# Neuroprotective Potential of *Sesamum indicum* in a Multifactorial In Vitro Neuron–Astrocyte System Exposed to Chronic Stress Mediators

**DOI:** 10.3390/biom16081084

**Published:** 2026-07-24

**Authors:** Viviana Soto-Mercado, María Paulina Arias-Loaiza, Miguel Mendivil-Perez

**Affiliations:** 1 Grupo de Neurociencias de Antioquia, Instituto de Investigaciones Médicas, Facultad de Medicina, Sede de Investigación Universitaria (SIU), Calle 62 No. 52-59, Torre 1, Laboratorios 411-412, Universidad de Antioquia (UdeA), Medellin 050010, Colombia; 2 Grupo de Investigación la Práctica de la Enfermería en el Contexto Social-GIPECS, Departamento Formación Básica Profesional, Facultad de Enfermería, Calle 67 #53-108, Universidad de Antioquia (UdeA), Medellin 050010, Colombia

**Keywords:** *Sesamum indicum*, sesamin, chronic stress, Alzheimer’s disease, neuroprotection

## Abstract

Chronic stress is increasingly recognized as a major contributor to Alzheimer’s disease (AD)-related neurodegeneration through mechanisms involving neuroinflammation, oxidative stress, mitochondrial dysfunction, excitotoxicity, and amyloidogenic processing. Here, we investigated the neuroprotective effects of Sesamum indicum whole paste extract (SIPE) using 2D neuron–astrocyte-like cell (ALC) co-cultures and 3D neuron–ALC spheroids exposed to a TNF-α/glutamate/cortisol (TGC) chronic stress paradigm. TGC exposure induced mitochondrial dysfunction, mitochondrial superoxide generation, astrocytic reactivity, NF-κB activation, reduced pro-BDNF expression, intracellular and extracellular Aβ42 accumulation, Tau phosphorylation, and caspase-3 activation, reproducing key hallmarks associated with chronic stress-related neurodegeneration. Among sesame-derived preparations evaluated, SIPE exhibited the strongest neuroprotective effects, preserving mitochondrial membrane potential, reducing oxidative stress, preventing neuronal loss, attenuating gliosis, and suppressing inflammatory, amyloidogenic, and apoptotic signaling. These effects were consistently reproduced in 3D spheroids. Phytochemical analysis revealed that SIPE contained the highest enrichment of sesamin, representing approximately 25% of the detected relative composition. Molecular docking analyses demonstrated favorable sesamin binding affinity toward TNF-α, DJ-1, Aβ42, and caspase-3. Collectively, these findings identify SIPE as a promising multitarget neuroprotective strategy against chronic stress-associated AD-related pathology.

## 1. Introduction

Chronic stress is characterized by sustained activation of the hypothalamic–pituitary–adrenal (HPA) axis and prolonged glucocorticoid exposure, ultimately leading to maladaptive neurobiological consequences. While acute stress promotes adaptive, survival-oriented responses, persistent cortisol elevation drives a progressive loss of homeostatic control across neuronal and glial compartments. This shift compromises neuronal plasticity, impairs mitochondrial bioenergetics, and amplifies oxidative and inflammatory cascades within stress-sensitive regions such as the hippocampus and prefrontal cortex [1,2,3]. Accordingly, chronic glucocorticoid signaling reshapes synaptic architecture, enhances excitatory transmission, and sensitizes neural circuits to inflammatory damage [4]. Increasing evidence further indicates that stress-induced immune activation—particularly via TNF-α, IL-1β, and NF-κB signaling—directly contributes to synaptic dysfunction, metabolic instability, and neuronal vulnerability [5]. Thus, chronic stress should be understood not merely as a neuroendocrine disturbance but as a systems-level condition capable of reprogramming neuron–glia interactions toward a neurodegenerative-prone phenotype.

Over the past decade, chronic psychosocial stress has emerged not simply as a comorbidity but as a potentially modifiable risk factor for dementia, especially Alzheimer’s disease (AD). Longitudinal and neuroimaging studies demonstrate that elevated cortisol levels correlate with accelerated hippocampal atrophy and progressive cognitive decline [6,7]. Mechanistically, glucocorticoid excess promotes amyloidogenic APP processing, increases Aβ aggregation, enhances tau phosphorylation through GSK3β activation, and exacerbates oxidative stress [8]. In parallel, neuroinflammation amplifies these pathological cascades by impairing Aβ clearance and promoting astrocyte–microglia reactivity [9]. Importantly, this stress–AD axis is also characterized by early impairment of neurotrophic support, particularly reduced brain-derived neurotrophic factor (BDNF) signaling. BDNF is essential for synaptic plasticity, dendritic spine maintenance, mitochondrial function, and neuronal survival, and its downregulation has been consistently reported in both chronic stress and AD brains [10,11]. Reduced BDNF signaling not only weakens synaptic resilience but also increases neuronal susceptibility to oxidative and inflammatory damage, suggesting that loss of neurotrophic support may represent an early mechanistic bridge between chronic stress and neurodegeneration. A second critical but often overlooked component of stress-related neurodegeneration is astrocytic metabolic dysfunction, particularly disruption of the glutamate–glutamine cycle. Astrocytes regulate extracellular glutamate levels through excitatory amino acid transporters and convert glutamate into glutamine via glutamine synthetase (GS), thereby preventing excitotoxicity and maintaining neurotransmitter cycling. This astrocyte-dependent metabolic coupling is essential for synaptic stability, antioxidant defense, and neuronal energy metabolism. However, chronic exposure to glucocorticoids and inflammatory cytokines has been shown to impair glutamate uptake and reduce glutamine synthetase activity, leading to extracellular glutamate accumulation, excitotoxic stress, and synaptic dysfunction [12,13]. Notably, decreased GS expression and activity have been reported in vulnerable brain regions in Alzheimer’s disease, suggesting that astrocytic metabolic failure may contribute to early synaptic vulnerability and disease progression [14]. Therefore, disruption of astrocytic glutamate detoxification and reduced BDNF support may represent convergent mechanisms through which chronic stress promotes neurodegenerative processes.

Accordingly, modeling chronic stress in vitro remains a significant conceptual challenge because stress-related neuropathology does not arise from a single molecular mediator but rather from the convergence of endocrine dysregulation, inflammatory activation, oxidative imbalance, and glutamatergic dysfunction. Oxidative stress represents a central integrative mechanism linking these pathological processes. While prolonged cortisol exposure is commonly used to mimic hypothalamic–pituitary–adrenal (HPA) axis hyperactivity and pro-inflammatory cytokines such as TNF-α or IL-1β are employed to reproduce inflammatory microenvironments, growing evidence indicates that glutamate dysregulation and astrocytic metabolic dysfunction are equally critical drivers of stress-induced neuronal vulnerability [15]. Importantly, many experimental models rely on exogenous hydrogen peroxide (H_2_O_2_) to induce oxidative damage; however, this approach only partially reproduces the physiological conditions of the brain, where H_2_O_2_ is generated endogenously through mitochondrial respiration, monoamine oxidase activity, peroxisomal metabolism, and inflammatory signaling. In neurons and astrocytes, intracellular H_2_O_2_ functions not only as a source of oxidative damage but also as a signaling molecule regulating NF-κB activation, mitochondrial dynamics, calcium homeostasis, and synaptic plasticity [16,17]. Among the intracellular targets of H_2_O_2_, the redox-sensitive protein DJ-1 (PARK7) has emerged as a critical oxidative stress sensor through the reversible oxidation of its catalytic Cys106 residue, enabling coordinated antioxidant, mitochondrial, and anti-inflammatory responses. Although DJ-1 was originally characterized for its neuroprotective role in Parkinson’s disease, growing evidence indicates that alterations in DJ-1 function contribute to the pathophysiology of a broad spectrum of neurodegenerative and neurological disorders, including Alzheimer’s disease and ischemic brain injury [18,19,20,21,22,23,24]. Consequently, DJ-1 is increasingly recognized as a central molecular hub linking oxidative stress, mitochondrial dysfunction, neuroinflammation, and neuronal survival across diverse brain disorders.

Moreover, early stress-related synaptic dysfunction appears to involve subtle alterations in glutamate cycling, glutamine synthetase activity, mitochondrial function, and BDNF signaling before overt neuronal loss becomes evident [25]. In this context, astrocytes are not passive bystanders but active regulators of synaptic homeostasis, redox balance, cytokine signaling, and neurotrophic support. Stress-induced astrocytic reactivity can impair glutamate clearance, amplify TNF-α signaling, disrupt GS-dependent metabolism, and destabilize synaptic integrity in a coordinated manner [26]. Consequently, experimental paradigms that isolate individual stress mediators or rely exclusively on exogenous oxidants may fail to recapitulate the spatial, temporal, and mechanistic complexity of chronic stress. This reductionist approach may partly explain why the transition from chronic stress exposure to Alzheimer’s disease-like molecular alterations remains incompletely understood. Therefore, determining whether a multifactorial model combining endocrine stress (cortisol), inflammatory stress (TNF-α), and excitatory stress (glutamate dysregulation) can induce endogenous oxidative stress generation within neuron–astrocyte systems represents an important step toward developing experimental conditions that more closely resemble the in vivo brain microenvironment.

In Colombia, multiple rural and peri-urban communities have endured prolonged exposure to armed conflict, displacement, and socioeconomic instability, resulting in sustained psychosocial stress burdens across vulnerable populations [27,28,29]. Nevertheless, community resilience has emerged through collective practices rooted in cultural identity, including traditional food cultivation and transformation. Among these, *Sesamum indicum* (sesame) plays nutritional, economic, and symbolic roles in several regions [30]. The artisanal processing of sesame into oils and pastes not only strengthens local bioeconomies but may also provide bioactive compounds with neuroprotective potential [31]. Sesame seeds are rich in lignans such as sesamin and sesamol, tocopherols, polyphenols, and unsaturated fatty acids with documented antioxidant and anti-inflammatory properties [32]. Experimental studies demonstrate that sesame lignans suppress NF-κB signaling, reduce TNF-α production, improve mitochondrial function, and attenuate oxidative stress [33]. Some reports also suggest that antioxidant and anti-inflammatory compounds can indirectly restore BDNF signaling and preserve astrocytic metabolic function, including glutamate clearance pathways [34]. Accordingly, it is important to determine whether the bioactive properties associated with sesame may contribute to neuroprotection in experimental models of chronic stress.

In this context, we propose that the combined exposure to TNF-α, cortisol, and glutamate in a neuron–astrocyte co-culture establishes a multifactorial stress paradigm that integrates inflammatory, endocrine, and excitotoxic insults frequently associated with chronic stress. Rather than recapitulating the full complexity of chronic stress, this approach was designed to reproduce convergent molecular mechanisms implicated in stress-related neurodegeneration and endogenous oxidative stress generation. We hypothesize that this multifactorial stress paradigm will promote Alzheimer’s disease-associated molecular alterations in vitro, including increased Aβ production, Tau hyperphosphorylation, oxidative imbalance, mitochondrial dysfunction, impaired BDNF signaling, disruption of astrocytic glutamine synthetase-dependent metabolism, and neuronal cell death. Furthermore, we propose that transformed *S. indicum* seed-derived preparations will attenuate inflammatory signaling, preserve redox homeostasis, restore glutamatergic balance, and protect neuron–astrocyte metabolic coupling against multifactorial stress-induced injury.

Taken together, this integrative approach addresses a critical gap in current experimental models by incorporating endocrine, inflammatory, excitatory, and oxidative components into a single mechanistic framework. By linking stress biology, astrocyte metabolism, neurotrophic signaling, and culturally relevant functional foods, this study provides a translational model for understanding how chronic stress may contribute to neurodegenerative disease and how community-based nutritional strategies may offer neuroprotective potential.

## 2. Materials and Methods

### 2.1. Neuronal Differentiation

Neuronal differentiation was performed from neural precursor cells (NPCs) as previously described [35], with minor modifications. NPCs were maintained in proliferation medium consisting of Neurobasal medium (Cat# 21103049, Gibco, Santa Fe, NM, USA) supplemented with 1% N2 supplement (Cat# 17502048, Gibco, Santa Fe, NM, USA), 2% B27 supplement (Cat# 17504044, Gibco, Santa Fe, NM, USA), 20 ng/mL epidermal growth factor (EGF; Cat# E5036, Sigma-Aldrich, St. Louis, MO, USA), 1 µg/mL heparin sodium salt (Cat# 375095, Sigma-Aldrich, St. Louis, MO, USA), 1 ng/mL basic fibroblast growth factor (bFGF; Cat# F0291-25UG, Sigma-Aldrich, St. Louis, MO, USA), 1× β-mercaptoethanol (Cat# M3148-100ML, Sigma-Aldrich, St. Louis, MO, USA), and 1% penicillin/streptomycin (Cat# 15140122, Gibco, Santa Fe, NM, USA). For neuronal differentiation, NPCs were plated at a density of 2 × 10^4^ cells/cm^2^ in culture plates and maintained in NPC proliferation medium for 4 days to allow cell attachment and stabilization. After this period, the proliferation medium was replaced with cholinergic differentiation medium (Cholinergic-N-Run) as previously described [36,37], and cells were maintained at 37 °C for 15 days to promote cholinergic neuronal differentiation. Following the differentiation period, the medium was replaced with a minimal maintenance medium consisting of DMEM low glucose with sodium bicarbonate, sodium pyruvate, and L-glutamine (Cat# D6046, Sigma-Aldrich, St. Louis, MO, USA) supplemented with 1% fetal bovine serum (FBS; Cat# 10609220, Gibco, Santa Fe, NM, USA), which was used for subsequent experimental procedures.

### 2.2. Astrocyte-like Cells (ALCs) Differentiation

Astrocytic differentiation was induced as previously described by [38], with minor modifications. Wharton’s Jelly-derived mesenchymal stem cells (WJ-MSCs) were plated at a density of 1 × 10^4^ cells/cm^2^ in culture flasks containing DMEM low glucose with sodium bicarbonate, sodium pyruvate, and L-glutamine (Cat. No. D6046, Sigma-Aldrich, St. Louis, MO, USA) supplemented with 10% fetal bovine serum (FBS; Cat. No. 10609220, Gibco, Santa Fe, NM, USA). Cells were allowed to proliferate until reaching approximately 40% confluence (4 days). The culture medium was then replaced with Astrocyte Medium^®^ (Cat. No. 1801, ScienCell Research Laboratories, Carlsbad, CA, USA), and cells were maintained under these conditions for 8 days to induce astrocytic differentiation, following the manufacturer’s instructions.

### 2.3. Neuron–ALCs Co-Culture

For co-culture experiments, neuronal cells were first differentiated from neural precursor cells as described above and allowed to reach the desired maturation stage prior to co-culture establishment. ALCs were differentiated separately from WJ-MSCs as previously described. Once astrocytic differentiation was completed, astrocytes were detached using 0.25% trypsin (Cat. No. T4799-25G, Sigma-Aldrich, St. Louis, MO, USA), centrifuged, and resuspended in neural maintenance medium. Co-cultures were established by seeding astrocytes together with previously differentiated neurons at a neuron-to-ALCs ratio of 3:1, reaching a final total cell density of 5 × 10^4^ cells/cm^2^. Cells were maintained in neural maintenance medium composed of DMEM low glucose with sodium bicarbonate, sodium pyruvate, and L-glutamine (Cat. No. D6046, Sigma-Aldrich, St. Louis, MO, USA) supplemented with 1% fetal bovine serum (FBS; Cat. No. 10609220, Gibco, Santa Fe, NM, USA) under standard culture conditions (37 °C, 5% CO_2_). Co-cultures were allowed to stabilize for 48 h before initiating experimental treatments.

### 2.4. Three-Dimensional Co-Culture Spheroids of NPCs and Astrocyte-like Cells (ALCs)

Three-dimensional co-culture spheroids were generated using neural precursor cells (NPCs) and astrocyte-like cells (ALCs) derived as previously described. For spheroid formation, cells were cultured in Fast-N-Spheres V2 medium, a serum-free culture medium supplemented with the reagents previously described [39], additionally containing Growth Factor Reduced Matrigel^®^ Basement Membrane Matrix (Cat. No. 354230, Corning, Corning, NY, USA) and 1% fetal bovine serum (FBS; Cat. No. 10609220, Gibco, Santa Fe, NM, USA) to promote three-dimensional aggregation and cell–cell interaction. NPCs and ALCs were mixed prior to seeding to establish the co-culture system. Cells (1.4 × 10^5^ cells/cm^2^; 3:1 NPCs:ALCs) were seeded in suspension culture conditions and maintained under constant agitation to promote spheroid formation and prevent cell adhesion to the culture surface. Cultures were maintained under orbital agitation for 15 days, and the culture medium was partially replaced every 5 days to maintain nutrient availability and remove metabolic waste. Under these conditions, cells spontaneously formed three-dimensional co-culture spheroids. The resulting spheroids were subsequently evaluated for the expression of neuronal and astrocytic markers and used for downstream experimental analyses.

### 2.5. In Vitro Chronic Stress-Associated Cellular Environment Reagents

Recombinant human tumor necrosis factor alpha (TNF-α) was purchased from Sino Biological Inc. (Cat. No. 10602-HNAE, Beijing, China). Cortisol (hydrocortisone; Cat. No. H0888, Sigma-Aldrich, St. Louis, MO, USA) and L-glutamate (Cat. No. G5889, Sigma-Aldrich, St. Louis, MO, USA) were obtained from Sigma-Aldrich (St. Louis, MO, USA). Stock solutions were prepared under sterile conditions according to the manufacturers’ instructions and stored at the recommended temperatures until use. Working solutions were freshly prepared in culture medium immediately before each experiment. These reagents were used to reproduce the inflammatory (TNF-α), endocrine (cortisol), and excitatory (glutamate) components of chronic stress in the neuron–astrocyte co-culture model.

### 2.6. Sesame-Derived Products and Preparation of Crude Ethanolic Extracts

Sesame-derived products (*n* = 3) were obtained from small-scale farmers located in the Montes de María region in northern Colombia, a region characterized by traditional agricultural practices and artisanal processing methods that have been preserved within local communities for generations. The products included raw sesame seeds and sesame paste obtained through traditional transformation processes carried out by local farmers using ancestral preparation methods. These processes typically involve manual seed selection, roasting, grinding, and mechanical extraction performed under non-industrial conditions. Briefly, sesame seeds are manually selected and roasted using traditional artisanal methods at approximately 170 ± 20 °C for 15 ± 5 min, followed by mechanical grinding to obtain a homogeneous whole paste while preserving the natural oil fraction and bioactive phytochemicals of the seeds. All samples were collected directly from local producers to ensure traceability and to preserve the physicochemical characteristics associated with traditional processing methods (Supplementary Figure S1). Samples were transported under controlled conditions to the laboratory for subsequent processing and experimental evaluation. For extract preparation, seed macerate, sesame paste and oil samples (10 mg each) were dissolved in 90% ethanol (*v*/*v*) using a solid-to-solvent ratio of 1:10 (*w*/*v*) and incubated for 24 h at room temperature under maceration conditions. The resulting mixtures were then sterilized by filtration through 0.22 µm syringe filters (Cat. No. 431224, Corning Incorporated, Corning, NY, USA), and the filtrates were collected as crude ethanolic extracts. No solvent evaporation or concentration step was performed in order to preserve the composition of the crude extracts. Extracts were stored at 4 °C until use. For in vitro experiments, the final ethanol concentration in the culture medium did not exceed 1%, and corresponding vehicle controls were included in all experiments. The crude ethanolic extracts obtained from these materials were used exclusively for the biological experiments, whereas independent aliquots of the original sesame-derived products were subjected to GC–MS chemical characterization as described below.

### 2.7. Determination of Total Polyphenol Content

Total polyphenol content was determined using the AOAC Official Method 2017.13 based on the Folin–Ciocalteu colorimetric assay. Sesame-derived products, including sesame seed, sesame paste, and sesame oil, were analyzed. Approximately 250 mg of each solid sample was extracted with 1.8 mL of 70% ethanol using ultrasound-assisted extraction. Samples were subsequently centrifuged at 13,000 rpm for 10 min, and the supernatant was adjusted to a final volume of 2 mL with 70% ethanol. Dilutions (1:2.5) were prepared using distilled water as solvent. Sesame oil samples were directly analyzed without prior extraction. Polyphenols were quantified spectrophotometrically at 765 nm using gallic acid as the reference standard. Results were expressed as mg gallic acid equivalents (GAE) per 100 g of sample. Calibration curves were constructed using gallic acid standards, and quantification was performed according to the linear regression equation obtained from the standard curve.

### 2.8. GC–MS Chemical Profiling

To chemically characterize the sesame-derived materials used for extract preparation, representative samples of sesame seed, whole sesame paste, and sesame oil were independently analyzed by GC–MS. This analysis was performed to determine the relative phytochemical composition of each starting material used for the preparation of the crude ethanolic extracts employed in the biological assays. Approximately 2.0 g of each sample was subjected to double extraction with 20 mL of ethyl acetate under ultrasonic conditions for 45 min. The solvent was removed under reduced pressure, and approximately 25 mg of the dried extract were derivatized with BSTFA and pyridine at 70 °C for 2 h prior to chromatographic analysis. GC–MS analyses were performed using an Agilent 7890 gas chromatograph coupled to an MSD 5975C mass spectrometer equipped with an HP-5MS capillary column (30 m × 0.25 mm i.d., 0.25 μm film thickness). Helium was used as the carrier gas at a constant flow rate of 1.0 mL/min. The oven temperature program was set as follows: 120 °C for 5 min, increased at 7 °C/min to 180 °C and held for 5 min, then increased at 10 °C/min to 200 °C and held for 5 min, followed by an increase to 300 °C at 10 °C/min and held for 5 min, and finally increased to 310 °C at 10 °C/min and maintained for 10 min. Data acquisition was performed in SCAN mode, and metabolite identification was achieved by comparison of mass spectra with the NIST23 spectral library using MassHunter software (accessed on 15 March 2026; Agilent Technologies, Santa Clara, CA, USA. Version 10.0). Relative abundances of the identified metabolites were calculated as chromatographic peak-area percentages. The complete phytochemical composition, including retention times, CAS numbers, relative peak-area percentages, and unidentified chromatographic peaks detected in sesame seed, whole sesame paste, and sesame oil, was represented in tables. This chemical characterization was performed independently of the biological experiments and was intended exclusively to compare the phytochemical composition of the three sesame-derived products evaluated in this study.

### 2.9. Fatty Acid Methyl Ester (FAME) Profiling by GC–MS

The fatty acid composition of *Sesamum indicum* seed, oil, and whole paste samples was determined by gas chromatography–mass spectrometry (GC–MS) following acid-catalyzed transesterification of lipids into fatty acid methyl esters (FAMEs). Briefly, approximately 15 mg of each sample was weighed and mixed with 1 mL of 2% sulfuric acid in methanol. The transesterification reaction was carried out at 70 °C for 3 h. Subsequently, FAMEs were extracted with hexane, transferred to a 2 mL volumetric flask, and diluted 1:10 prior to chromatographic analysis. GC–MS analyses were performed using an Agilent 7890 gas chromatograph coupled to an MSD 5975C mass spectrometer (Agilent Technologies, Santa Clara, CA, USA), equipped with a ZB-WAX Plus capillary column (30 m × 0.25 mm internal diameter × 0.25 μm film thickness; Phenomenex, Torrance, CA, USA). Helium was used as carrier gas at a constant flow rate of 0.75 mL/min. The oven temperature program was as follows: initial temperature of 100 °C, increased to 130 °C at 10 °C/min, then to 200 °C at 3 °C/min, followed by 205 °C at 1 °C/min, and finally to 225 °C at 0.7 °C/min with a final hold of 5 min. Mass spectra were acquired in SCAN mode, and compound identification was performed by comparison with a commercial FAME standard mixture and the NIST23 spectral library using MassHunter software (Accessed on 19 March 2026; Agilent Technologies, Santa Clara, CA, USA. Version 10.0). Relative abundance of each fatty acid was calculated as percentage of chromatographic peak area.

### 2.10. Experimental Treatment Protocol

For experiments performed in the 2D neuron–astrocyte co-culture system, a dose–response curve was first established to evaluate the effect of the combined stress treatment consisting of tumor necrosis factor alpha (TNF-α), glutamate, and cortisol (TGC). Cells were exposed for 4 days to the following TGC combinations: 0:0:0, 10:1:10, 25:2.5:25, 50:5:50, 100:10:100, 500:50:500, and 1000:100:1000, corresponding to TNF-α (ng/mL), glutamate (µM), and cortisol (nM), respectively. Based on the dose–response results, the 100:10:100 condition was selected for subsequent experiments, as it induced significant cellular alterations without causing complete loss of viability. After selecting this condition, co-cultures were pretreated for 24 h with either vehicle (1% ethanol) or crude ethanolic extracts obtained from *Sesamum indicum*-derived products, including seeds, whole sesame paste and oil. Extracts were tested at final concentrations of 0, 1, 10, 100, and 1000 µg/mL. Following pre-treatment, cells were exposed to the selected TGC condition (100:10:100) for 4 days. At the end of the treatment period, cells were processed for the corresponding experimental analyses. For the 3D co-culture model, cells were treated only with the selected TGC condition (100:10:100) and the sesame paste extract at a final concentration of 10 µg/mL, based on the results obtained in the 2D model.

### 2.11. Cell Viability Assay

Cell viability was assessed using the 3-(4,5-dimethylthiazol-2-yl)-2,5-diphenyl tetrazolium bromide reduction assay (MTT, Cat. No. M5655, Sigma-Aldrich, St. Louis, MO, USA) as an indicator of mitochondrial metabolic activity. For the initial dose–response analysis of the combined stress condition, 2D neuron–astrocyte co-cultures were exposed for 4 days to different combinations of tumor necrosis factor alpha (TNF-α), glutamate, and cortisol (TGC): 0:0:0, 10:1:10, 25:2.5:25, 50:5:50, 100:10:100, 500:50:500, and 1000:100:1000, corresponding to TNF-α (ng/mL), glutamate (µM), and cortisol (nM), respectively. Based on these results, the 100:10:100 condition was selected for subsequent experiments. To evaluate the effects of *Sesamum indicum* extracts, co-cultures were pretreated for 24 h with crude ethanolic extracts obtained from seeds, whole sesame paste and oil at final concentrations of 0, 1, 10, 100, and 1000 µg/mL. After pre-treatment, cells were incubated for 4 days either in the absence or in the presence of the selected TGC combination (100:10:100), allowing the evaluation of both the intrinsic cytotoxicity of the extracts and their potential protective effects under stress conditions. At the end of the treatment period, culture medium was removed, and cells were incubated with MTT solution (0.5 mg/mL final concentration) for 3 h at 37 °C. The supernatant was then removed, and the resulting formazan crystals were dissolved in dimethyl sulfoxide (DMSO). Absorbance was measured at 570 nm using a microplate reader. Cell viability was expressed as neural cell mitochondrial activity in arbitrary units (AU), corresponding to the absorbance values obtained in the MTT assay.

### 2.12. Superoxide Production Assay

Superoxide production was assessed using the Nitro Blue Tetrazolium reduction assay (NBT, Cat. No. N6876, Sigma-Aldrich, St. Louis, MO, USA), which measures the intracellular production of superoxide anion (O_2_^−^) based on the reduction of NBT to insoluble formazan crystals. For the initial dose–response analysis, 2D neuron–astrocyte co-cultures were exposed for 4 days to different combinations of tumor necrosis factor alpha (TNF-α), glutamate, and cortisol (TGC): 0:0:0, 10:1:10, 25:2.5:25, 50:5:50, 100:10:100, 500:50:500, and 1000:100:1000, corresponding to TNF-α (ng/mL), glutamate (µM), and cortisol (nM), respectively. Based on these results, the 100:10:100 condition was selected for subsequent experiments. To evaluate the effects of *Sesamum indicum* extracts on oxidative stress, co-cultures were pretreated for 24 h with crude ethanolic extracts obtained from seeds, whole sesame paste or oil at final concentrations of 0, 1, 10, 100, and 1000 µg/mL. After pre-treatment, cells were incubated for 4 days either in the absence or in the presence of the selected TGC combination (100:10:100). At the end of the treatment period, cells were incubated with NBT solution (1 mg/mL) for 1 h at 37 °C. After incubation, the medium was removed, and the reduced formazan deposits were dissolved using DMSO. Absorbance was measured at 560 nm using a microplate reader. Neural cell superoxide production was expressed as neural cell superoxide production in arbitrary units (AU), corresponding directly to the absorbance values obtained from the NBT reduction assay.

### 2.13. Flow Cytometric Analysis of Mitochondrial Membrane Potential Using DiOC_6_(3)

Mitochondrial membrane potential (ΔΨm) was evaluated using 3,3′-dihexyloxacarbocyanine iodide (DiOC_6_(3), Cat. No. D-273, Calbiochem, Darmstadt, Germany), a lipophilic fluorescent dye that accumulates in mitochondria in a membrane potential–dependent manner and is commonly used as an indicator of mitochondrial polarization. Neuron–astrocyte co-cultures were maintained under control conditions (vehicle, 1% ethanol; see Experimental Treatment Protocol above), treated with sesame paste extract (10 µg/mL), exposed to the selected stress condition TGC (100:10:100; TNF-α ng/mL:glutamate µM:cortisol nM), or treated with the combination of sesame paste extract (10 µg/mL) and TGC (100:10:100). After the treatment period, cells were incubated with DiOC_6_(3) at a final concentration of 20 nM for 20 min at 37 °C in the dark. Cells were then washed with phosphate-buffered saline (PBS), detached using trypsin, collected, and resuspended in PBS for flow cytometry analysis. Unstained cells were used as staining controls to establish background fluorescence and gating parameters. Flow cytometric analysis was performed using a BD LSRFortessa™ II flow cytometer (BD Biosciences, San Jose, CA, USA). Twenty thousand events were acquired per sample, and data were analyzed using FlowJo 7.6.2 Data Analysis Software. Changes in mitochondrial membrane potential were expressed as the percentage of DiOC_6_(3) low cells.

### 2.14. Flow Cytometric Analysis of Mitochondrial Superoxide Production Using MitoSOX Red

Mitochondrial superoxide production was evaluated using MitoSOX™ Red Mitochondrial Superoxide Indicator (Cat. No. M36007, Invitrogen™, Thermo Fisher Scientific, Waltham, MA, USA), a fluorogenic probe selectively targeted to mitochondria and oxidized primarily by superoxide. Neuron–astrocyte co-cultures were maintained under control conditions (vehicle, 1% ethanol; see Experimental Treatment Protocol above), treated with sesame paste extract (10 µg/mL), exposed to the selected stress condition TGC (100:10:100; TNF-α ng/mL:glutamate µM:cortisol nM), or treated with the combination of sesame paste extract (10 µg/mL) and TGC (100:10:100). After the treatment period, cells were incubated with MitoSOX Red at a final concentration of 100 nM for 20 min at 37 °C under dark conditions. Cells were then washed with phosphate-buffered saline (PBS), trypsinized, collected, and resuspended in PBS for flow cytometry analysis. Unstained cells were included as staining controls to establish background fluorescence and gating parameters. Flow cytometric analysis was performed using a BD LSRFortessa™ II flow cytometer. Twenty thousand events were acquired per sample, and data were analyzed using FlowJo 7.6.2 Data Analysis Software. Mitochondrial superoxide production was expressed as the percentage of MitoSOX-positive cells.

### 2.15. Triple Fluorescence Labeling of Co-Cultures with Hoechst 33342, DiOC_6_(3), and MitoSOX Red

For the simultaneous assessment of nuclear morphology, mitochondrial membrane potential, and mitochondrial superoxide production, neuron–astrocyte co-cultures were stained with Hoechst 33342 (Cat. No. B2261, Sigma-Aldrich, St. Louis, MO, USA), DiOC_6_(3), and MitoSOX Red. Co-cultures were maintained under control conditions (vehicle, 1% ethanol; see Experimental Treatment Protocol above), treated with sesame paste extract (10 µg/mL), exposed to the selected stress condition TGC (100:10:100; TNF-α ng/mL:glutamate µM:cortisol nM) alone or in combination. Following the treatment period, cells were incubated for 20 min at 37 °C in the dark with Hoechst 33342 (Thermo Fisher Scientific; Cat. No. H3570) at a final concentration of 1 µM, DiOC_6_(3) at a final concentration of 20 nM, and MitoSOX™ Red Mitochondrial Superoxide Indicator (Invitrogen™, Thermo Fisher Scientific; Cat. No. M36007) at a final concentration of 100 nM. After staining, cells were washed with phosphate-buffered saline (PBS) and immediately analyzed by fluorescence microscopy. Hoechst fluorescence was used for nuclear identification, DiOC_6_(3) signal for mitochondrial membrane potential assessment, and MitoSOX fluorescence for mitochondrial superoxide detection. Fluorescence microscopy photographs were taken using a Zeiss Axiovert A1 Fluorescence Microscope equipped with a Zeiss AxioCam Cm1 and Zeiss Wöhlk-Contact-Linsfluoreen (GmbH Schcönkirchen, Göttingen, Germany). Fluorescence images were analyzed using ImageJ software (version 1.53k; National Institutes of Health, Bethesda, MD, USA; http://imagej.nih.gov/ij/ (accessed on 11 November 2025)).

### 2.16. Immunofluorescence Analysis by Flow Cytometry

Flow cytometry immunofluorescence analysis was performed to evaluate neuronal, astrocytic, neurodegenerative, oxidative stress, inflammatory, and apoptotic markers in neuron–astrocyte co-cultures [40]. Co-cultures were maintained under control conditions (vehicle, 1% ethanol; see Experimental Treatment Protocol above), treated with sesame paste extract (10 µg/mL), exposed to the selected stress condition TGC (100:10:100; TNF-α ng/mL:glutamate µM:cortisol nM), or treated with the combination of sesame paste extract (10 µg/mL) and TGC (100:10:100). After the treatment period, cells were detached using trypsin, washed with phosphate-buffered saline (PBS), and centrifuged at 1500 rpm for 5 min. Cells were then resuspended in PBS and fixed with cold 70% ethanol overnight at 4 °C. After fixation, cells were washed twice with PBS and incubated with primary antibodies diluted 1:500 in PBS containing 1% Bovine Serum Albumin (BSA; Cat. No. A9647, Sigma-Aldrich, St. Louis, MO, USA) for 1 h at room temperature. The following primary antibodies were used: βIII-tubulin (neuronal marker), GFAP (astrocyte marker), pro-BDNF, glutamine synthetase (GS), amyloid-β (Aβ), oxidized DJ-1 (ox(Cys106)DJ-1; PARK7), Tau (Ser404), NF-κB (p65), and cleaved caspase-3 (Table 1). After primary antibody incubation, cells were washed with PBS and incubated with the corresponding fluorescent secondary antibodies for 1 h at room temperature in the dark. Finally, cells were washed, resuspended in PBS, and analyzed using a BD LSRFortessa™ II flow cytometer. Ten thousand events were acquired per sample, and data were analyzed using FlowJo 7.6.2 Data Analysis Software. Results were expressed as the percentage of positive cells and mean fluorescence intensity (MFI) for each marker.

### 2.17. Immunofluorescence Analysis by Fluorescence Microscopy

Fluorescence microscopy immunofluorescence analysis was performed to evaluate neuronal, astrocytic, neurodegenerative, oxidative stress, inflammatory, and apoptotic markers in neuron–astrocyte co-cultures. Co-cultures were seeded on glass coverslips placed in 24-well plates and maintained under control conditions (vehicle, 1% ethanol; see Experimental Treatment Protocol above), treated with sesame paste extract (10 µg/mL), exposed to the selected stress condition TGC (100:10:100; TNF-α ng/mL:glutamate µM:cortisol nM), or treated with the combination of sesame paste extract (10 µg/mL) and TGC (100:10:100). After the treatment period, cells were washed twice with PBS and fixed with 4% paraformaldehyde for 20 min at room temperature. Subsequently, cells were washed three times with PBS and permeabilized with 0.2% Triton™ X-100 (Cat. No. T8787, Sigma-Aldrich, St. Louis, MO, USA) in PBS for 10 min. Non-specific binding sites were blocked by incubating the cells with 5% BSA in PBS for 1 h at room temperature. Cells were then incubated overnight at 4 °C with primary antibodies diluted 1:500 in PBS containing 1% BSA. The following primary antibodies were used: βIII-tubulin (neuronal marker), GFAP (astrocyte marker), pro-BDNF, glutamine synthetase (GS), amyloid-β (Aβ; clone 6E10), oxidized DJ-1 (ox(Cys106)DJ-1; PARK7), Tau (Ser404), NF-κB (p65), and cleaved caspase-3 (see above). After primary antibody incubation, cells were washed three times with PBS and incubated for 1 h at room temperature in the dark with the corresponding species-specific secondary antibodies conjugated to Alexa Fluor™ 488 Goat Anti-Rabbit IgG (H+L) (Cat. No. A-11008, Invitrogen™, Thermo Fisher Scientific, Waltham, MA, USA) or Alexa Fluor™ 594 Goat Anti-Mouse IgG (H+L) (Cat. No. A-11005, Invitrogen™, Thermo Fisher Scientific, Waltham, MA, USA) (1:500 dilution). Subsequently, nuclei were stained with Hoechst 33342 (0.5 µM) for 10 min at room temperature. Coverslips were mounted onto glass slides using fluorescence mounting medium and allowed to dry in the dark. Fluorescence images were acquired using a Zeiss Axiovert A1 equipped with a Zeiss AxioCam CM1 (see above). For each experimental condition, at least five random fields per coverslip were captured under identical exposure settings. Fluorescence intensity and marker colocalization were analyzed using ImageJ (NIH, USA). Results were expressed as relative fluorescence intensity.

### 2.18. Cytokine Release Measurement (IL-1β and IL-6)

Neuron–astrocyte co-cultures were seeded in 24-well plates as described previously. Cells were maintained under control conditions (vehicle, 1% ethanol; see Experimental Treatment Protocol above), treated with sesame paste extract (10 µg/mL), exposed to the selected stress condition TGC (100:10:100; TNF-α ng/mL:glutamate µM:cortisol nM), or treated with the combination of sesame paste extract (10 µg/mL) and TGC (100:10:100). After the treatment period, culture supernatants were collected, centrifuged at 1500 rpm for 5 min to remove cellular debris, and stored at −80 °C until further analysis. The secretion levels of IL-1β and IL-6 were quantified in cell-free supernatants using the Cytometric Bead Array (CBA) Human Inflammatory Cytokine Kit (Cat. No. 551811, BD Biosciences, San Jose, CA, USA) [41], following the manufacturer’s instructions. Briefly, samples were incubated with cytokine-specific capture beads and phycoerythrin (PE)-conjugated detection antibodies, allowing the formation of fluorescence-labeled bead–cytokine complexes. Data acquisition was performed using a CytoFLEX Flow Cytometer (Beckman Coulter Life Sciences, Indianapolis, IN, USA). At least 3000 events per analyte were recorded, and cytokine concentrations were determined based on standard curves generated using recombinant cytokine standards provided in the kit. Results were expressed as pg/mL of IL-1β and IL-6 in culture supernatants.

### 2.19. Measurement of Aβ1–42 Peptides in Culture Supernatants

The levels of secreted Aβ1–42 peptides were quantified in neuron–astrocyte co-cultures under different experimental conditions. Cells were maintained under control conditions (vehicle, 1% ethanol; see Experimental Treatment Protocol above), treated with sesame paste extract (10 µg/mL), exposed to the selected stress condition TGC (100:10:100; TNF-α ng/mL:glutamate µM:cortisol nM), or treated with the combination of sesame paste extract (10 µg/mL) and TGC (100:10:100). After the treatment period, culture supernatants were collected and centrifuged at 1500 rpm for 5 min to remove cellular debris. Aliquots (100 µL) of cell-free supernatants were used for the quantification of Aβ1–42 peptide levels. Aβ1–42 concentrations were determined using a human Aβ1–42 solid-phase sandwich ELISA kit (Cat. No. KHB3441, Invitrogen™, Thermo Fisher Scientific, Waltham, MA, USA) [35], following the manufacturer’s instructions. Briefly, samples and standards were added to wells pre-coated with an anti-Aβ antibody, followed by incubation with a detection antibody specific for Aβ1–42. After washing steps, a substrate solution was added, and absorbance was measured at 450 nm using a Multiskan SkyHigh Microplate Spectrophotometer (Thermo Fisher Scientific, Vantaa, Finland). Aβ1–42 concentrations were calculated based on standard curves generated with known concentrations of recombinant peptide and expressed as pg/mL of culture supernatant. All measurements were performed in triplicate in three independent experiments, and analyses were conducted blind to the experimental conditions.

### 2.20. Real-Time Quaking-Induced Conversion (RT-QuIC) Assay (Cell-Free System)

Amyloid aggregation kinetics were evaluated using a cell-free Real-Time Quaking-Induced Conversion (RT-QuIC) assay with minor modifications to previously described protocols [42]. Synthetic Aβ1–42 peptide (Aβ42 peptide ab120301, Abcam) was dissolved in DMSO (Sigma-Aldrich) and sonicated for 5 min immediately prior to use to ensure monomerization. For the first experimental setup, Aβ42 peptide (100 µg/mL) was incubated alone or in the presence of sesame paste extract (SIPE, 10 µg/mL). For the second experimental setup, a synthetic DJ-1 peptide was combined with Aβ42 (100 µg/mL) and hydrogen peroxide (H_2_O_2_, 10 µM) to induce oxidative conditions, in the absence or presence of SIPE (10 µg/mL). All reactions were prepared in PBS (pH 7.4) containing Congo Red (CR, 10 µM) as an amyloid-sensitive dye, with a final reaction volume of 100 µL per well. Each condition was run in multiple technical replicates in a 96-well plate. Plates were incubated at 37 °C in a Multiskan SkyHigh Plate Reader (See above) under intermittent shaking conditions (600 rpm for 1 min every 60 min) for a total of 48 h to promote amyloid fibril formation. Absorbance readings were recorded every 60 min at 420 nm and 540 nm to monitor Congo Red binding shifts associated with amyloid aggregation. Aggregation kinetics were analyzed by calculating changes in absorbance ratios over time, allowing the assessment of fibril formation dynamics under each experimental condition.

### 2.21. Dot-Blot Analysis of DJ-1 Oxidation

The effect of sesame paste extract (SIPE) on DJ-1 oxidation was evaluated using a cell-free dot-blot assay as described previously [43]. Briefly, synthetic DJ-1 peptide was prepared at a final concentration of 5 µM in phosphate-buffered saline (PBS, pH 7.4). The peptide was pre-incubated in the presence or absence of SIPE (10 µg/mL) for 10 min at 37 °C. Following pre-incubation, samples were exposed to hydrogen peroxide (H_2_O_2_, 10 µM) for 10 min at 37 °C to induce oxidative modification of DJ-1 at cysteine 106. After treatment, equal volumes of each reaction mixture were spotted onto nitrocellulose membranes (Hybond™-ECL Nitrocellulose Membrane, Cat. No. RPN303D, Amersham Biosciences, Little Chalfont, Buckinghamshire, UK) and allowed to dry at room temperature. Membranes were then blocked with 5% bovine serum albumin (BSA) in PBS for 1 h at room temperature to prevent non-specific binding. Subsequently, membranes were incubated overnight at 4 °C with a primary antibody against oxidized DJ-1 (ox(Cys106)DJ-1, see above), diluted 1:5000 in PBS containing 1% BSA. This antibody specifically recognizes DJ-1 oxidized at cysteine 106 to cysteine sulfonic acid (SO_3_H), a marker of irreversible oxidative stress. After primary incubation, membranes were washed with PBS and incubated for 1 h at room temperature with an infrared secondary antibody: goat anti-rabbit IgG IRDye^®^ 680RD secondary antibody (Cat. No. 926-68071, LI-COR Biosciences, Lincoln, NE, USA) diluted 1:1000 in blocking buffer. Signal detection and densitometric analysis were performed using the Odyssey imaging system and software (LI-COR Biosciences. Image Studio Software. Lincoln, NE, USA. Version 6.2). Relative signal intensity was quantified and normalized to control conditions. All experiments were performed in triplicate in three independent assays.

### 2.22. Molecular Docking

Molecular docking analyses were performed using the three-dimensional structures of tumor necrosis factor alpha (TNF-α), DJ-1, caspase-3, and amyloid beta 1–42 (Aβ42). The protein structures of TNF-α, DJ-1, and caspase-3 were obtained from the Protein Data Bank (PDB codes: 6RMJ, 2OR3, and 1NME, respectively), whereas the Aβ42 structure was generated using AlphaFold2. Blind molecular docking was carried out using CB-Dock3, an improved cavity-detection-guided protein–ligand blind docking web server that integrates cavity prediction with AutoDock Vina 1.2.0. The SDF structure files of the tested ligands, including sesamin (CID: 72307), sesamolin (CID: 101746), (−)-beta-Sitosterol (CID: 222284), campesterol (CID: 173183), (+)-gamma-Tocopherol (CID: 92729) as well as reference compounds for each protein target (TNF-α inhibitor (CID: 16079006), 8RK64 (CID: 162677482), thioflavin T (CID: 16953), and NSCI (CID: 11591540)), were downloaded from PubChem. For each docking assay, the corresponding 3D structure file of each target protein was uploaded to the CB-Dock3 server together with the SDF file of each ligand. Docking poses were ranked according to their Vina score, and the best-scoring pose located within the predicted functional or binding cavity was selected for further analysis. The generated docking models were visualized and analyzed using the CB-Dock3 interface, and the main contact residues involved in ligand–protein interactions were identified for each complex [44]. Docking results were compared against the corresponding reference ligands for each target, allowing the evaluation of the potential binding affinity and interaction profiles of sesamin, sesamolin, (−)-beta-Sitosterol, Campesterol, (+)-gamma-Tocopherol with TNF-α, DJ-1, Aβ42, and caspase-3.

### 2.23. Data Analysis

In this experimental design, neuron–astrocyte co-cultures and 3D model were established at a standardized cellular density as described above in 24 well plates. Cells (i.e., biological and observational units) were randomly assigned to wells using a simple randomization method (sampling without replacement), and wells (i.e., experimental units) were subsequently randomized to treatment conditions following the same procedure. Experimental groups included control (vehicle, 1% ethanol), sesame indicum whole paste extract (SIPE, 10 µg/mL), TGC (100:10:100; TNF-α ng/mL:glutamate µM:cortisol nM), and the combined treatment (SIPE + TGC). All experiments were performed in three (flow cytometry, dot-blot, probe fluorescence and cell-free assays) or six (MTT, NBT and immunofluorescence assays) independent biological replicates (*n* = 3; *n* = 6), and analyses were conducted blind to the experimenter and, when applicable, to the flow cytometry, fluorescence microscopy, ELISA, or biochemical assay analyst [45]. For cell-free assays (RT-QuIC and dot-blot), multiple technical replicates were included within each independent experiment. Data obtained from independent experiments were averaged, and representative images or curves were selected for illustrative purposes. Quantitative results are presented as mean ± standard deviation (SD), with individual data points representing each independent experiment. Prior to statistical analysis, data distribution normality was assessed using the Shapiro–Wilk test, and homogeneity of variances was evaluated using Levene’s test. Statistical significance was determined by one-way analysis of variance (ANOVA) followed by Tukey’s post hoc test for multiple comparisons, using GraphPad Prism software (version 5.0 or later). Differences between groups were considered statistically significant at *p* < 0.05 (*), *p* < 0.01 (**), and *p* < 0.001 (***).

## 3. Results

### 3.1. NPCs and WJ-MSCs Exhibit Neural Stemness and Efficiently Differentiate into Neuronal and Astrocyte-like Lineages

**Figure 1:**
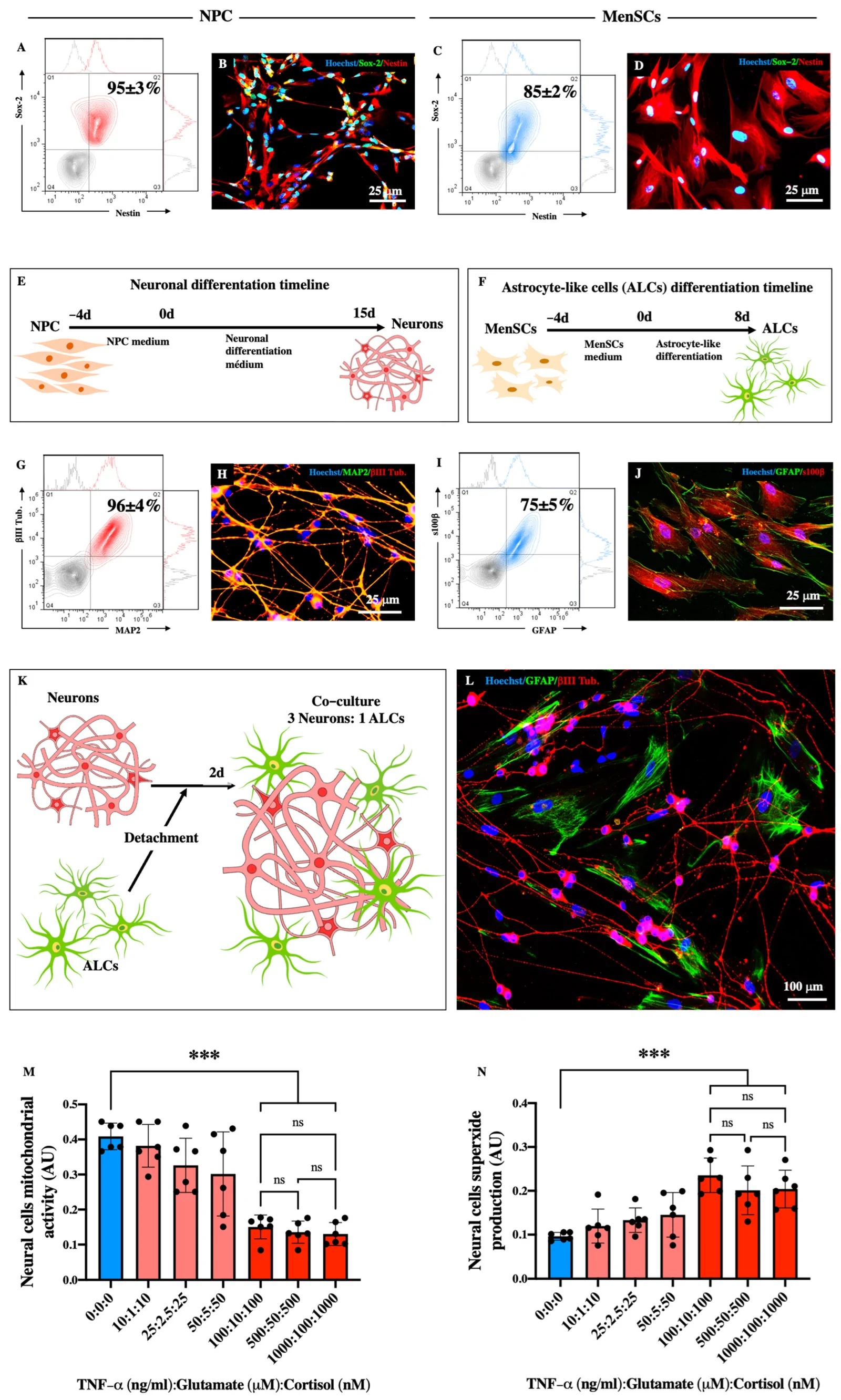
NPCs and WJ-MSCs exhibit neural stemness, differentiate into neuronal and astrocyte-like lineages, and generate a neuron–ALC co-culture model responsive to chronic stress-associated stimuli. (**A**,**B**) Representative flow cytometry and immunofluorescence analyses showing the expression of the neural stemness markers Sox2 and Nestin in iPSC-derived neural precursor cells (NPCs). (**C**,**D**) Flow cytometry and immunofluorescence characterization of Wharton’s jelly-derived mesenchymal stem cells (WJ-MSCs) demonstrating Sox2 and Nestin expression. (**E**,**F**) Schematic representation of neuronal and astrocyte-like cell (ALC) differentiation protocols (arrows indicate the temporal progression of the events). NPCs were differentiated into neurons using Cholinergic-N-Run medium, whereas WJ-MSCs were differentiated into ALCs using astrocyte differentiation medium, as described in Materials and Methods. (**G**,**H**) Representative flow cytometry and immunofluorescence analyses confirming neuronal differentiation through MAP2 and βIII-tubulin expression. (**I**,**J**) Flow cytometry and immunofluorescence analyses validating astrocytic differentiation by GFAP and S100β expression. (**K**) Schematic illustration of the neuron (red cells)–ALC(green cells) co-culture system established by seeding detached ALCs onto pre-differentiated neuronal cultures (arrows indicate the temporal progression of the events). (**L**) Representative immunofluorescence image confirming co-culture establishment by simultaneous detection of βIII-tubulin-positive neurons and GFAP-positive ALCs after 48 h of incubation. (**M**,**N**) Assessment of oxidative stress and mitochondrial dysfunction induced by TGC exposure using MTT and NBT reduction assays, respectively. Image magnification 20×. Scale bars as indicated in each panel. Data are presented as mean ± SD from at least three independent experiments. Statistical significance was determined by one-way ANOVA followed by post hoc analysis (*** *p* < 0.001).

We initially recovered iPSC-derived neural precursor cells (NPCs) and Wharton’s jelly-derived mesenchymal stem cells (WJ-MSCs) following previously established protocols [35,38]. To confirm their neural stem-like phenotype, we evaluated the expression of canonical stemness markers. Both iPSC-derived NPCs (Figure 1A,B) and WJ-MSCs (Figure 1C,D) exhibited robust co-expression of Sox2 and Nestin, as determined by flow cytometry and immunofluorescence analyses, reaching 95 ± 3% and 85 ± 2% positivity, respectively. Next, we assessed their differentiation potential toward neural lineages. NPCs (Figure 1E) and WJ-MSCs (Figure 1F) were cultured in Cholinergic-N-Run and astrocyte differentiation media to generate neurons and astrocyte-like cells (ALCs), respectively. Neuronal differentiation was confirmed by the co-expression of MAP2 and βIII-tubulin (Figure 1G,H), whereas astrocytic differentiation was validated by GFAP and S100β expression (Figure 1I,J), using both flow cytometry and immunofluorescence approaches. Flow cytometry analysis revealed a highly efficient neuronal differentiation, with 96 ± 4% of cells co-expressing MAP2 and βIII-tubulin following NPC induction. Similarly, astrocytic differentiation yielded 75 ± 5% of GFAP/S100β-positive cells. These findings were consistently supported by immunofluorescence imaging, confirming the acquisition of lineage-specific phenotypes. To model neuron–glia interactions, we established a co-culture system by detaching ALCs and seeding them onto pre-differentiated neuronal cultures, as detailed above (Figure 1K). After 48 h of incubation, successful co-culture establishment was confirmed by the simultaneous identification of astrocytic (GFAP) and neuronal (βIII-tubulin) markers through immunofluorescence analysis (Figure 1L).

### 3.2. TNF-α/Glutamate/Cortisol (TGC) Induces Mitochondrial Dysfunction and Superoxide Generation in Neuron–ALC Co-Cultures

To establish an in vitro system reproducing key molecular features associated with chronic stress, we first evaluated the effects of increasing concentrations of a TNF-α/glutamate/cortisol (TGC) cocktail, as detailed in the Section 2. Mitochondrial activity, assessed by MTT assay, revealed that TGC exposure induced a significant decline in cellular metabolic function at concentrations equal to or above 100 ng/mL TNF-α, 10 μM glutamate, and 100 nM cortisol (Figure 1M), indicating the onset of mitochondrial dysfunction. Consistently, the assessment of superoxide anion production using the NBT reduction assay demonstrated a marked increase in mitochondrial-derived reactive oxygen species (ROS) under the same concentration threshold (Figure 1N). These findings collectively indicate that TGC triggers a robust oxidative stress response coupled to impaired mitochondrial activity in neuron–astrocyte-like cell (ALC) co-cultures. Notably, no statistically significant differences were observed among concentrations exceeding this threshold, suggesting a plateau effect in the stress response. Therefore, the condition comprising 100 ng/mL TNF-α, 10 μM glutamate, and 100 nM cortisol was selected as the standard experimental condition for subsequent assays.

### 3.3. Bioactive Compound Enrichment in Sesamum indicum Whole Paste Compared with Seed and Oil Preparations

The Comparative phytochemical profiling revealed that whole sesame paste constitutes the most chemically enriched sesame-derived product among the preparations evaluated, exhibiting a remarkable concentration of multiple classes of bioactive compounds previously associated with antioxidant, anti-inflammatory and neuroprotective activities. Notably, the traditional transformation of sesame seeds into paste resulted in a substantial enrichment of total polyphenols, lignans, phytosterols, tocopherols, and unsaturated fatty acids, generating a phytochemical matrix of considerably greater complexity than either the original seeds or the extracted oil (Table 2 and Table 3). Among all products analyzed, sesame paste exhibited the highest total polyphenol content (96.029 ± 0.073 mg GAE/100 g sample), representing a 24.5% increase compared with sesame seeds (77.138 ± 0.787 mg GAE/100 g sample) and more than a five-fold increase compared with sesame oil (17.874 ± 0.164 mg GAE/100 g sample). This finding is particularly relevant because polyphenols constitute one of the principal antioxidant defense systems in plant-derived foods, contributing to the neutralization of reactive oxygen species, modulation of inflammatory pathways, and preservation of mitochondrial homeostasis. The marked reduction observed in the oil fraction suggests that the extraction process selectively concentrates lipids while simultaneously removing a substantial proportion of polar antioxidant metabolites.

Comprehensive GC–MS profiling of the ethanolic extracts prepared from sesame seed, whole sesame paste, and sesame oil identified ~38 metabolites and several unidentified chromatographic peaks (Table 3; Supplementary Table S1). Interestingly, this analysis further demonstrated a profound enrichment of the characteristic sesame lignans in sesame paste. (+)-Sesamin, the major bioactive lignan identified, reached 24.70 ± 0.12% relative area, corresponding to a 2.15-fold increase compared with seeds (11.48 ± 0.12%) and exceeding the abundance detected in sesame oil (21.73 ± 0.20%). Likewise, sesamolin reached 10.14 ± 0.05%, representing approximately a three-fold increase compared with seeds (3.45 ± 0.04%) and a 33% increase compared with oil (7.62 ± 0.07%). These observations are of biological significance because sesamin and sesamolin have been extensively associated with activation of endogenous antioxidant responses, suppression of NF-κB-dependent inflammatory signaling, modulation of lipid metabolism, protection against mitochondrial dysfunction, and attenuation of amyloidogenic and neurodegenerative processes. The simultaneous enrichment of both lignans strongly suggests that traditional paste production preserves the phytochemical architecture of sesame while concentrating compounds with recognized therapeutic potential.

A similar trend was observed for phytosterols. Sesame paste contained the highest levels of β-sitosterol (13.75 ± 0.07%), campesterol (3.87 ± 0.02%), stigmasterol (2.31 ± 0.01%), and isofucosterol (2.63 ± 0.01%), consistently exceeding the concentrations detected in seeds and slightly surpassing those found in sesame oil. β-Sitosterol alone was enriched by approximately 166% relative to seeds. This finding is noteworthy because phytosterols have been linked to anti-inflammatory effects, attenuation of oxidative stress, and modulation of microglial activation, mechanisms increasingly recognized as relevant in age-related neurodegenerative disorders. Furthermore, sesame paste exhibited the highest abundance of γ-tocopherol (3.84 ± 0.02%), an important lipophilic antioxidant capable of scavenging reactive nitrogen species and protecting membrane lipids from oxidative damage. Although sesame oil retained a favorable lipid composition characterized by elevated levels of linoleic acid (42.04 ± 0.39%) and oleic acid (35.43 ± 0.33%), its phytochemical diversity was substantially lower than that of sesame paste. In contrast, sesame paste combined a highly favorable unsaturated fatty acid profile (38.87 ± 0.19% linoleic acid and 35.94 ± 0.18% oleic acid) with the highest concentrations of antioxidant and neuroprotective secondary metabolites. This unique combination may provide synergistic biological effects, as unsaturated fatty acids contribute to membrane integrity and neuronal function, while lignans, polyphenols, tocopherols, and phytosterols collectively enhance antioxidant defenses and regulate inflammatory signaling pathways.

### 3.4. Sesamum indicum Whole Paste Extract (SIPE) Confers Maximal Protective Effect Against TGC-Induced Mitochondrial Dysfunction and Superoxide Generation in Neuron–ALC Co-Cultures

**Figure 2:**
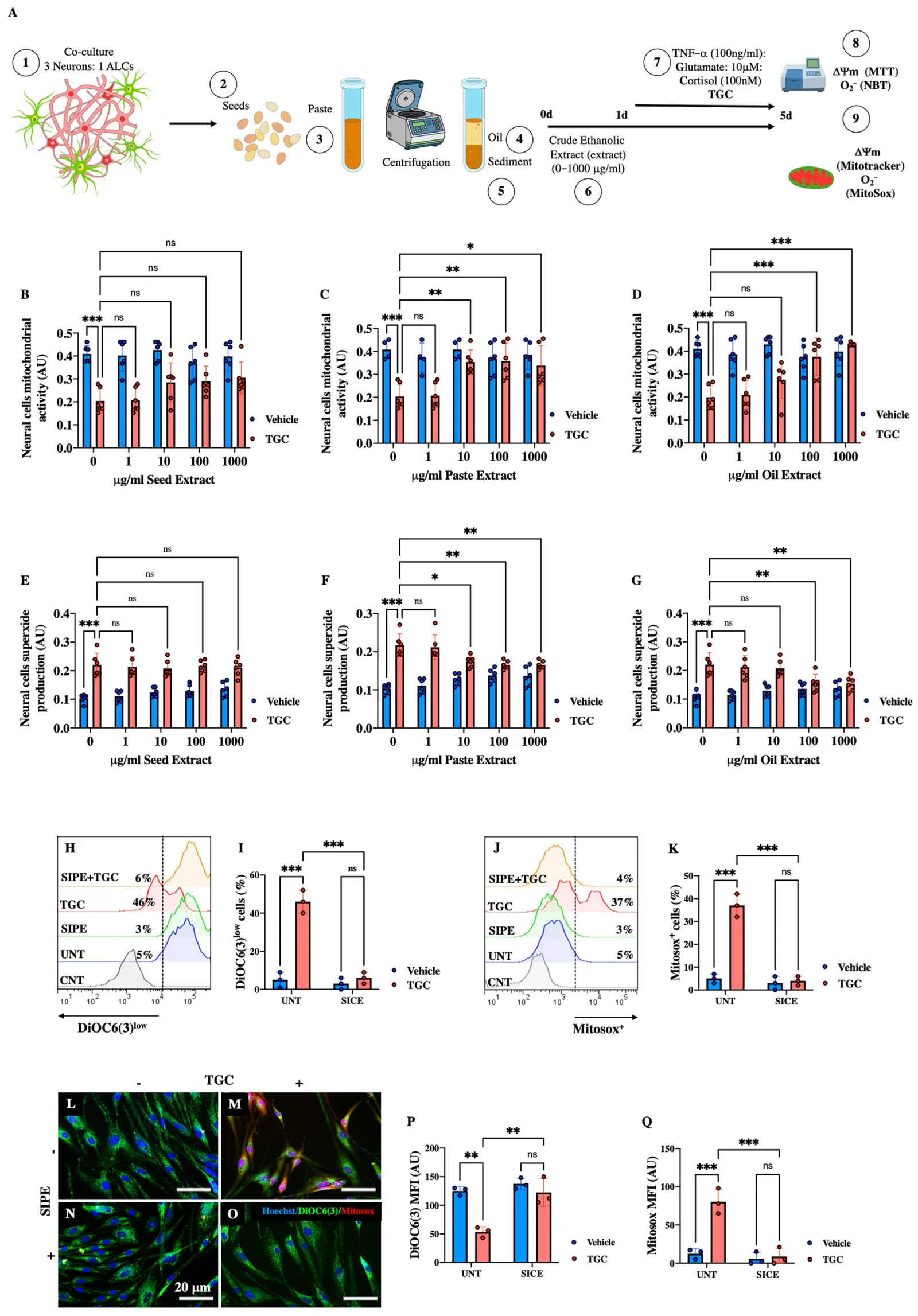
*Sesamum indicum* whole paste extract (SIPE) confers maximal protection against TGC-induced mitochondrial dysfunction and oxidative stress in neuron–ALC co-cultures. (**A**) Experimental design illustrating the preparation of *Sesamum indicum* derivatives from unprocessed seeds, whole paste, and oil, followed by exposure of neuron–ALC co-cultures to TNF-α/glutamate/cortisol (TGC) in the presence or absence of increasing extract concentrations (0, 1, 10, 100, and 1000 μg/mL). Mitochondrial activity and superoxide anion production were assessed using MTT and NBT reduction assays, respectively. (**B**–**D**) MTT analyses showing the effects of seed, whole paste, and oil extracts on mitochondrial metabolic activity in TGC-exposed neuron–ALC co-cultures. (**E**–**G**) NBT reduction assays evaluating superoxide anion production following treatment with seed, whole paste, and oil extracts under TGC exposure. (**H**,**I**) Representative flow cytometry histograms and quantitative analyses of ΔΨm assessed using the DiOC6(3) probe. (**J**,**K**) Representative flow cytometry histograms and quantitative analyses of mitochondrial superoxide generation determined using the MitoSOX probe. (**L**–**O**) Representative fluorescence microscopy images showing nuclei (blue), DiOC6(3) staining (green) for mitochondrial membrane potential, and MitoSOX staining (red) for mitochondrial superoxide production in neuron–ALC co-cultures. (**P**) Quantification of DiOC6(3) mean fluorescence intensity (MFI). (**Q**) Quantification of MitoSOX mean fluorescence intensity (MFI). Data are presented as mean ± SD from at least three independent experiments. Statistical significance was determined by one-way ANOVA followed by post hoc analysis (* *p* < 0.05, ** *p* < 0.01, *** *p* < 0.001). Image magnification 20×. Scale bars as indicated in each panel.

To assess the neuroprotective potential of *Sesamum indicum* derivatives against TGC-induced toxicity, we prepared extracts from unprocessed seeds, whole paste and oil across a concentration range (0, 1, 10, 100, and 1000 μg/mL). These extracts were tested in the absence or presence of TGC at the previously established stress-inducing condition. Then, mitochondrial activity and superoxide anion production were evaluated using MTT and NBT reduction assays, respectively (Figure 2A). MTT analysis revealed that unprocessed seed extracts failed to prevent the TGC-induced impairment of mitochondrial activity at all tested concentrations (Figure 2B). In contrast, treatment with whole paste (Figure 2C) and oil (Figure 2D) extracts significantly preserved mitochondrial function. Notably, the whole paste extract exhibited the highest efficacy, conferring protection from as low as 10 μg/mL, whereas oil extract required higher concentrations (≥100 μg/mL) to achieve comparable effects. Consistent with these findings, NBT reduction assays demonstrated that seed extracts did not attenuate TGC-induced superoxide anion production (Figure 2E). However, exposure to whole paste (Figure 2F) and oil (Figure 2G) extracts significantly reduced mitochondrial ROS generation. Again, the whole paste extract showed superior potency, suppressing superoxide production from 10 μg/mL, while the oil extract was effective only at higher concentrations (≥100 μg/mL). Taken together, these results indicate that *Sesamum indicum* derivatives—particularly the whole paste formulation—effectively counteract TGC-induced mitochondrial dysfunction and oxidative stress in neuron–ALC co-cultures. Based on its superior efficacy at low concentrations, the whole paste extract at 10 μg/mL was selected for subsequent experimental procedures.

To further validate the protective effect of the whole paste extract from *Sesamum indicum* at 10 μg/mL, we assessed mitochondrial membrane potential (ΔΨm) using the DiOC6(3) probe, as well as mitochondrial superoxide production using the MitoSOX probe, in neuron–ALC co-cultures exposed to TGC in the presence or absence of the extract. Analyses were performed by flow cytometry and fluorescence microscopy. Flow cytometry analysis revealed that exposure to TGC induced a marked loss of mitochondrial membrane potential, with a reduction of approximately 46% compared with untreated control cells (~5%) or cells treated with the *Sesamum indicum* whole paste extract alone (SIPE; ~3%) (Figure 2H,I). Notably, pre-treatment with SIPE effectively preserved mitochondrial integrity, maintaining ΔΨm at levels comparable to untreated controls (~5%), thereby preventing TGC-induced depolarization. Consistently, TGC exposure significantly increased mitochondrial superoxide production (~37%) relative to untreated (~5%) or SIPE-treated cells (~3%) (Figure 2J,K). Importantly, pre-treatment with SIPE abolished this effect, maintaining superoxide levels near baseline (~4%), indicating a strong attenuation of mitochondrial oxidative stress. These findings were further corroborated by fluorescence microscopy analyses (Figure 2L–Q), which confirmed the preservation of mitochondrial function and the suppression of superoxide generation in SIPE-treated co-cultures.

### 3.5. Sesamum indicum Whole Paste Extract (SIPE) Prevents TGC-Induced Neuronal Loss and Attenuates Gliosis in Neuron–ALC Co-Cultures

**Figure 3:**
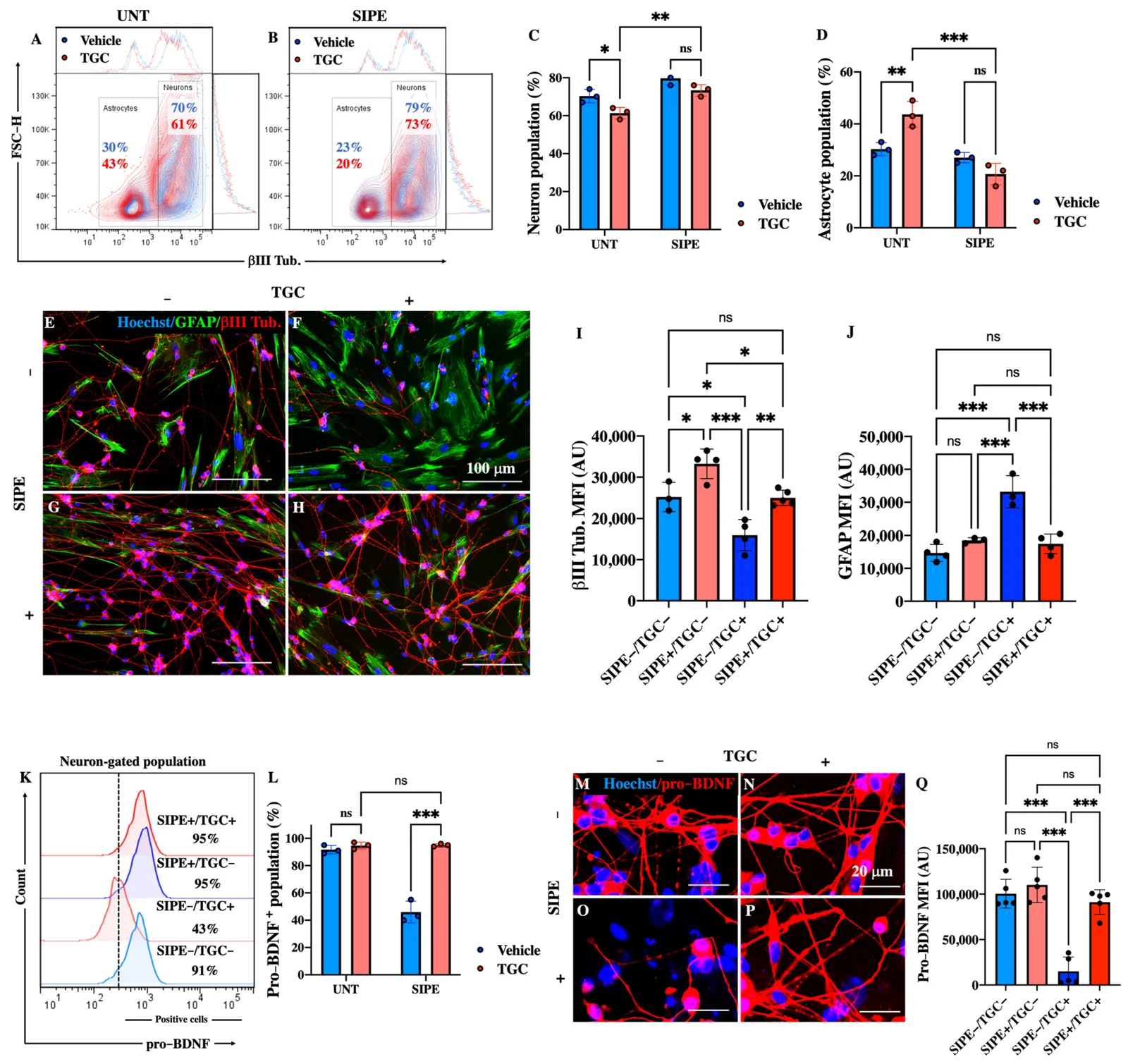
*Sesamum indicum* whole paste extract (SIPE) prevents TGC-induced neuronal loss, attenuates gliosis, and preserves neurotrophic signaling in neuron–ALC co-cultures. (**A**,**B**) Representative flow cytometry contour plots illustrating the proportion of βIII-tubulin-positive neurons and astrocyte-like cells (ALCs) in neuron–ALC co-cultures exposed to TNF-α/glutamate/cortisol (TGC) in the presence or absence of SIPE (10 μg/mL). (**C**,**D**) Quantitative analysis of βIII-tubulin-positive neuronal populations determined by flow cytometry. (**E**–**H**) Representative fluorescence microscopy images illustrating neuron–ALC co-cultures under vehicle, SIPE, TGC, and SIPE + TGC conditions, showing nuclear staining (blue), βIII-tubulin-positive neuronal networks (red) and GFAP-positive ALCs (green). (**I**) Quantification of βIII-tubulin mean fluorescence intensity (MFI). (**J**) Quantification of GFAP mean fluorescence intensity (MFI). (**K**) Representative flow cytometry histograms showing pro-BDNF expression in neuron–ALC co-cultures. (**L**) Flow cytometry quantification of pro-BDNF-positive cells under the indicated experimental conditions. (**M**–**P**) Representative fluorescence microscopy images showing pro-BDNF immunoreactivity (red) and nuclear staining (blue) in neuron–ALC co-cultures exposed to vehicle, SIPE, TGC, or SIPE + TGC. (**Q**) Quantification of pro-BDNF mean fluorescence intensity (MFI). Data are presented as mean ± SD from at least three independent experiments. Statistical significance was determined by one-way ANOVA followed by post hoc analysis (* *p* < 0.05, ** *p* < 0.01, *** *p* < 0.001). Image magnification 20× (**E**–**H**) and 100× (**M**–**P**). Scale bars as indicated in each panel.

To further evaluate the neuroprotective potential of *Sesamum indicum* whole paste extract (SIPE), we assessed changes in neuronal and astrocyte-like cell (ALC) populations within neuron–ALC co-cultures following TGC exposure. Neuronal integrity was determined by quantifying βIII-tubulin-positive cells, using both flow cytometry and fluorescence microscopy. Flow cytometry analysis revealed that TGC exposure induced a significant reduction in the neuronal population, as evidenced by a decrease of approximately 18% in βIII-tubulin-positive cells compared with vehicle-treated controls (~70%) and SIPE-treated cells (~79%) (Figure 3A–C). Notably, pre-treatment with SIPE effectively preserved neuronal identity, maintaining βIII-tubulin reactivity at levels comparable to untreated controls (~73%), thereby preventing TGC-induced neuronal loss (Figure 3B,C). Concomitantly, TGC exposure led to a marked increase in the proportion of ALCs (up to ~43%), compared with untreated (~30%) and SIPE-treated conditions (~23%) (Figure 3A–D), suggesting a reactive gliosis-like response. Importantly, SIPE pre-treatment attenuated this effect, maintaining ALC levels close to baseline (~23%), indicative of a protective modulation of neuron–glia dynamics. These findings were consistently corroborated by fluorescence microscopy analyses (Figure 3E–J), which confirmed the preservation of neuronal networks and the prevention of aberrant glial expansion in SIPE-treated co-cultures.

To further determine whether SIPE-mediated neuroprotection extends beyond preservation of cellular proportions and involves enhanced neuronal survival, we evaluated pro-BDNF expression as a surrogate marker of neuronal viability and neurotrophic signaling. Flow cytometry analysis demonstrated that TGC exposure significantly reduced the proportion of pro-BDNF-positive cells (~48% decrease) compared with vehicle-treated (~91%) and SIPE-treated cells (~95%) (Figure 3K). Strikingly, SIPE pre-treatment fully prevented this decline, maintaining pro-BDNF levels comparable to baseline (~95%), thereby supporting a role for SIPE in sustaining neurotrophic signaling under stress conditions. These results were further validated by fluorescence microscopy (Figure 3M–Q), confirming the preservation of pro-BDNF-positive neuronal populations in SIPE-treated co-cultures.

### 3.6. Sesamum indicum Whole Paste Extract (SIPE) Prevents TGC-Induced Glial Reactivity and Preserves ALC Functional Phenotype in Neuron–ALC Co-Cultures

**Figure 4:**
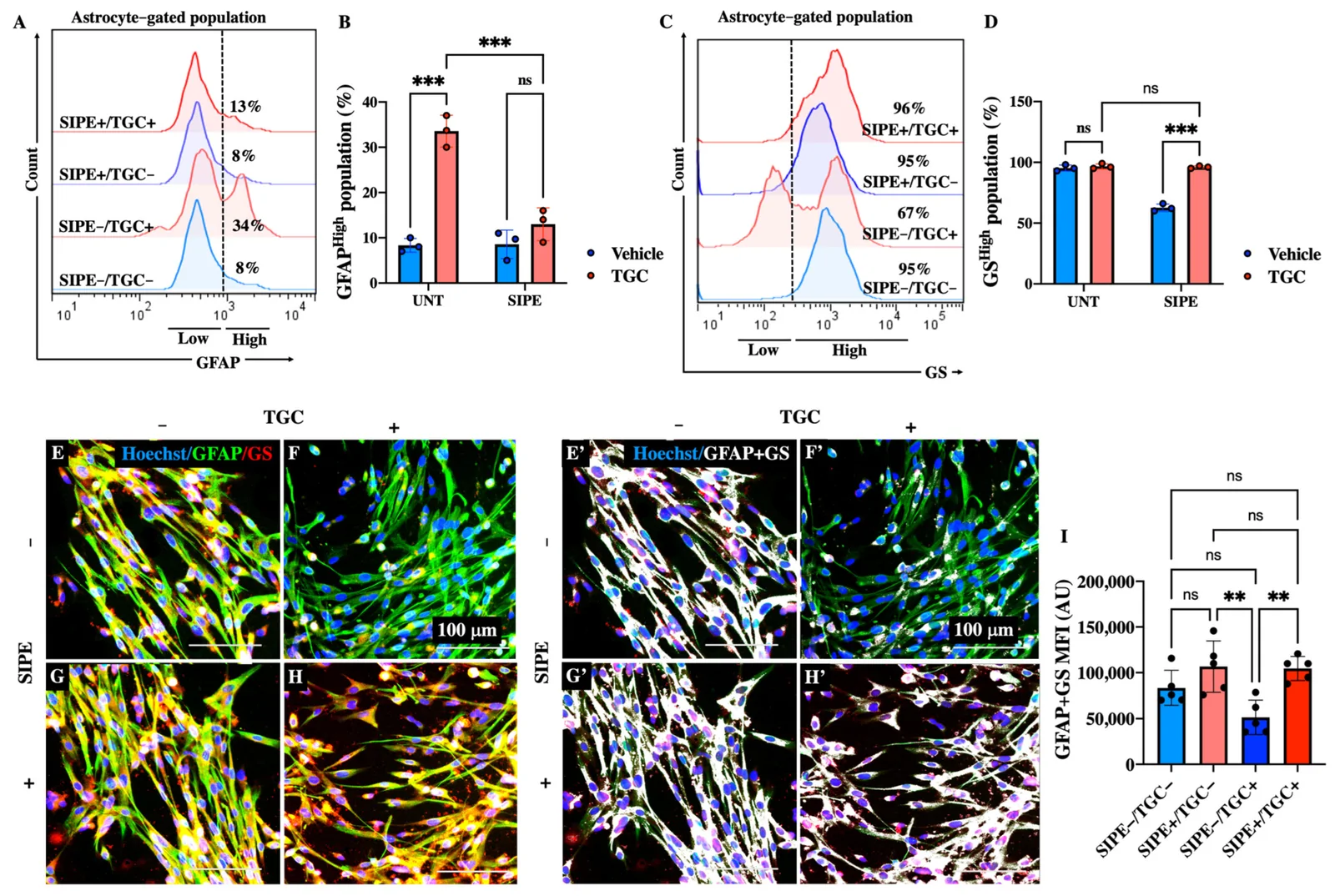
*Sesamum indicum* whole paste extract (SIPE) attenuates TGC-induced astrocytic reactivity and preserves glutamine synthetase expression in neuron–ALC co-cultures. (**A**) Representative flow cytometry histograms and (**B**) quantitative analyses illustrating the proportion of GFAP^high^ astrocyte-like cells (ALCs) in neuron–ALC co-cultures exposed to TNF-α/glutamate/cortisol (TGC) in the presence or absence of SIPE (10 μg/mL). (**C**) Representative flow cytometry histograms and (**D**) quantitative analyses showing glutamine synthetase (GS) expression and the proportion of GS^high^ ALCs under the indicated experimental conditions. (**E**–**H**) Representative fluorescence microscopy images showing GFAP (green) and GS (red) immunoreactivity and nuclear staining (blue) in neuron–ALC co-cultures exposed to vehicle, SIPE, TGC, or SIPE + TGC conditions. (**E′**–**H′**) Representative fluorescence microscopy images showing GFAP+GS simultaneous immunoreactivity (white) under the indicated experimental conditions. (**I**) Quantification of GFAP+GS mean fluorescence intensity (MFI) obtained from fluorescence microscopy analyses. Data are presented as mean ± SD from at least three independent experiments. Statistical significance was determined by one-way ANOVA followed by post hoc analysis (** *p* < 0.01, *** *p* < 0.001). Image magnification 20×. Scale bars as indicated in each panel.

Given that TGC exposure promoted a marked increase in the proportion of astrocyte-like cells (ALCs) (Figure 3A–J) and considering that glutamate-driven excitotoxicity has been widely associated with the induction of reactive astrocyte phenotypes under chronic stress conditions [46,47], we next evaluated ALC reactivity by assessing GFAP, S100β, Vimentin and glutamine synthetase (GS) expression using flow cytometry and fluorescence microscopy in neuron–ALC co-cultures following SIPE and/or TGC exposure. Flow cytometry analysis revealed that TGC exposure induced a significant shift toward a reactive astrocytic phenotype, as evidenced by an increase of approximately 34% in GFAP^high^ cells compared with vehicle-treated (~8%) and SIPE-treated conditions (~8%) (Figure 4A). Notably, pre-treatment with *Sesamum indicum* whole paste extract (SIPE) effectively attenuated this response, maintaining GFAP^high^ levels close to baseline (~13%), thereby preventing TGC-induced astrocytic reactivity. Similar results were obtained by determining S100β and Vimentin reactivity in Neuron–ALC Co-Cultures following SIPE and/or TGC exposure (Supplementary Figure S2).

Concomitantly, TGC exposure significantly impaired ALC functional status, as indicated by a reduction of approximately 28% in GS^high^ cells relative to vehicle (~95%) and SIPE-treated cells (~95%) (Figure 4B). Importantly, SIPE pre-treatment preserved GS expression, maintaining GS^high^ levels comparable to untreated controls (~96%), suggesting the maintenance of astrocytic metabolic and glutamate-buffering capacity. These results were further validated by fluorescence microscopy (Figure 4E–H,E′–H′,I), which confirmed the preservation of both structural (GFAP) and functional (GS) astrocytic markers in SIPE-treated co-cultures.

### 3.7. Sesamum indicum Whole Paste Extract (SIPE) Attenuates TGC-Induced NF-κB Activation and Pro-Inflammatory Cytokine Release in Neuron–ALC Co-Cultures

**Figure 5:**
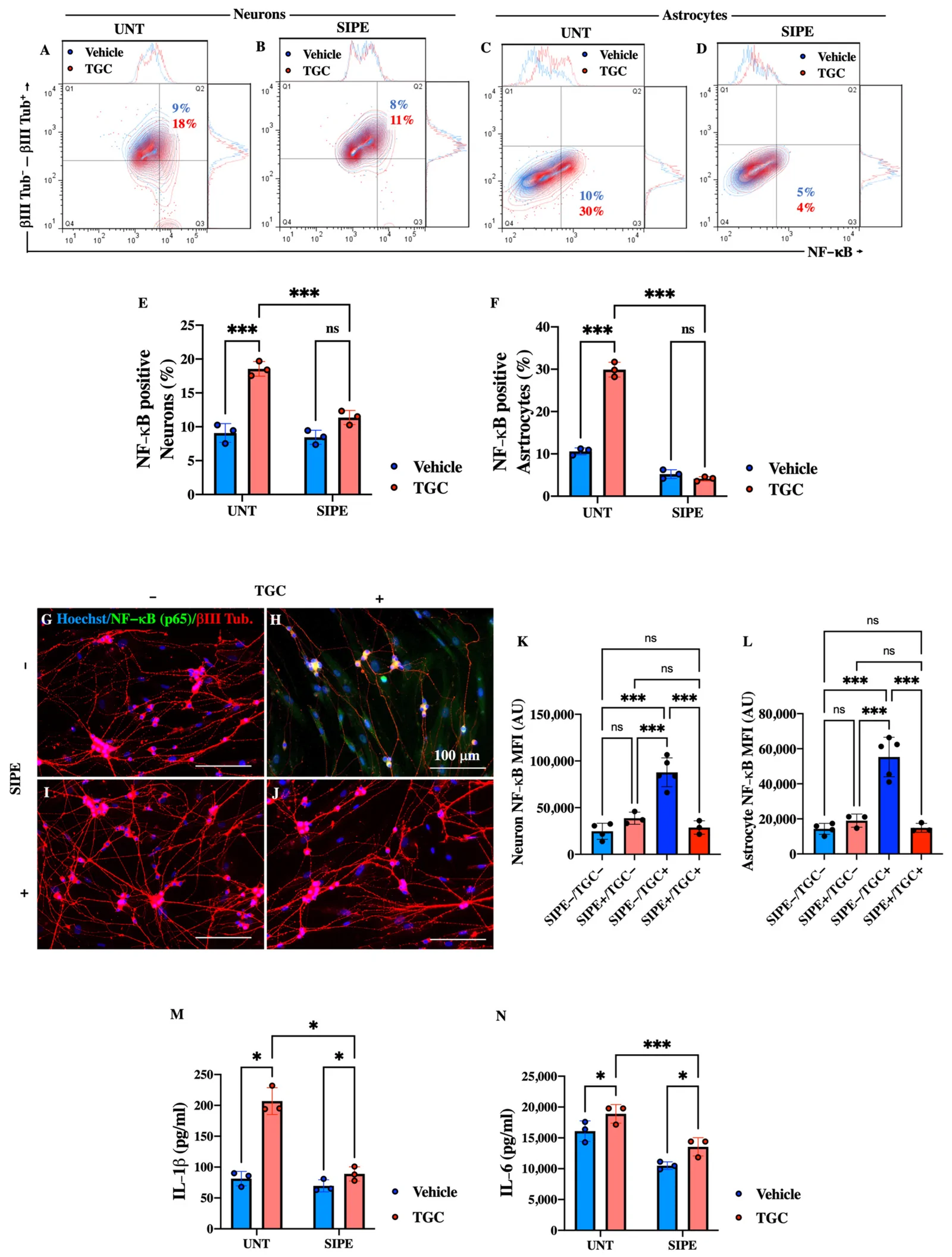
*Sesamum indicum* whole paste extract (SIPE) attenuates TGC-induced NF-κB activation and pro-inflammatory cytokine release in neuron–ALC co-cultures. (**A**,**B**) Representative flow cytometry contour plots illustrating neuronal NF-κB activation, defined as βIII-tubulin/NF-κB double-positive cells, in neuron–ALC co-cultures exposed to TNF-α/glutamate/cortisol (TGC) in the presence or absence of SIPE (10 μg/mL). (**C**,**D**) Representative flow cytometry contour plots showing astrocytic NF-κB activation, defined as βIII-tubulin-negative/NF-κB-positive cells, under the indicated experimental conditions. (**E**,**F**) Quantitative analyses of neuronal and astrocytic NF-κB activation determined by flow cytometry. (**G**–**J**) Representative fluorescence microscopy images illustrating NF-κB immunoreactivity (green), βIII-tubulin-positive neurons (red), and nuclear staining (blue) in neuron–ALC co-cultures exposed to vehicle, SIPE, TGC, or SIPE + TGC conditions. (**K**) Quantification of βIII-tubulin-positive/NF-κB-positive cells’ mean fluorescence intensity (MFI) obtained from fluorescence microscopy analyses. (**L**) Quantification of βIII-tubulin-negative/NF-κB-positive cells mean fluorescence intensity (MFI) obtained from fluorescence microscopy analyses. (**M**,**N**) Quantification of IL-1β and IL-6 release in neuron–ALC co-culture supernatants under the indicated experimental conditions. Data are presented as mean ± SD from at least three independent experiments. Statistical significance was determined by one-way ANOVA followed by post hoc analysis (* *p* < 0.05, *** *p* < 0.001). Image magnification 20×. Scale bars as indicated in each panel.

To further characterize the neuroprotective and anti-inflammatory effects of *Sesamum indicum* whole paste extract (SIPE), we evaluated nuclear factor kappa B (NF-κB, p65) activation in both neuronal and astrocyte-like cell (ALC) populations within neuron–ALC co-cultures following TGC exposure. Neuronal NF-κB activation was quantified as βIII-tubulin/NF-κB double-positive cells, whereas astrocytic NF-κB activation was defined as βIII-tubulin-negative/NF-κB-positive cells. Analyses were performed using flow cytometry and fluorescence microscopy. Flow cytometry analysis demonstrated that TGC exposure significantly increased neuronal NF-κB activation, as evidenced by an ~18% rise in βIII-tubulin/NF-κB double-positive cells compared with vehicle-treated (~9%) and SIPE-treated cells (~8%) (Figure 5A,B,E). Notably, pre-treatment with SIPE markedly attenuated this response, maintaining neuronal NF-κB activation at levels comparable to untreated controls (~11%), thereby preventing TGC-induced pro-inflammatory signaling in neurons. Similarly, TGC exposure significantly enhanced NF-κB activation in ALCs, with an approximate 30% increase in βIII-tubulin-negative/NF-κB-positive cells compared with vehicle (~10%) and SIPE-treated conditions (~5%) (Figure 5C,D,F). Importantly, SIPE pre-treatment effectively suppressed this astrocytic response, maintaining NF-κB activation near baseline (~4%), indicative of a robust inhibition of glial pro-inflammatory activation. These findings were consistently corroborated by fluorescence microscopy (Figure 5G–L), which confirmed the attenuation of NF-κB nuclear reactivity in both neuronal and astrocytic compartments. Collectively, these results indicate that SIPE exerts a dual anti-inflammatory effect by modulating NF-κB signaling and preventing the establishment of a TGC-induced pro-inflammatory phenotype in neuron–ALC co-cultures.

To further validate the anti-inflammatory effects of SIPE, we quantified the release of key pro-inflammatory cytokines, interleukin-1β (IL-1β) and interleukin-6 (IL-6), in neuron–ALC co-cultures following TGC exposure. Flow cytometry analysis revealed that TGC significantly increased IL-1β release (207 ± 21 pg/mL) compared with vehicle-treated (81 ± 11 pg/mL) and SIPE-treated cells (69 ± 9 pg/mL) (Figure 5M). Notably, SIPE pre-treatment markedly attenuated this response, maintaining IL-1β levels close to baseline (89 ± 11 pg/mL), indicating effective suppression of TGC-induced inflammatory signaling. Consistently, TGC exposure induced a significant increase in IL-6 release (18,900 ± 1514 pg/mL) relative to vehicle-treated (16,084 ± 1683 pg/mL) and SIPE-treated conditions (10,496 ± 615 pg/mL) (Figure 5N). Importantly, SIPE pre-treatment significantly reduced IL-6 levels (13,575 ± 1408 pg/mL), further supporting its role in attenuating inflammatory responses.

### 3.8. Sesamum indicum Whole Paste Extract (SIPE) Attenuates TGC-Induced Intra- and Extracellular Aβ42 Accumulation and Aggregation in Neuron–ALC Co-Cultures

**Figure 6:**
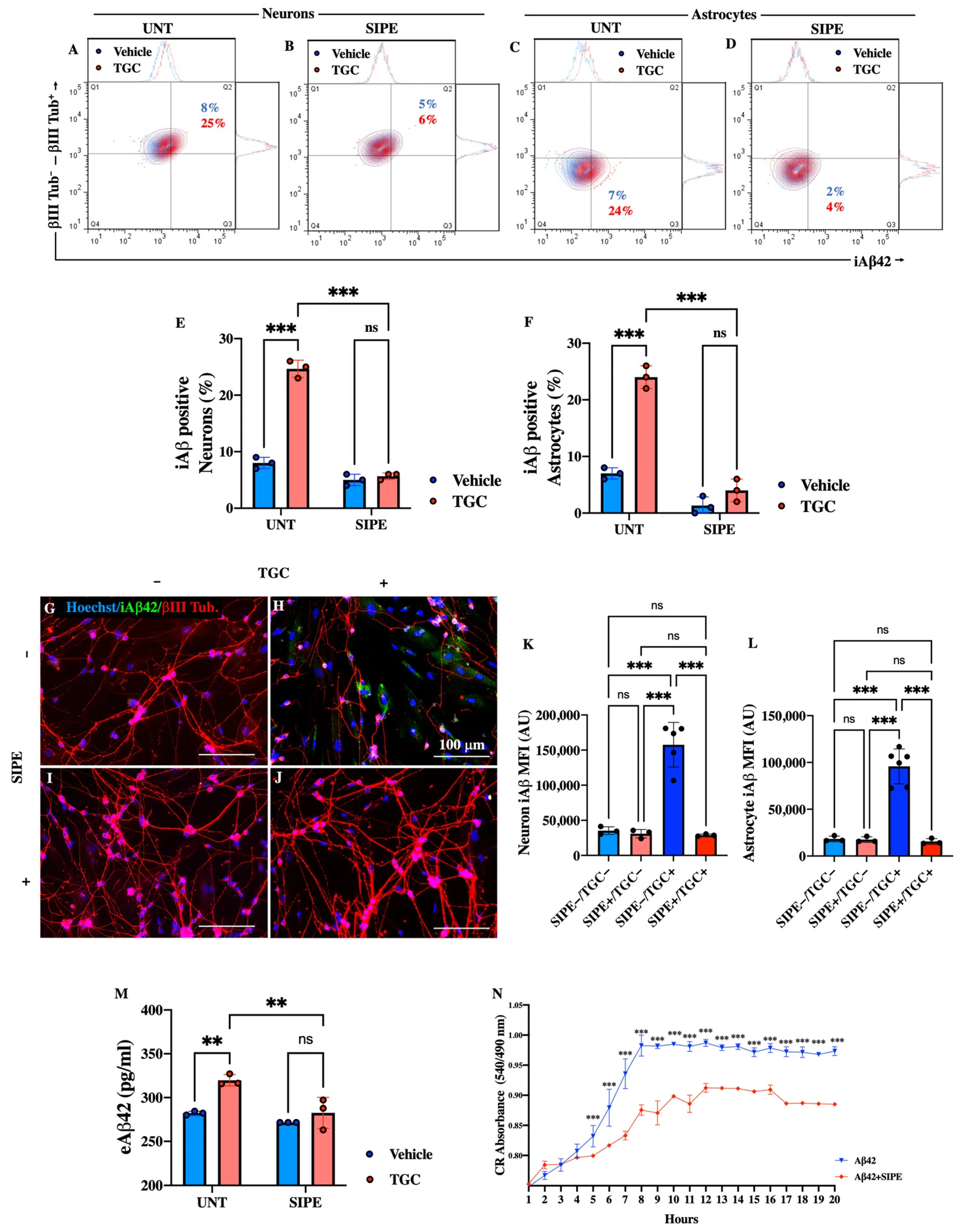
*Sesamum indicum* whole paste extract (SIPE) attenuates TGC-induced intra- and extracellular Aβ42 accumulation and interferes with Aβ42 aggregation kinetics in neuron–ALC co-cultures. (**A**,**B**) Representative flow cytometry contour plots illustrating neuronal intracellular Aβ42 (iAβ42) accumulation, defined as βIII-tubulin/iAβ42 double-positive cells, in neuron–ALC co-cultures exposed to TNF-α/glutamate/cortisol (TGC) in the presence or absence of SIPE (10 μg/mL). (**C**,**D**) Representative flow cytometry contour plots showing astrocytic iAβ42 accumulation, defined as βIII-tubulin-negative/iAβ42-positive cells, under the indicated experimental conditions. (**E**,**F**) Quantitative analyses of neuronal and astrocytic iAβ42 accumulation determined by flow cytometry. (**G**–**J**) Representative fluorescence microscopy images illustrating intracellular Aβ42 immunoreactivity (green), βIII-tubulin-positive neurons (red), and nuclear staining (blue) in neuron–ALC co-cultures exposed to vehicle, SIPE, TGC, or SIPE + TGC conditions. (**K**) Quantification of βIII-tubulin-positive/Aβ42-positive cells’ mean fluorescence intensity (MFI) obtained from fluorescence microscopy analyses. (**L**) Quantification of βIII-tubulin-negative/Aβ42-positive cells’ mean fluorescence intensity (MFI) obtained from fluorescence microscopy analyses. (**M**) Quantification of extracellular Aβ42 (eAβ42) levels in neuron–ALC co-culture supernatants under the indicated experimental conditions. (**N**) RT-QuIC kinetic analysis of synthetic Aβ42 aggregation monitored by Congo Red relative absorbance in the presence or absence of SIPE, illustrating the effects of SIPE on amyloid fibril formation and aggregation dynamics. Data are presented as mean ± SD from at least three independent experiments. Statistical significance was determined by one-way ANOVA followed by post hoc analysis (** *p* < 0.01, *** *p* < 0.001). Image magnification 20×. Scale bars as indicated in each panel.

To further characterize the anti-amyloidogenic effects of *Sesamum indicum* whole paste extract (SIPE), we evaluated intracellular Aβ42 (iAβ42) accumulation in both neuronal and astrocyte-like cell (ALC) populations within neuron–ALC co-cultures following TGC exposure. Neuronal iAβ42 was quantified as βIII-tubulin/iAβ42 double-positive cells, whereas astrocytic iAβ42 accumulation was defined as βIII-tubulin-negative/iAβ42-positive cells. Analyses were performed using flow cytometry and fluorescence microscopy. Flow cytometry analysis demonstrated that TGC exposure significantly increased neuronal iAβ42 accumulation, as evidenced by a ~25% rise in βIII-tubulin/iAβ42 double-positive cells compared with vehicle-treated (~8%) and SIPE-treated cells (~5%) (Figure 6A,B,E). Notably, pre-treatment with SIPE markedly attenuated this response, maintaining neuronal iAβ42 levels near baseline (~6%), thereby preventing TGC-induced amyloidogenic accumulation in neurons. Similarly, TGC exposure significantly enhanced iAβ42 accumulation in ALCs, with an approximate 24% increase in βIII-tubulin-negative/iAβ42-positive cells compared with vehicle (~7%) and SIPE-treated conditions (~2%) (Figure 6C,D,F). Importantly, SIPE pre-treatment effectively suppressed this astrocytic accumulation, maintaining iAβ42 levels close to control values (~4%), indicative of a robust inhibition of amyloidogenic processes across cell types. These findings were consistently corroborated by fluorescence microscopy (Figure 6G–L), which confirmed the reduction in intracellular Aβ42 immunoreactivity in both neuronal and astrocytic compartments.

To further validate the anti-amyloidogenic effects of SIPE, we quantified extracellular Aβ42 (eAβ42) levels in neuron–ALC co-cultures following TGC exposure. Flow cytometry analysis revealed that TGC significantly increased eAβ42 release (320 ± 6 pg/mL) compared with vehicle-treated (282 ± 2 pg/mL) and SIPE-treated cells (271 ± 1 pg/mL) (Figure 6M). Notably, SIPE pre-treatment markedly attenuated this increase, maintaining eAβ42 levels close to baseline (~281 ± 16 pg/mL), indicating effective control of TGC-induced extracellular amyloid burden. To determine whether SIPE directly modulates Aβ42 aggregation kinetics, we performed RT-QuIC analysis using synthetic Aβ42. Kinetic analysis revealed the characteristic three-phase aggregation profile: an initial lag phase (0–4 h; 0–20% Congo Red relative absorbance), corresponding to sub-detectable seed formation; an exponential growth phase (5–8 h; 20–90% CR), reflecting rapid fibril elongation and secondary nucleation; and a plateau phase (>9 h; 90–100% CR), indicative of substrate depletion and fibril stabilization (Figure 6N). Importantly, SIPE treatment significantly reduced Aβ42 fibrillar growth, predominantly during the exponential and plateau phases, resulting in an approximate 40% decrease in Congo Red relative absorbance. These findings indicate that SIPE interferes with both fibril elongation and aggregation stability, supporting a direct anti-amyloidogenic effect.

### 3.9. Sesamum indicum Whole Paste Extract (SIPE) Prevents TGC-Induced DJ-1 Oxidation in Neuron–ALC Co-Cultures

**Figure 7:**
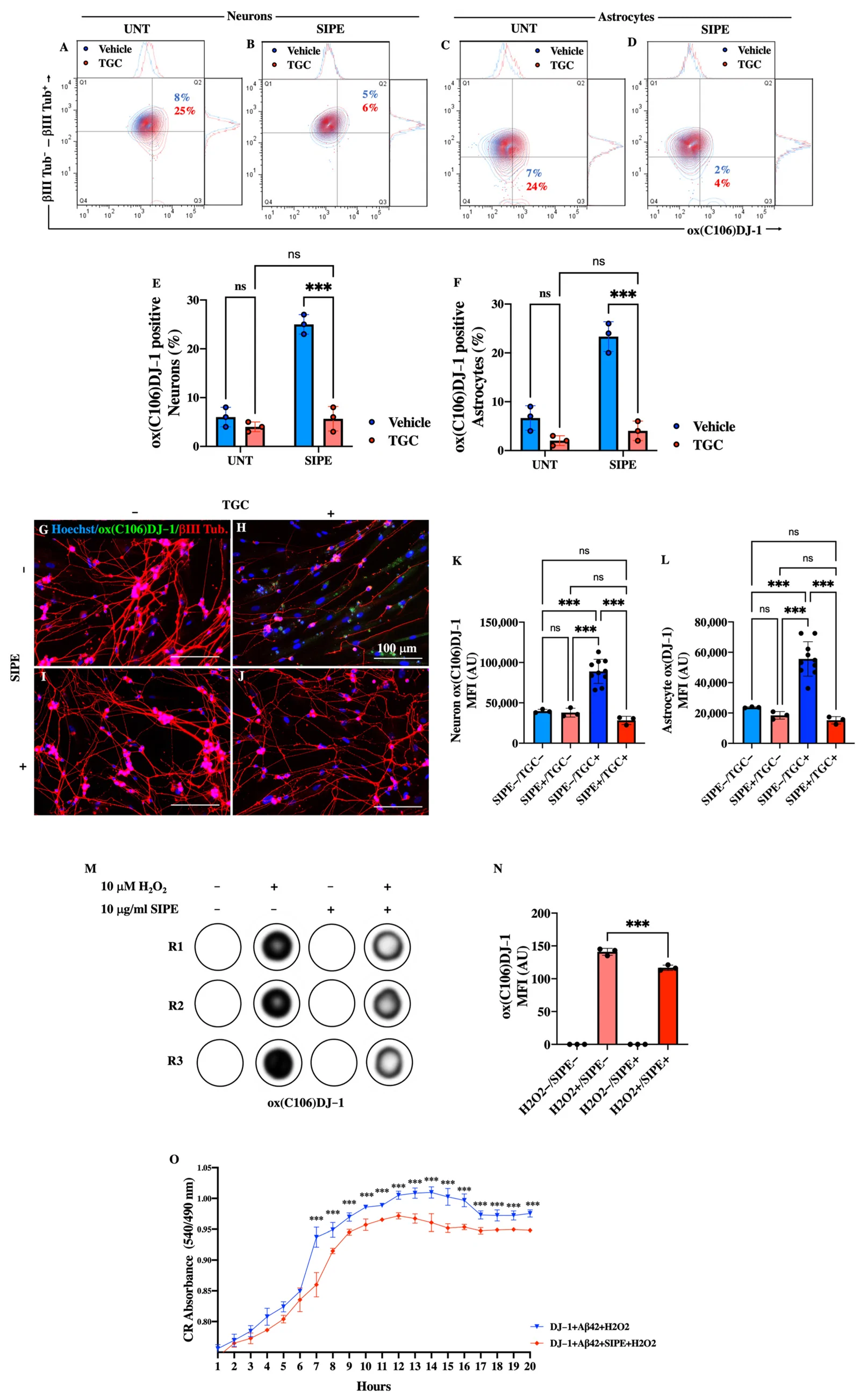
*Sesamum indicum* whole paste extract (SIPE) prevents TGC-induced DJ-1 oxidation and attenuates oxidation-dependent Aβ42 aggregation in neuron–ALC co-cultures. (**A**,**B**) Representative flow cytometry contour plots illustrating neuronal DJ-1 oxidation, defined as βIII-tubulin/ox(C106)DJ-1 double-positive cells, in neuron–ALC co-cultures exposed to TNF-α/glutamate/cortisol (TGC) in the presence or absence of SIPE (10 μg/mL). (**C**,**D**) Representative flow cytometry contour plots showing astrocytic DJ-1 oxidation, defined as βIII-tubulin-negative/ox(C106)DJ-1-positive cells, under the indicated experimental conditions. (**E**,**F**) Quantitative analyses of neuronal and astrocytic ox(C106)DJ-1 accumulation determined by flow cytometry. (**G**–**J**) Representative fluorescence microscopy images illustrating ox(C106)DJ-1 immunoreactivity (green), βIII-tubulin-positive neurons (red), and nuclear staining (blue) in neuron–ALC co-cultures exposed to vehicle, SIPE, TGC, or SIPE + TGC conditions. (**K**) Quantification of βIII-tubulin-positive/ox(C106)DJ-1-positive cells mean fluorescence intensity (MFI) obtained from fluorescence microscopy analyses. (**L**) Quantification of βIII-tubulin-negative/ox(C106)DJ-1-positive cells mean fluorescence intensity (MFI) obtained from fluorescence microscopy analyses. (**M**) Representative dot-blot analysis showing oxidized DJ-1 levels following incubation of recombinant DJ-1 protein with SIPE, H_2_O_2_, or SIPE + H_2_O_2_ under cell-free conditions. (**N**) Quantification of oxidized DJ-1 dot-blot signal intensity under the indicated experimental conditions. (**O**) RT-QuIC kinetic analysis of synthetic Aβ42 aggregation in the presence of recombinant DJ-1 and H_2_O_2_, monitored by Congo Red relative absorbance, illustrating the effects of SIPE on oxidation-dependent amyloid fibril formation and aggregation dynamics. Data are presented as mean ± SD from at least three independent experiments. Statistical significance was determined by one-way ANOVA followed by post hoc analysis (*** *p* < 0.001). Image magnification 20×. Scale bars as indicated in each panel.

To confirm the antioxidant effects of *Sesamum indicum* whole paste extract (SIPE), we evaluated DJ-1 oxidation at cysteine 106 (ox(C106)DJ-1) in both neuronal and astrocyte-like cell (ALC) populations within neuron–ALC co-cultures following TGC exposure. Neuronal ox(C106)DJ-1 was quantified as βIII-tubulin/ox(C106)DJ-1 double-positive cells, whereas astrocytic oxidation was defined as βIII-tubulin-negative/ox(C106)DJ-1-positive cells. Analyses were performed using flow cytometry and fluorescence microscopy. Flow cytometry analysis demonstrated that TGC exposure significantly increased neuronal DJ-1 oxidation, as evidenced by a ~25% rise in βIII-tubulin/ox(C106)DJ-1 double-positive cells compared with vehicle-treated (~8%) and SIPE-treated cells (~5%) (Figure 7A,B,E). Notably, pre-treatment with SIPE markedly attenuated this response, maintaining neuronal ox(C106)DJ-1 levels near baseline (~6%), thereby preventing TGC-induced oxidative stress in neurons. Similarly, TGC exposure significantly enhanced DJ-1 oxidation in ALCs, with an approximate 24% increase in βIII-tubulin-negative/ox(C106)DJ-1-positive cells compared with vehicle (~7%) and SIPE-treated conditions (~2%) (Figure 7C,D,F). Importantly, SIPE pre-treatment effectively suppressed this astrocytic oxidation, maintaining ox(C106)DJ-1 levels close to control values (~4%), indicating a robust inhibition of oxidative stress across both cellular compartments. These findings were consistently corroborated by fluorescence microscopy (Figure 7G–L), confirming the reduction in oxidized DJ-1 immunoreactivity in both neuronal and astrocytic populations.

To validate the antioxidant properties of SIPE and explore its potential direct reactivity toward hydrogen peroxide (H_2_O_2_), we performed a cell-free assay using recombinant DJ-1 protein. SIPE (10 μg/mL), H_2_O_2_ (10 μM), and synthetic DJ-1 (10 μM) were incubated and analyzed by dot-blot (Figure 7M,N). In the absence of both SIPE and H_2_O_2_, no oxidized DJ-1 was detected, indicating that DJ-1 remains stable under basal conditions. SIPE alone did not induce detectable DJ-1 oxidation, whereas H_2_O_2_ robustly promoted oxidation at C106. Importantly, co-incubation of SIPE with H_2_O_2_ resulted in a significant reduction in oxidized DJ-1 levels, demonstrating that SIPE directly scavenges H_2_O_2_ and protects the redox-sensitive cysteine residue (C106) of DJ-1 from oxidative modification. These findings support a direct antioxidant mechanism of SIPE beyond its cellular effects.

Given our previous evidence that DJ-1 oxidation at C106 promotes Aβ42 aggregation kinetics [48], we next evaluated whether SIPE modulates this process under oxidative conditions. Accordingly, RT-QuIC analysis using synthetic Aβ42 peptide and DJ-1 protein in the presence of H_2_O_2_ revealed the characteristic three-phase aggregation profile: a lag phase (0–6 h; 0–20% Congo Red relative absorbance), an exponential phase (6–13 h; 20–90% CR), and a plateau phase (>13 h; 90–100% CR), reflecting fibril stabilization (Figure 7O). Notably, SIPE treatment significantly reduced Aβ42 fibrillar growth in the presence of DJ-1 under oxidative conditions, predominantly during the exponential and plateau phases, resulting in an approximate 15% decrease in Congo Red relative absorbance. These findings indicate that SIPE interferes with DJ-1 oxidation-dependent amyloidogenic processes, thereby exerting an indirect anti-amyloidogenic effect through preservation of DJ-1 redox homeostasis.

### 3.10. Sesamum indicum Whole Paste Extract (SIPE) Prevents TGC-Induced Tau Phosphorylation and Caspase-3 Activation in Neuron–ALC Co-Cultures

**Figure 8:**
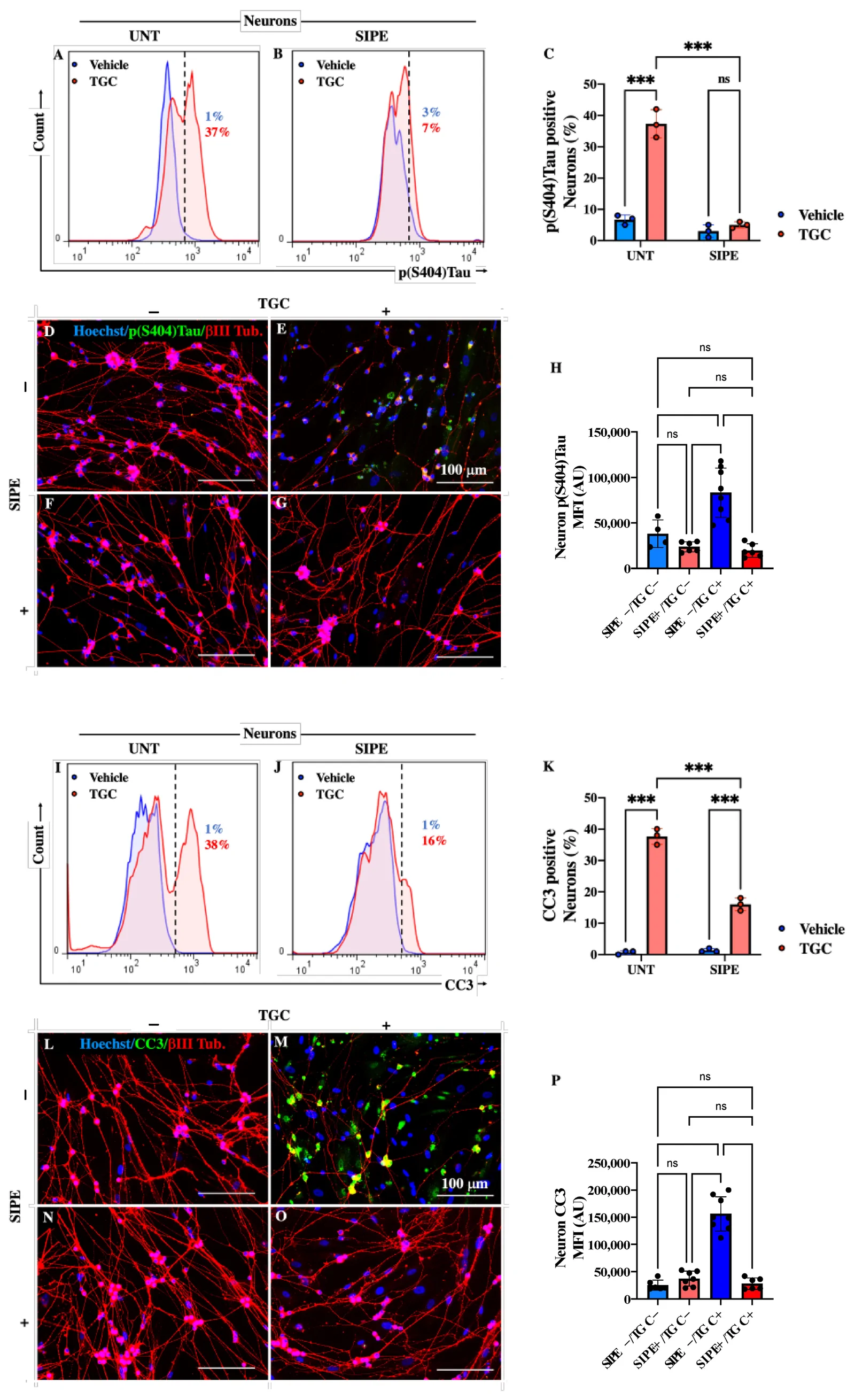
*Sesamum indicum* whole paste extract (SIPE) prevents TGC-induced Tau phosphorylation and caspase-3 activation in neuron–ALC co-cultures. (**A**,**B**) Representative flow cytometry contour plots illustrating neuronal Tau phosphorylation, defined as βIII-tubulin/p(S404)Tau double-positive cells, in neuron–ALC co-cultures exposed to TNF-α/glutamate/cortisol (TGC) in the presence or absence of SIPE (10 μg/mL). (**C**) Quantitative analysis of βIII-tubulin/p(S404)Tau double-positive neuronal populations determined by flow cytometry. (**D**–**G**) Representative fluorescence microscopy images illustrating p(S404)Tau immunoreactivity (green), βIII-tubulin-positive neurons (red), and nuclear staining (blue) in neuron–ALC co-cultures exposed to vehicle, SIPE, TGC, or SIPE + TGC conditions. (**H**) Quantification of p(S404)Tau mean fluorescence intensity (MFI) obtained from fluorescence microscopy analyses. (**I**,**J**) Representative flow cytometry contour plots illustrating neuronal cleaved caspase-3 (CC3) activation, defined as βIII-tubulin/CC3 double-positive cells, under the indicated experimental conditions. (**K**) Quantitative analysis of βIII-tubulin/CC3 double-positive neuronal populations determined by flow cytometry. (**L**–**O**) Representative fluorescence microscopy images illustrating CC3 immunoreactivity (green), βIII-tubulin-positive neurons (red), and nuclear staining (blue) in neuron–ALC co-cultures exposed to vehicle, SIPE, TGC, or SIPE + TGC conditions. (**P**) Quantification of CC3 mean fluorescence intensity (MFI) obtained from fluorescence microscopy analyses. Data are presented as mean ± SD from at least three independent experiments. Statistical significance was determined by one-way ANOVA followed by post hoc analysis (*** *p* < 0.001). Image magnification 20×. Scale bars as indicated in each panel.

To confirm the neuroprotective effects of *Sesamum indicum* whole paste extract (SIPE), we evaluated Tau phosphorylation at serine 404 (p(S404)Tau) in neuronal populations within neuron–ALC co-cultures following TGC exposure. Neuronal p(S404)Tau was quantified as βIII-tubulin/p(S404)Tau double-positive cells, and analyses were performed using flow cytometry and fluorescence microscopy. Flow cytometry analysis demonstrated that TGC exposure significantly increased neuronal Tau phosphorylation, as evidenced by a ~37% rise in βIII-tubulin/p(S404)Tau double-positive cells compared with vehicle-treated (~1%) and SIPE-treated cells (~3%) (Figure 8A–C). Notably, pre-treatment with SIPE markedly attenuated this response, maintaining neuronal p(S404)Tau levels near baseline (~7%), thereby preventing TGC-induced Tau hyperphosphorylation in neurons. These findings were consistently corroborated by fluorescence microscopy (Figure 8D–H), which confirmed reduced p(S404)Tau immunoreactivity in SIPE-treated neuronal populations. Furthermore, the regulatory effect of SIPE on Tau phosphorylation under TGC exposure was confirmed by assessing the pathological p(S202/T205)Tau epitope. Notably, a comparable protective response was observed, providing additional evidence that SIPE broadly attenuates Tau hyperphosphorylation associated with chronic stress-induced neurodegenerative processes (Supplementary Figure S3).

To further determine whether SIPE protects against TGC-induced neuronal apoptosis, we evaluated cleaved caspase-3 (CC3) as a marker of apoptotic activation in neuron–ALC co-cultures. Neuronal CC3 was quantified as βIII-tubulin/CC3 double-positive cells using flow cytometry and fluorescence microscopy. TGC exposure significantly increased neuronal CC3 levels, as evidenced by a ~38% rise in βIII-tubulin/CC3 double-positive cells compared with vehicle-treated (~1%) and SIPE-treated cells (~1%) (Figure 8I–K). Importantly, SIPE pre-treatment markedly attenuated this response, reducing CC3-positive neuronal populations to approximately half of TGC-induced levels (~16%), indicating a strong inhibition of caspase-3 activation and downstream apoptotic signaling. These results were further validated by fluorescence microscopy (Figure 8L–P), confirming the reduction in apoptotic markers in SIPE-treated neuron–ALC co-cultures.

### 3.11. Sesamum indicum Whole Paste Extract (SIPE) Prevents TGC-Induced Neuronal Loss and Attenuates Mitochondrial Dysfunction and Superoxide Generation in Neuron–ALC Spheroids

**Figure 9:**
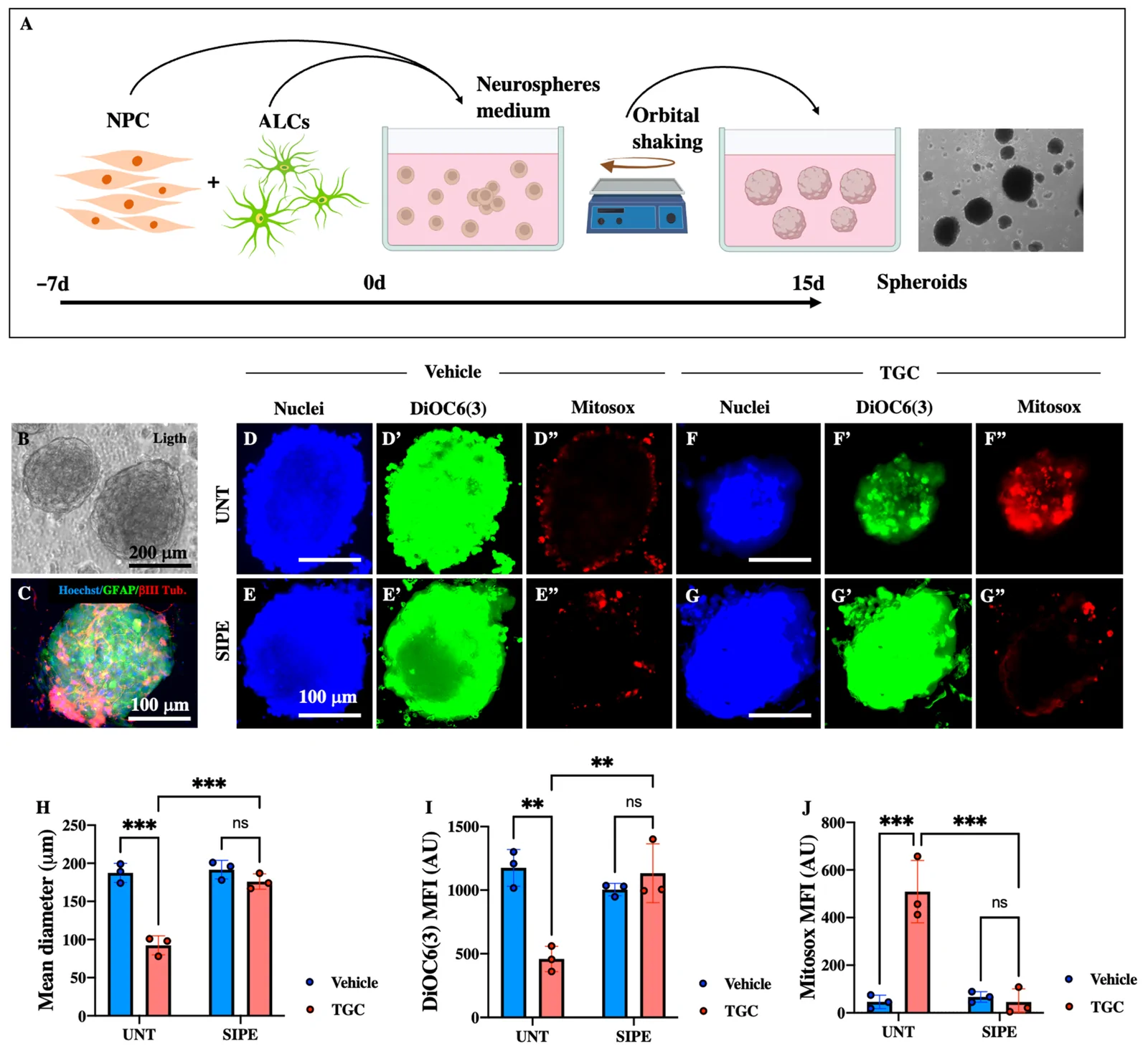
*Sesamum indicum* whole paste extract (SIPE) preserves spheroid integrity and attenuates TGC-induced mitochondrial dysfunction and oxidative stress in neuron–ALC spheroids. (**A**–**C**) Schematic representation of the generation of neuron–astrocyte-like cell (ALC) spheroids using floating 3D co-culture conditions and the subsequent experimental exposure to TNF-α/glutamate/cortisol (TGC) in the presence or absence of SIPE (10 μg/mL). (**D**–**G**) Representative fluorescence microscopy images of neuron–ALC spheroids stained with Hoechst (blue) illustrating spheroid architecture and diameter under vehicle, SIPE, TGC, and SIPE + TGC conditions. (**H**) Quantification of spheroid diameter based on Hoechst-positive nuclei distribution. (**D′**–**G′**) Representative fluorescence microscopy images showing DiOC6(3) staining (green) for mitochondrial membrane potential (ΔΨm) in neuron–ALC spheroids exposed to the indicated experimental conditions. (**I**) Quantification of DiOC6(3) mean fluorescence intensity (MFI) obtained from fluorescence microscopy analyses. (**D″**–**G″**) Representative fluorescence microscopy images showing MitoSOX staining (red) for mitochondrial superoxide production in neuron–ALC spheroids under the indicated experimental conditions. (**J**) Quantification of MitoSOX mean fluorescence intensity (MFI) obtained from fluorescence microscopy analyses. Data are presented as mean ± SD from at least three independent experiments. Statistical significance was determined by one-way ANOVA followed by post hoc analysis (** *p* < 0.01, *** *p* < 0.001). Image magnification 40×. Scale bars as indicated in each panel.

To validate the protective effects of the *Sesamum indicum* whole paste extract (SIPE; 10 μg/mL), we established a 3D culture system by generating neuron–astrocyte-like cell (ALC) spheroids using floating co-cultures (Figure 9A–C). Subsequently, spheroid integrity was evaluated through spheroid diameter measurements based on nuclear (Hoechst) staining, while mitochondrial membrane potential (ΔΨm) and mitochondrial superoxide production were assessed using the DiOC6(3) and MitoSOX probes, respectively, in spheroids exposed to TGC in the presence or absence of SIPE. All analyses were performed by fluorescence microscopy. Microscopic analysis revealed that TGC exposure induced a pronounced reduction in spheroid diameter, decreasing by approximately 50% (~92 μm; Figure 9F,H) compared with untreated control spheroids (~187 μm; Figure 9D,H) and SIPE-treated spheroids (~191 μm; Figure 9E,H). Notably, SIPE pre-treatment markedly preserved spheroid architecture, maintaining spheroid diameter at levels comparable to untreated controls (~176 μm; Figure 9G,H). Similarly, TGC exposure caused a substantial loss of ΔΨm, with an approximate 60% reduction relative to untreated control spheroids (Figure 9D’,I) and SIPE-only treated cultures (Figure 9E’,I). Importantly, SIPE pre-treatment effectively preserved mitochondrial membrane potential, maintaining ΔΨm levels close to those observed in untreated controls (Figure 9G’,I). Consistent with these findings, TGC exposure dramatically increased mitochondrial superoxide production in neuron–ALC spheroids (~1000%; Figure 9F″,J) compared with untreated and SIPE-treated cultures (Figure 9D″,E″,J). Remarkably, SIPE pre-treatment almost completely abolished this increase, maintaining mitochondrial superoxide levels near baseline values (Figure 9G″,J), thereby indicating a strong attenuation of mitochondrial oxidative stress. Collectively, these findings demonstrate that SIPE preserves spheroid structural integrity and protects against TGC-induced mitochondrial dysfunction and oxidative stress in neuron–ALC 3D cultures.

### 3.12. Sesamum indicum Whole Paste Extract (SIPE) Mitigates TGC-Induced NF-κB Activation, DJ-1 Oxidation, Amyloidogenic Signaling, Tau Phosphorylation, Caspase-3 Activation, and Cytokine Release in Neuron–ALC Spheroids

**Figure 10:**
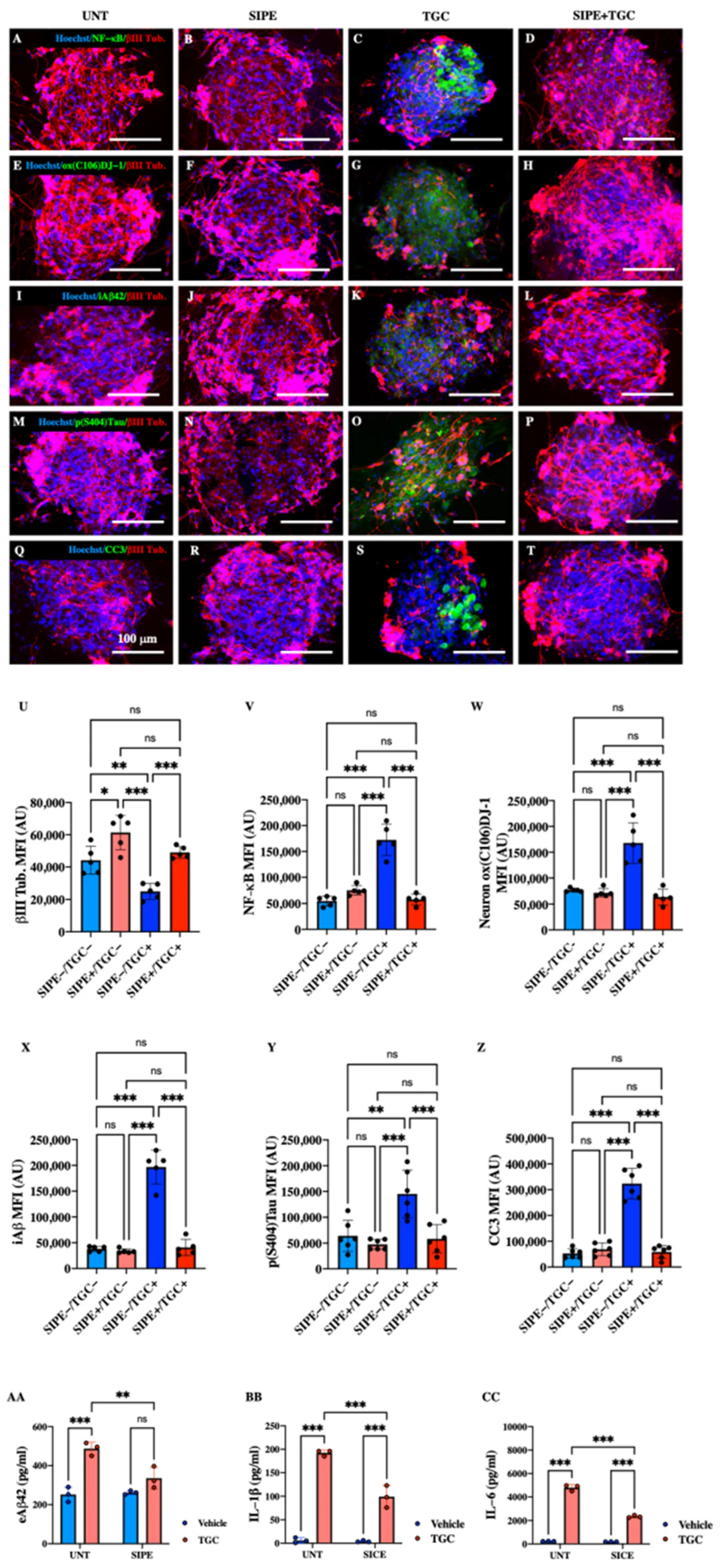
*Sesamum indicum* whole paste extract (SIPE) mitigates TGC-induced inflammatory signaling, oxidative stress, amyloidogenic processing, tauopathy, apoptotic activation, and cytokine release in neuron–ALC spheroids. (**A**–**T**) Representative fluorescence microscopy images illustrating βIII-tubulin immunoreactivity (red) and nuclear staining (blue) in neuron–ALC spheroids exposed to vehicle, SIPE, TGC, or SIPE + TGC conditions. (**A**–**D**) Representative fluorescence microscopy images showing NF-κB immunoreactivity (green), βIII-tubulin-positive neurons (red), and nuclear staining (blue) under the indicated experimental conditions. (**E**–**H**) Representative fluorescence microscopy images illustrating oxidized DJ-1 (ox(C106)DJ-1) immunoreactivity (green), βIII-tubulin-positive neurons (red), and nuclear staining (blue) in neuron–ALC spheroids. (**I**–**L**) Representative fluorescence microscopy images showing intracellular Aβ42 immunoreactivity (green), βIII-tubulin-positive neurons (red), and nuclear staining (blue) under the indicated experimental conditions. (**M**–**P**) Representative fluorescence microscopy images illustrating p(S404)Tau immunoreactivity (green), βIII-tubulin-positive neurons (red), and nuclear staining (blue) in neuron–ALC spheroids exposed to vehicle, SIPE, TGC, or SIPE + TGC conditions. (**Q**–**T**) Representative fluorescence microscopy images showing cleaved caspase-3 (CC3) immunoreactivity (green), βIII-tubulin-positive neurons (red), and nuclear staining (blue) under the indicated experimental conditions. (**U**–**Z**) Quantitative analyses of βIII-tubulin, NF-κB, ox(C106)DJ-1, intracellular Aβ42, p(S404)Tau, and CC3 mean fluorescence intensity (MFI) obtained from fluorescence microscopy analyses. (**AA**) Quantification of extracellular Aβ42 (eAβ42) levels in neuron–ALC spheroid supernatants under the indicated experimental conditions. (**BB**,**CC**) Quantification of IL-1β and IL-6 release in neuron–ALC spheroid supernatants exposed to vehicle, SIPE, TGC, or SIPE + TGC conditions. Data are presented as mean ± SD from at least three independent experiments. Statistical significance was determined by one-way ANOVA followed by post hoc analysis (* *p* < 0.05, ** *p* < 0.01, *** *p* < 0.001). Image magnification 20×. Scale bars as indicated in each panel.

To further investigate the neuroprotective effects of *Sesamum indicum* whole paste extract (SIPE; 10 μg/mL) in the 3D neuron–ALC spheroid model exposed to TGC, we analyzed multiple hallmarks associated with neuroinflammation, oxidative stress, Alzheimer’s disease-related pathology, and apoptotic signaling by fluorescence microscopy. Initially, neuronal integrity was evaluated through βIII-tubulin immunoreactivity. Quantitative analysis demonstrated that TGC exposure markedly reduced the neuronal population, producing an approximate 43% decrease in βIII-tubulin-positive cells (Figure 10B,F,J,N,R,U) compared with vehicle-treated controls (Figure 10A,E,I,M,Q,U) and SIPE-treated spheroids (Figure 10C,G,K,O,S,U). Importantly, SIPE pre-treatment largely preserved neuronal identity and structural integrity, maintaining βIII-tubulin reactivity at levels comparable to untreated controls (Figure 10D,H,L,P,T,U), thereby preventing TGC-induced neuronal degeneration.

Evaluation of the pro-inflammatory transcription factor NF-κB (p65) revealed a robust inflammatory response following TGC exposure, with an approximately 215% increase in NF-κB activation (Figure 10B,V) relative to vehicle- and SIPE-treated spheroids (Figure 10A,C,V). Remarkably, SIPE markedly suppressed this response, maintaining NF-κB activation near basal levels (Figure 10D,V), thus attenuating TGC-induced inflammatory signaling. Likewise, oxidative stress-associated damage was assessed by quantifying oxidized DJ-1 (ox(C106)DJ-1). TGC treatment significantly enhanced DJ-1 oxidation, increasing ox(C106)DJ-1 immunoreactivity by approximately 117% (Figure 10F,W) compared with control and SIPE-treated conditions (Figure 10E,G,W). In contrast, SIPE pre-treatment effectively preserved DJ-1 redox homeostasis, maintaining oxidation levels close to those observed in untreated spheroids (Figure 10H,W), indicative of reduced oxidative stress burden.

Analysis of intracellular amyloid beta accumulation revealed that TGC strongly promoted amyloidogenic processing, producing an approximately 417% increase in intracellular Aβ42 (iAβ42) levels (Figure 10J,X) relative to vehicle- and SIPE-treated groups (Figure 10I,K,X). Notably, SIPE markedly diminished this amyloidogenic response, restoring iAβ42 levels toward basal conditions (Figure 10L,X), thereby limiting TGC-induced amyloid accumulation. Similarly, assessment of Tau phosphorylation demonstrated that TGC significantly increased p(S404)Tau immunoreactivity by approximately 126% (Figure 10N,Y) compared with control conditions (Figure 10M,O,Y). Pre-treatment with SIPE substantially reduced Tau hyperphosphorylation, maintaining pTau levels close to those detected in untreated spheroids (Figure 10P,Y), suggesting protection against TGC-associated tauopathic alterations. Consistent with these findings, TGC exposure triggered a pronounced apoptotic response, evidenced by an approximately 512% increase in cleaved caspase-3 (CC3) activation (Figure 10R,Z) relative to vehicle- and SIPE-treated spheroids (Figure 10Q,S,Z). Importantly, SIPE significantly restrained caspase-3 activation, preserving CC3 levels near baseline values (Figure 10T,Z), thereby preventing TGC-induced apoptotic cell death.

To confirm the anti-amyloidogenic effects of SIPE, extracellular Aβ42 (eAβ42) release was quantified in spheroid supernatants. Flow cytometric analysis demonstrated that TGC markedly increased eAβ42 secretion (487 ± 34 pg/mL) compared with vehicle-treated (252 ± 37 pg/mL) and SIPE-treated spheroids (262 ± 10 pg/mL) (Figure 10AA). Notably, SIPE pre-treatment significantly attenuated this increase, reducing extracellular Aβ42 levels to approximately 335 ± 56 pg/mL, indicative of a substantial reduction in extracellular amyloid burden. Finally, the anti-inflammatory effects of SIPE were further validated through the quantification of the pro-inflammatory cytokines IL-1β and IL-6. TGC exposure dramatically increased IL-1β release (192 ± 7 pg/mL) relative to vehicle-treated (8 ± 6 pg/mL) and SIPE-treated spheroids (4 ± 2 pg/mL) (Figure 10BB). Although IL-1β levels remained elevated following SIPE pre-treatment, they were significantly reduced compared with TGC alone (99 ± 23 pg/mL), supporting a partial suppression of inflammatory signaling. Similarly, TGC markedly elevated IL-6 secretion (4800 ± 261 pg/mL) compared with vehicle-treated (231 ± 16 pg/mL) and SIPE-treated conditions (161 ± 3 pg/mL) (Figure 10CC). Importantly, SIPE significantly reduced IL-6 release (2330 ± 103 pg/mL), further supporting its capacity to mitigate neuroinflammatory responses in neuron–ALC spheroids.

Collectively, these findings demonstrate that SIPE exerts broad neuroprotective effects in the 3D neuron–ALC spheroid model by attenuating inflammatory signaling, oxidative stress, amyloidogenic processing, tauopathy, apoptotic activation, and cytokine release induced by TGC exposure.

### 3.13. Major Phytochemical Constituents of S. indicum Whole Paste Exhibit Complementary Multitarget Interactions with Proteins Involved in Chronic Stress-Associated Neurodegeneration

**Figure 11:**
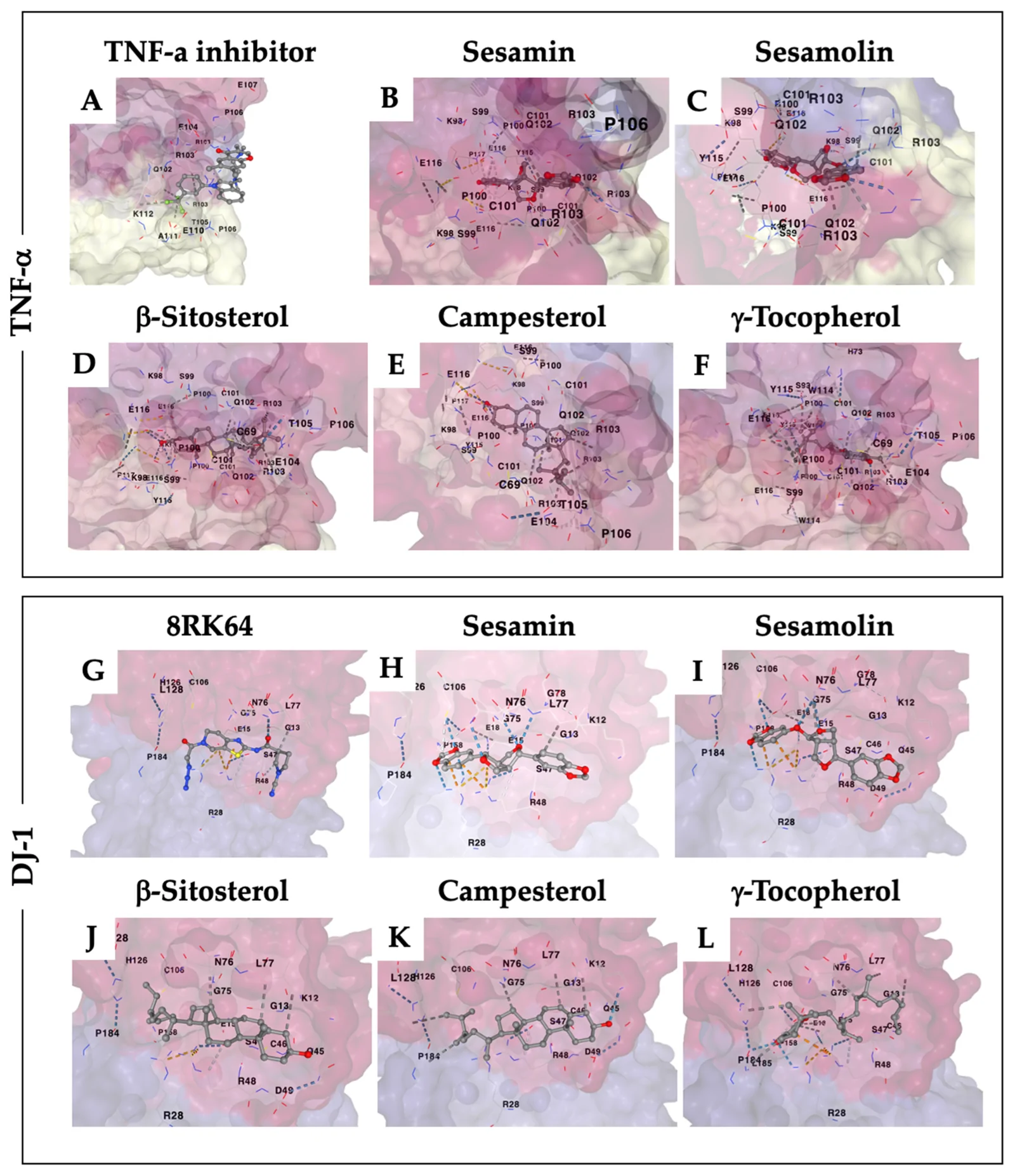

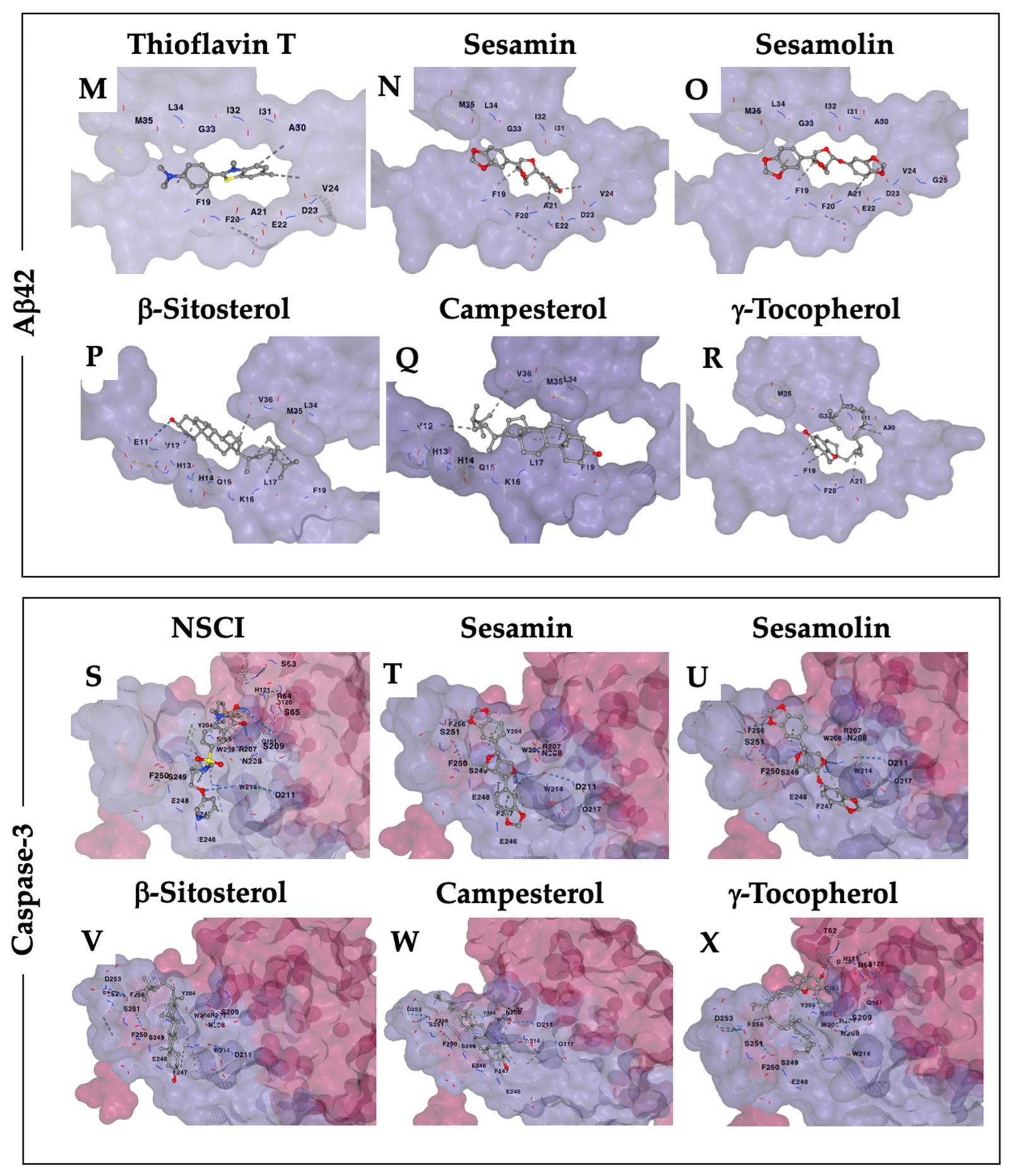
Molecular docking analysis of the major bioactive constituents of *Sesamum indicum* whole paste against key molecular targets involved in chronic stress-associated neurodegeneration. Representative docking poses illustrating the interactions of the principal *Sesamum indicum* whole paste phytochemicals ((+)-sesamin, sesamolin, β-sitosterol, campesterol, and γ-tocopherol) with TNF-α (**A**–**F**), DJ-1 (**G**–**L**), amyloid-β42 (Aβ42) (**M**–**R**), and caspase-3 (**S**–**X**). Docking analyses were performed using CB-Dock3, and binding affinities were compared with those of previously reported reference ligands for each target, including the TNF-α inhibitor (CID: 16079006), the DJ-1 ligand 8RK64 (CID: 162677482), Thioflavin T for Aβ42, and NSCI (CID: 11591540) for caspase-3. Representative ligand-binding conformations are shown within the predicted functional cavities together with the principal interacting residues. Although individual phytochemicals exhibited distinct binding affinities, all compounds occupied functionally relevant regions associated with inflammatory signaling, oxidative stress regulation, amyloid aggregation, and apoptotic activation. Interaction residues and docking parameters are summarized in Table 4 and Supplementary Table S2.

Considering that the extract from *Sesamum indicum* whole paste consistently exhibited the strongest neuroprotective effects across all experimental models evaluated in this study, we next investigated whether *Sesamum indicum* whole paste’s major bioactive constituents could directly interact with molecular targets implicated in chronic stress-induced neurodegeneration. Phytochemical characterization revealed that SIPE was markedly enriched in several bioactive metabolites, particularly the lignans (+)-sesamin and sesamolin, the phytosterols β-sitosterol and campesterol, and the antioxidant γ-tocopherol. Together, these compounds represent the predominant phytochemical classes identified in SIPE and collectively define its antioxidant and neuroprotective phytochemical matrix. To determine whether the biological activity of SIPE could be explained by the coordinated action of multiple enriched constituents rather than by its major lignan alone, we expanded the molecular docking analysis to include these five representative phytochemicals. Each compound was evaluated against molecular targets critically involved in chronic stress-associated neurodegeneration, including TNF-α, DJ-1, amyloid-β42 (Aβ42), and caspase-3. For each target, docking performance was compared with that of well-characterized reference ligands previously reported to occupy the same functional binding pockets, including the TNF-α inhibitor CID:16079006, the DJ-1 modulator 8RK64 (CID:162677482), Thioflavin T for Aβ42 aggregation-associated regions, and NSCI (CID:11591540) for caspase-3 (Figure 11; Table 4; Supplementary Table S2).

Molecular docking analyses demonstrated that the principal bioactive constituents enriched in *Sesamum indicum* whole paste collectively exhibited favorable interaction profiles with proteins critically involved in chronic stress-associated neurodegeneration, including TNF-α, DJ-1, amyloid-β42 (Aβ42), and caspase-3 (Figure 11A–X, Table 4). Although each phytochemical displayed a distinct affinity profile depending on the molecular target, all five compounds occupied functionally relevant cavities previously identified by established reference ligands, supporting the hypothesis that the biological activity of SIPE arises from the coordinated action of multiple bioactive constituents rather than from a single predominant molecule (Figure 11; Table 4; Supplementary Table S2).

Among the evaluated phytochemicals, the lignans sesamin and sesamolin exhibited the most favorable multitarget interaction profiles. Sesamolin displayed the highest overall binding affinities, surpassing the reference TNF-α inhibitor (−9.0 vs. −8.0 kcal/mol) and the reference DJ-1 ligand 8RK64 (−7.7 vs. −7.0 kcal/mol), while also exhibiting the strongest interaction with Aβ42 (−6.2 kcal/mol) and maintaining a high affinity for caspase-3 (−8.1 kcal/mol). Likewise, sesamin demonstrated consistently favorable docking scores across all evaluated proteins, including TNF-α (−7.7 kcal/mol), DJ-1 (−7.5 kcal/mol), Aβ42 (−5.0 kcal/mol), and caspase-3 (−8.3 kcal/mol), confirming its broad multitarget interaction profile. Both lignans occupied the canonical TNF-α trimeric interface, interacting with conserved residues including GLN102, ARG103, GLU104, THR105, PRO106, GLU107, and LYS112, suggesting stabilization of a functionally relevant inhibitory region associated with modulation of TNF-α signaling. Similarly, both lignans established extensive interactions within the redox regulatory cavity of DJ-1, including residues surrounding the highly oxidation-sensitive Cys106 microenvironment (GLN45, CYS46, SER47, ARG48, ASP49, ASN76, GLN80, CYS106, THR110, and HIS126) (Figure 11; Table 4; Supplementary Table S2). Because irreversible oxidation of Cys106 represents a critical event leading to DJ-1 dysfunction during oxidative stress, these interactions support the possibility that sesame lignans contribute to preserving the structural integrity and antioxidant functions of this master regulator of cellular redox homeostasis.

Docking simulations against Aβ42 further demonstrated that the phytochemicals preferentially interacted with the hydrophobic aggregation core of the peptide. In particular, sesamolin exhibited greater affinity than the reference amyloid-binding probe Thioflavin T (−6.2 vs. −5.0 kcal/mol), whereas sesamin displayed a comparable interaction profile. Both lignans established contacts with residues involved in amyloid nucleation and fibrillization, including HIS13, HIS14, GLN15, LYS16, LEU17, PHE19, PHE20, ALA21, VAL24, ILE31, ILE32, GLY33, LEU34, and MET35, suggesting that these compounds may interfere with early aggregation-prone conformations of Aβ42. Likewise, docking against caspase-3 revealed that both lignans occupied the catalytic-associated cavity recognized by the reference inhibitor NSCI. Sesamin (−8.3 kcal/mol) and sesamolin (−8.1 kcal/mol) displayed docking scores approaching that of the reference ligand (−8.8 kcal/mol) and shared multiple interaction residues within the catalytic region, including TRP206, ARG207, ASN208, SER209, TRP214, GLN217, GLU246, PHE247, GLU248, SER249, and PHE250 (Figure 11; Table 4; Supplementary Table S2), supporting a potential role in modulating caspase-3 activation and apoptotic signaling.

In addition to the lignans, the phytosterols β-sitosterol and campesterol, together with γ-tocopherol, also demonstrated stable interactions with all evaluated molecular targets. Although their binding affinities were generally lower than those observed for sesamin and sesamolin, these compounds consistently occupied the same functional cavities identified by the corresponding reference ligands and shared numerous contact residues involved in inflammatory regulation, oxidative stress responses, amyloid assembly, and apoptosis (Figure 11; Table 4; Supplementary Table S2). Notably, all five phytochemicals converged on highly conserved functional regions, including the TNF-α trimeric interface, the Cys106-centered redox regulatory cavity of DJ-1, the hydrophobic aggregation core of Aβ42, and the catalytic-associated pocket of caspase-3, indicating a remarkable degree of mechanistic complementarity despite their structural diversity. Collectively, these in silico findings support the concept that *Sesamum indicum* whole paste functions as a multitarget phytochemical matrix rather than as a source of a single active compound. While the lignans contributed the strongest individual binding affinities, the phytosterols and γ-tocopherol expanded the spectrum of molecular interactions by reinforcing complementary binding within the same pathogenic pathways. This cooperative interaction network provides a mechanistic explanation for the robust neuroprotective effects observed experimentally in SIPE-treated neuron–astrocyte co-cultures and three-dimensional spheroids, supporting the hypothesis that the biological efficacy of SIPE emerges from the synergistic modulation of interconnected processes governing neuroinflammation, oxidative stress, mitochondrial dysfunction, amyloidogenesis, and apoptosis during chronic stress-associated neurodegeneration.

## 4. Discussion

The present study was designed to investigate whether traditionally processed sesame-derived products can protect neuron–glia networks against neurodegenerative alterations induced by a multifactorial stress paradigm integrating inflammatory, endocrine, and excitotoxic insults. To address this objective, we first established a biologically relevant in vitro platform composed of iPSC-derived neurons and Wharton’s jelly-derived astrocyte-like cells (ALCs). Collectively, the high differentiation efficiencies obtained in both lineages supported the generation of a stable and reproducible neuron–glia platform suitable for modeling stress-associated neuropathological processes. The incorporation of ALCs represents a particularly relevant aspect of the present experimental design. Although neuronal dysfunction has historically dominated the study of neurodegenerative disorders, accumulating evidence indicates that astrocytes actively regulate glutamatergic homeostasis, neurotrophic support, inflammatory signaling, antioxidant defense mechanisms, and metabolic coupling with neurons [49]. Consequently, chronic stress-associated alterations are unlikely to be fully reproduced in neuronal monocultures. By establishing a neuron–ALC co-culture system, we sought to recreate key aspects of neuron–glia communication that are increasingly recognized as critical determinants of neuronal vulnerability and resilience during stress-related conditions. Having established this biologically relevant neuron–glia platform, our next objective was to develop a multifactorial stress paradigm integrating three major pathological mechanisms frequently associated with prolonged stress exposure in vivo, namely inflammatory activation, glucocorticoid dysregulation, and glutamate-mediated excitotoxicity [50]. Accordingly, co-cultures were exposed to a combination of TNF-α, glutamate, and cortisol (TGC), representing inflammatory, excitotoxic, and endocrine components, respectively. Although this experimental paradigm does not recapitulate the full biological complexity of chronic stress, it provides a reductionist approach to investigate the convergence of these interconnected pathways and their contribution to oxidative stress, mitochondrial dysfunction, and neurodegeneration [4]. Consistent with this rationale, TGC exposure significantly reduced MTT reduction capacity while simultaneously increasing NBT reduction, indicating the development of mitochondrial dysfunction accompanied by enhanced superoxide generation. These findings agree with growing evidence demonstrating that the convergence of inflammatory, endocrine, and excitotoxic insults promotes mitochondrial impairment, disrupts electron transport chain activity, and enhances reactive oxygen species (ROS) production, all of which are frequently observed in chronic stress-related disorders [51]. From a pathophysiological perspective, the simultaneous induction of mitochondrial dysfunction and oxidative stress is particularly relevant because both mechanisms are increasingly recognized as central drivers of neuronal dysfunction and are shared by several neurodegenerative disorders, including Alzheimer’s disease, where they contribute to amyloidogenesis, Tau pathology, neuroinflammation, and progressive neuronal loss [52].

Having established a neuron–astrocyte co-culture exposed to convergent stress-associated stimuli, we next examined whether sesame-derived products differed in their phytochemical composition and whether such differences could account for their subsequent biological activity. Remarkably, whole sesame paste emerged as the most phytochemically enriched preparation among all products evaluated. Compared with raw seeds and sesame oil, the traditional transformation of sesame into paste generated a complex matrix enriched in polyphenols, lignans, phytosterols, tocopherols, and unsaturated fatty acids. These findings suggest that artisanal processing does not merely preserve sesame bioactives but may optimize the concentration and availability of compounds with recognized antioxidant, anti-inflammatory, and neuroprotective properties [53]. Importantly, the observed phytochemical enrichment was not merely a compositional finding but appeared to closely parallel the biological efficacy of the different sesame-derived preparations. This association suggests that the neuroprotective effects observed throughout the study are intrinsically linked to the preservation of a complex phytochemical architecture generated during traditional sesame processing. Consequently, the subsequent experiments were designed to determine whether this enriched phytochemical matrix could effectively counteract the pathological cascade initiated by chronic stress exposure. One of the most striking observations was the substantial increase in total polyphenol content detected in sesame paste. Specifically, sesame paste contained approximately 25% more total polyphenols than raw seeds and more than five times the amount detected in sesame oil. This finding is particularly relevant because polyphenols constitute one of the principal antioxidant defense systems naturally present in plant-derived foods and are known to neutralize ROS, modulate redox-sensitive signaling pathways, and preserve mitochondrial function [39,40]. Conversely, the markedly lower polyphenol content detected in oil suggests that lipid extraction concentrates fatty acids while simultaneously reducing the abundance of polar antioxidant metabolites.

In parallel, sesame paste exhibited the highest concentrations of sesamin and sesamolin, two characteristic sesame lignans extensively associated with activation of endogenous antioxidant responses, inhibition of NF-κB-dependent inflammatory signaling, preservation of mitochondrial homeostasis, regulation of lipid metabolism, and attenuation of amyloidogenic pathways [54,55,56]. Therefore, the enrichment of both lignans strongly supports the concept that whole sesame paste may function as a multitarget neuroprotective matrix capable of simultaneously modulating several pathogenic pathways implicated in chronic stress-associated neurodegeneration. The phytosterol profile further reinforces the biological relevance of SIPE. β-Sitosterol, campesterol, stigmasterol, and isofucosterol were consistently more abundant in sesame paste than in seed and oil preparations. Increasing evidence indicates that phytosterols exert anti-inflammatory and antioxidant effects while modulating glial activation and supporting neuronal survival pathways [57]. Likewise, sesame paste exhibited elevated levels of γ-tocopherol, a lipid-soluble antioxidant with a remarkable capacity to scavenge reactive nitrogen species and protect membrane lipids from oxidative damage [58]. Since neuronal membranes are particularly vulnerable to oxidative injury due to their high content of polyunsaturated fatty acids, increased γ-tocopherol availability may represent an additional mechanism contributing to cellular resilience under chronic stress conditions. Although sesame oil retained a favorable fatty acid composition characterized by high levels of linoleic and oleic acids, its overall phytochemical diversity was substantially lower than that of sesame paste. Consequently, the superior efficacy of SIPE is unlikely to be explained by lipid composition alone. Rather, its biological activity most likely arises from preservation of a complete phytochemical matrix in which unsaturated fatty acids coexist with lignans, polyphenols, phytosterols, and tocopherols.

Importantly, the preservation of mitochondrial function observed following SIPE treatment was not an isolated phenomenon but was accompanied by profound effects on cellular homeostasis throughout the neuron–ALC network. Indeed, chronic exposure to TGC induced a significant reduction in βIII-tubulin-positive neuronal populations, indicating that the oxidative and bioenergetic disturbances generated by the stress cocktail were sufficient to compromise neuronal survival. This observation is consistent with extensive evidence demonstrating that chronic stress promotes neuronal vulnerability through the convergence of cytotoxic signaling [59]. Consequently, the ability of SIPE to preserve neuronal populations suggests that its beneficial effects on mitochondrial integrity translate into functional protection of neuronal networks rather than merely correcting isolated metabolic parameters. Notably, neuronal preservation was accompanied by maintenance of pro-BDNF expression, indicating preservation of neurotrophic signaling under stress conditions. Although mature BDNF is traditionally associated with neuronal survival and synaptic plasticity, increasing evidence suggests that pro-BDNF also participates in neuronal homeostasis, differentiation, and adaptive responses to cellular stress. Therefore, the marked reduction in pro-BDNF induced by TGC exposure likely reflects a profound disruption of neurotrophic support mechanisms. Given that impaired BDNF signaling represents a common pathological feature of chronic stress, cognitive decline, and Alzheimer’s disease [60,61], the ability of SIPE to preserve pro-BDNF expression may contribute not only to neuronal resilience but also to the maintenance of synaptic stability and adaptive stress responses.

Beyond its effects on neurons, SIPE also exerted a substantial modulatory influence on astrocytic biology. Astrocytes are increasingly recognized as central regulators of brain homeostasis, controlling glutamate clearance, antioxidant defenses, inflammatory signaling, neurotrophic factor production, and metabolic support to neurons [62]. Under pathological conditions, however, astrocytes undergo a process of reactive transformation commonly referred to as gliosis, characterized by increased expression of GFAP, S100β, and vimentin [63]. Although reactive gliosis may initially represent an adaptive response aimed at limiting tissue damage, persistent activation frequently contributes to neuroinflammation, excitotoxicity, and progressive neuronal dysfunction. In agreement with this concept, TGC exposure induced a pronounced reactive astrocytic phenotype characterized by increased GFAP, S100β, and vimentin expression together with a reduction in glutamine synthetase (GS). The reduction in GS deserves particular attention because this enzyme represents a critical component of the glutamate–glutamine cycle, catalyzing the conversion of glutamate into glutamine and thereby preventing excitotoxic accumulation of extracellular glutamate [64]. Consequently, decreased GS expression may reflect impaired astrocytic metabolic competence and diminished glutamatergic buffering capacity, both of which are increasingly associated with chronic stress-related neurodegeneration [65,66]. Remarkably, SIPE prevented both astrocytic reactivity and GS loss, suggesting preservation of astrocytic homeostasis and maintenance of essential neuron–glia metabolic interactions. Thus, the protective effects of SIPE appear to extend beyond neuronal preservation and include stabilization of the astrocytic compartment that supports neuronal survival.

The attenuation of neuronal and astrocytic pathology was subsequently accompanied by a robust suppression of inflammatory signaling. Among the molecular pathways activated by chronic stress, NF-κB occupies a particularly relevant position because it integrates oxidative stress, cytokine signaling, mitochondrial dysfunction, and glial activation into a coordinated inflammatory response [67]. In the present study, TGC induced marked NF-κB activation in both neuronal and astrocytic compartments, indicating that inflammatory signaling extended beyond glial cells and directly affected neuronal populations and importantly, SIPE effectively prevented NF-κB activation in both cellular populations. This dual action is particularly significant because suppression of neuronal NF-κB signaling may directly reduce neuronal vulnerability, whereas inhibition of astrocytic NF-κB activation may limit the production of inflammatory mediators that perpetuate neurotoxicity. Consistent with this interpretation, SIPE markedly reduced the release of IL-1β and IL-6, two cytokines extensively implicated in chronic stress pathology, synaptic dysfunction, and cognitive impairment/neurodegeneration [68]. Therefore, the anti-inflammatory effects of SIPE appear to operate at both intracellular and extracellular levels, ultimately contributing to preservation of neuron–glia homeostasis.

Consistent with this notion, TGC exposure induced significant accumulation of intracellular Aβ42 in both neurons and ALCs, accompanied by increased extracellular Aβ42 release. The observation that intracellular Aβ42 accumulated in both cellular populations is particularly relevant because astrocytes are increasingly recognized as active participants in amyloid metabolism, clearance, and inflammatory amplification [69,70,71]. Accordingly, dysfunction of either neuronal or astrocytic compartments may contribute to the progressive establishment of an amyloidogenic microenvironment. Remarkably, SIPE reduced both intracellular and extracellular Aβ42 levels, suggesting broad modulation of amyloidogenic pathways operating across neuron–glia networks. Furthermore, RT-QuIC analyses demonstrated that SIPE directly interferes with Aβ42 aggregation kinetics. More specifically, the reduction observed during the exponential and plateau phases indicates inhibition of fibril elongation and stabilization rather than a simple delay in aggregation onset. These findings are particularly relevant because several naturally occurring polyphenols and lignans have been reported to interact with amyloidogenic proteins, destabilize β-sheet-rich structures, and redirect aggregation toward less toxic conformations [72,73]. Consequently, SIPE appears to exert both indirect anti-amyloidogenic effects through preservation of cellular homeostasis and direct effects on amyloid assembly dynamics.

Mechanistically, oxidative stress may represent a critical link connecting chronic stress with amyloidogenesis. In this context, DJ-1 emerges as a particularly relevant molecular sensor because oxidation of its highly reactive Cys106 residue occurs in response to mitochondrial dysfunction and excessive ROS generation. Under physiological conditions, DJ-1 contributes to antioxidant defenses, mitochondrial integrity, inflammatory regulation, and proteostasis maintenance. However, excessive oxidation compromises these cytoprotective functions and has been increasingly associated with neurodegenerative progression [74]. Although DJ-1 was initially identified as a neuroprotective protein in Parkinson’s disease, accumulating evidence demonstrates that alterations in its redox state also play a pivotal role in Alzheimer’s disease [20,21]. Oxidative overoxidation of Cys106 impairs DJ-1-mediated activation of antioxidant pathways, including the Nrf2 axis, disrupts mitochondrial quality control, and amplifies NF-κB-dependent inflammatory signaling. These events collectively create a permissive environment for enhanced amyloidogenic APP processing, intracellular Aβ accumulation, Tau hyperphosphorylation, and synaptic dysfunction, all of which are recognized hallmarks of Alzheimer’s disease. Moreover, decreased functional DJ-1 and increased oxidized DJ-1 have been reported to be associated with Aβ in *postmortem* brain samples from patients with Alzheimer’s disease [19,48], suggesting that DJ-1 redox status may represent both a mechanistic determinant and a potential biomarker of disease progression.

Consistent with the oxidative alterations induced by TGC, we observed a marked increase in ox(C106)DJ-1 in both neuronal and astrocytic compartments. Remarkably, SIPE almost completely prevented DJ-1 oxidation, restoring ox(C106)DJ-1 levels to values comparable to untreated controls. This observation substantially extends the antioxidant effects previously demonstrated through MTT, NBT, DiOC6(3), and MitoSOX analyses and suggests that SIPE not only reduces ROS production but also preserves critical redox-sensitive proteins involved in cellular defense mechanisms. The cell-free experiments provide particularly compelling mechanistic support for this interpretation. Under these conditions, SIPE directly reduced H_2_O_2_-induced DJ-1 oxidation, indicating intrinsic antioxidant activity independent of cellular metabolism. Preservation of DJ-1 in its functional redox state may therefore have consequences extending beyond ROS scavenging, as it could sustain mitochondrial homeostasis, maintain proteostatic pathways, and indirectly limit the molecular events that drive amyloidogenesis and tauopathy under chronic stress conditions. Consistent with this relationship, RT-QuIC analyses performed in the presence of recombinant DJ-1 and H_2_O_2_ demonstrated that SIPE stabilized the oxidation-dependent Aβ42 aggregation, supporting an indirect anti-amyloidogenic mechanism mediated through preservation of DJ-1 redox homeostasis. Collectively, these findings position DJ-1 as a critical molecular node linking mitochondrial dysfunction, oxidative stress, neuroinflammation, and amyloidogenesis. Rather than representing a Parkinson’s disease-specific target, our findings support the concept that DJ-1 functions as a central redox-sensitive regulator of neurodegeneration whose preservation may simultaneously modulate several convergent pathogenic pathways involved in Alzheimer’s disease, including mitochondrial dysfunction, chronic oxidative stress, inflammatory activation, Aβ aggregation, and Tau pathology.

Finally, the attenuation of oxidative stress, neuroinflammation, DJ-1 oxidation, and amyloid pathology was accompanied by protection against two major downstream hallmarks of neurodegeneration: Tau hyperphosphorylation and apoptotic activation. TGC exposure induced a marked increase in Tau phosphorylation at Ser404 and Ser202/Thr205, indicating activation of tauopathic pathways by inflammatory, endocrine, and excitotoxic stressors. Since Tau hyperphosphorylation constitutes a critical event linking upstream cellular stress responses with cytoskeletal instability, impaired axonal transport, synaptic dysfunction, and neuronal death [75], these findings suggest that the pathological cascade initiated by chronic stress extended well beyond early oxidative alterations. Furthermore, phosphorylation at Ser404 and Ser202/Thr205 has been consistently associated with pathological Tau conformations and neurofibrillary pathology in experimental models of chronic stress [76]. Notably, SIPE effectively prevented Tau hyperphosphorylation, probably through coordinated suppression of oxidative stress, NF-κB activation, cytokine release, DJ-1 oxidation, and Aβ42 accumulation. Protection against Tau pathology was accompanied by a marked reduction in cleaved caspase-3 levels, indicating attenuation of apoptotic signaling. Given that caspase-3 activation represents a terminal execution step of programmed cell death and contributes to neuronal loss, synaptic dysfunction, and neurodegenerative progression [77], these findings suggest that SIPE interrupts the pathological transition from reversible cellular stress toward irreversible neuronal degeneration. Taken together, the present results support a mechanistic framework in which chronic stress initiates a progressive cascade involving mitochondrial dysfunction, oxidative stress, astrocytic reactivity, NF-κB activation, cytokine release, DJ-1 oxidation, amyloidogenesis, Tau pathology, and ultimately apoptotic cell death. Within this framework, SIPE acts as a multitarget neuroprotective intervention capable of interrupting several critical nodes of this interconnected pathogenic network.

Importantly, the biological relevance of the present findings was substantially strengthened through validation in three-dimensional neuron–ALC spheroids. Although conventional two-dimensional cultures provide valuable mechanistic insights, they inherently fail to fully reproduce the tissue-like architecture, cell–cell interactions, metabolic gradients, and microenvironmental complexity that characterize neural tissue in vivo [78]. Consequently, three-dimensional spheroid systems have emerged as more physiologically relevant platforms for evaluating neuroprotective interventions and modeling complex neurodegenerative processes [79]. Within this context, the beneficial actions of SIPE cannot be attributed solely to simplified cell culture conditions but appear sufficiently robust to preserve neuronal and glial homeostasis under conditions that more closely resemble the in vivo microenvironment. Accordingly, the molecular alterations identified in 2D cultures were consistently reproduced in the spheroid model. TGC exposure induced coordinated increases in NF-κB activation, DJ-1 oxidation, intracellular and extracellular Aβ42 accumulation, Tau phosphorylation, caspase-3 activation, and pro-inflammatory cytokine release. Such convergence is particularly noteworthy because it suggests that chronic stress does not trigger isolated pathological events but rather activates an interconnected pathogenic network involving oxidative stress, neuroinflammation, amyloidogenesis, tauopathy, and apoptotic signaling. In turn, SIPE significantly attenuated each of these pathological hallmarks, supporting the existence of a reproducible neuroprotective signature rather than a model-specific phenomenon. Among these findings, the preservation of DJ-1 redox homeostasis across both experimental platforms deserves particular attention. Throughout the present study, DJ-1 emerged as a potential molecular nexus linking mitochondrial dysfunction, oxidative stress, inflammation, amyloidogenesis, and neuronal degeneration in chronic stress.

Beyond corroborating the experimental findings, the phytochemical characterization and molecular docking analyses provide important mechanistic insights into the molecular basis underlying the neuroprotective activity of *Sesamum indicum* whole paste. Phytochemical profiling demonstrated that the paste is enriched in a diverse array of bioactive constituents, particularly the lignans (+)-sesamin and sesamolin, the phytosterols β-sitosterol and campesterol, and the antioxidant γ-tocopherol. Rather than acting as isolated entities, these compounds constitute a phytochemically integrated matrix with the potential to simultaneously modulate multiple pathogenic processes involved in chronic stress-associated neurodegeneration. Overall, the five compounds exhibited favorable interactions with TNF-α, DJ-1, Aβ42, and caspase-3, occupying functionally relevant binding cavities that substantially overlapped those recognized by established reference ligands. Although docking analyses do not constitute direct evidence of biological activity, the convergence of structurally distinct phytochemicals on common functional regions provides mechanistic support for the multitarget neuroprotective effects observed experimentally.

Among the evaluated phytochemicals, the lignans displayed the strongest interaction profiles. Interestingly, sesamolin consistently exhibited the highest binding affinities across the evaluated targets, surpassing the reference ligands for both TNF-α and DJ-1 while also demonstrating the strongest interaction with Aβ42 and maintaining high affinity for caspase-3. Sesamin likewise exhibited robust multitarget binding, particularly toward DJ-1 and caspase-3. The observation that two structurally related lignans independently interact with the same pathogenic proteins strongly suggests that lignans constitute a major mechanistic component of *Sesamum indicum* whole paste neuroprotection rather than identifying sesamin as the sole active principle. Particularly noteworthy was the remarkable conservation of interaction residues surrounding the highly redox-sensitive Cys106 region of DJ-1. Both lignans, together with β-sitosterol, campesterol, and γ-tocopherol, established interactions within this redox-regulatory microenvironment, supporting the hypothesis that preservation of DJ-1 structural integrity may represent one of the central mechanisms through which *Sesamum indicum* whole paste maintains mitochondrial function and antioxidant defenses under chronic stress conditions. Considering the growing evidence that DJ-1 acts as an upstream regulator of mitochondrial homeostasis, neuroinflammation, amyloidogenesis, and tau-related pathology, these findings further strengthen the biological relevance of DJ-1 as a convergent therapeutic target in chronic stress-associated neurodegeneration.

Likewise, the interaction profiles observed for Aβ42 indicate that multiple *Sesamum indicum* whole paste phytochemicals converge on the hydrophobic aggregation core of the peptide, a region critically involved in amyloid nucleation and fibrillization. This observation is fully consistent with the marked reduction in intracellular Aβ42 accumulation and inhibition of fibrillar growth demonstrated experimentally. Similarly, the shared occupation of functionally relevant regions within TNF-α and caspase-3 by several phytochemicals provides a plausible molecular explanation for the robust anti-inflammatory and anti-apoptotic responses observed in both two-dimensional neuron–astrocyte co-cultures and three-dimensional spheroids. Collectively, these findings support a mechanistic model in which the biological efficacy of *Sesamum indicum* whole paste emerges from the complementary and potentially cooperative interactions of multiple phytochemicals rather than from the activity of a single constituent. While sesamin and sesamolin appear to represent the principal molecular contributors owing to their consistently high binding affinities, the additional interactions provided by phytosterols and γ-tocopherol likely broaden the spectrum of biological targets, reinforcing the simultaneous modulation of oxidative stress, mitochondrial dysfunction, neuroinflammation, amyloidogenesis, and apoptosis. Such a multitarget phytochemical network provides a compelling mechanistic explanation for the robust neuroprotective effects of the complete SIPE preparation and highlights traditionally processed sesame paste as a promising source of naturally occurring compounds for the development of innovative interventions against chronic stress-associated neurodegeneration. Future studies should now focus on dissecting the individual and combinatorial contributions of these phytochemicals through comparative pharmacological studies, target-engagement analyses, pharmacokinetic characterization, blood–brain barrier permeability assessments, and structure–activity relationship studies. Such investigations will be essential to determine whether the complementary molecular interactions identified here translate into true biological synergy capable of reproducing the remarkable neuroprotective efficacy of the complete *Sesamum indicum* paste phytochemical matrix. Furthermore, although the present study provides a comprehensive mechanistic evaluation of *Sesamum indicum* whole paste within a preventive chronic stress paradigm, it did not include direct comparisons with currently approved Alzheimer’s disease therapies. Consequently, future investigations should also incorporate clinically established AD medications as comparative controls to determine the relative efficacy, mechanistic overlap, and translational potential of *Sesamum indicum* whole paste within the context of existing therapeutic strategies. Such comparative studies will represent an important step toward validating the clinical relevance of this multitarget phytochemical approach.

## 5. Conclusions

**Figure 12:**
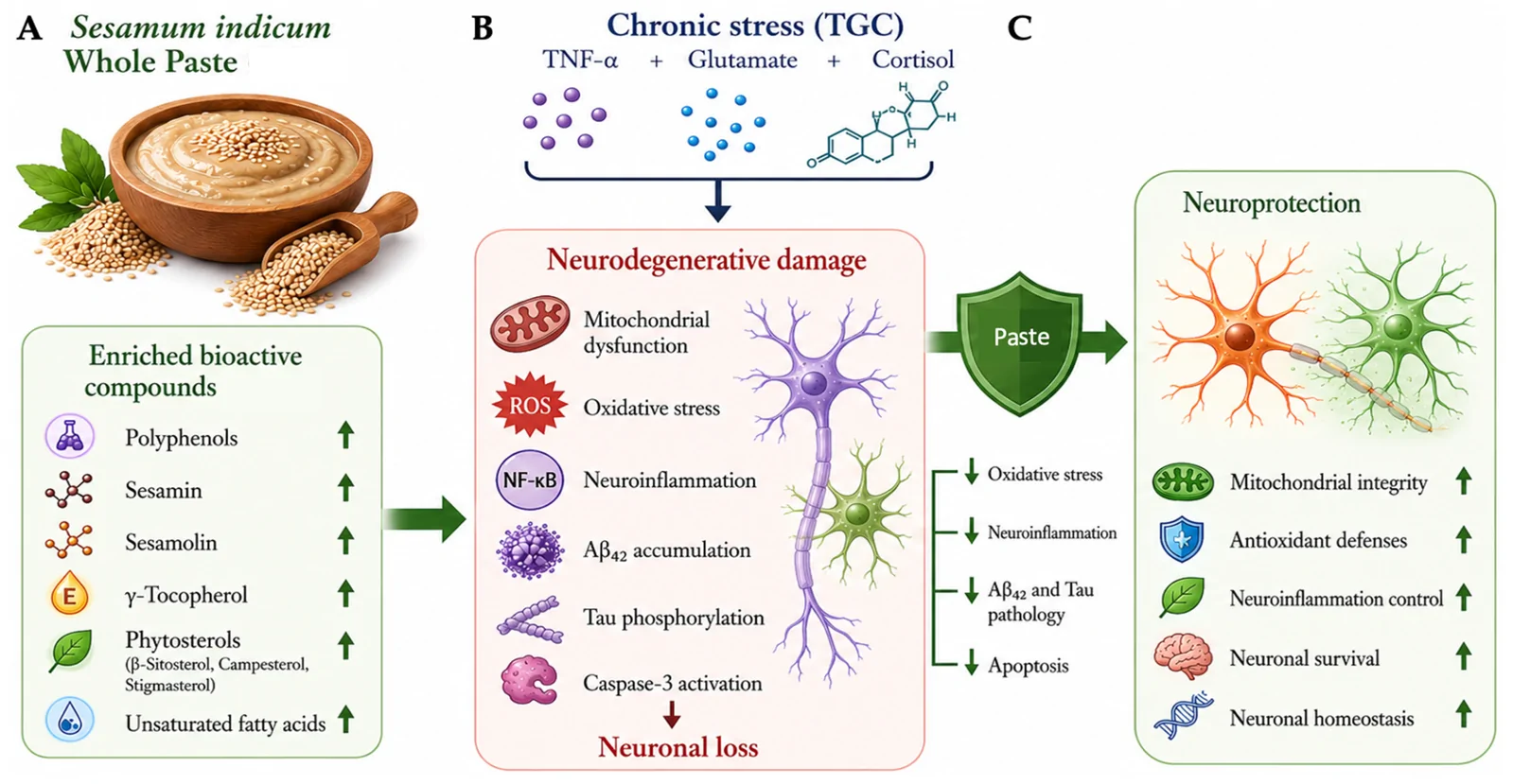
Integrated mechanistic model summarizing the neuroprotective actions of *Sesamum indicum* whole paste. (**A**) Traditional sesame paste, enriched in polyphenols, lignans, phytosterols, tocopherols, and unsaturated fatty acids, counteracts (**B**) chronic stress-induced neurodegenerative damage (TNF-α, glutamate, and cortisol), characterized by oxidative stress, mitochondrial dysfunction, neuroinflammation, Aβ42 accumulation, Tau pathology, and apoptosis. (**C**) Through a multitarget mechanism, sesame paste preserves neuron–astrocyte homeostasis, maintains mitochondrial integrity, enhances antioxidant defenses, reduces inflammatory and amyloidogenic processes, and promotes neuronal survival. Upward and downward arrows indicate increased or decreased levels of the corresponding molecule, respectively.

Collectively, the present study demonstrates that *Sesamum indicum* whole paste exerts robust multitarget neuroprotective effects against neurodegenerative alterations induced by a multifactorial stress paradigm integrating inflammatory, endocrine, and excitotoxic insults (Figure 12), by simultaneously attenuating mitochondrial dysfunction, oxidative stress, astrocytic reactivity, neuroinflammation, amyloidogenic processing, Tau phosphorylation, and apoptotic activation in both 2D neuron–glia co-cultures and 3D spheroid systems. Mechanistically, these protective effects appear to be associated with the coordinated action of multiple bioactive phytochemicals enriched within the whole paste preparation, particularly the lignans sesamin and sesamolin together with antioxidant-associated metabolites such as β-sitosterol, campesterol, and γ-tocopherol, which not only preserved neuronal and astrocytic homeostasis but also exhibited complementary interactions with molecular targets critically involved in inflammation, oxidative stress, amyloidogenesis, and apoptosis (Figure 12). Importantly, our findings support the emerging concept that chronic stress may function as a systems-level driver of Alzheimer’s disease-related pathology through progressive disruption of neuron–glia interactions and mitochondrial homeostasis. Within this framework, traditionally processed sesame-derived preparations may represent promising nutraceutical candidates capable of modulating interconnected pathogenic pathways involved in chronic stress-associated neurodegeneration. Beyond their biological relevance, these findings also highlight the potential scientific and translational value of ancestral food practices developed by communities historically exposed to chronic psychosocial stress, positioning *Sesamum indicum* whole paste as a compelling bridge between traditional knowledge, neurobiology, and future preventive strategies against neurodegenerative disease. Nevertheless, the present study was designed to investigate the preventive multitarget mechanisms of *Sesamum indicum* whole paste rather than to compare its efficacy with currently approved Alzheimer’s disease therapies. Therefore, although our findings provide a strong mechanistic rationale supporting the neuroprotective potential of *Sesamum indicum* whole paste, direct comparative studies with established AD medications will be essential to determine its relative therapeutic value and translational applicability. Such comparisons constitute an important limitation of the present work and represent a priority direction for future research.

## Figures and Tables

**Table 1:** Primary antibodies used for immunofluorescence and flow cytometry analyses, including target specificity, host species, commercial source, catalog number, and working dilution.

Protein	Target	Host	Catalog Number	Dilution	Manufacturer
βIII-tubulin	Neuronal marker	Mouse	14-4510-82	1:500	Thermo Fisher Scientific (eBioscience), Waltham, MA, USA
Nestin	Neuronal precursos marker	Mouse	MA1-5840	1:500	Invitrogen™, Thermo Fisher Scientific, Waltham, MA, USA
Sox2	Neuronal precursos marker	Rabbit	PA1-094	1:500	Thermo Fisher Scientific, Waltham, MA, USA
GFAP	Astrocyte marker	Rabbit	ab16997	1:500	Abcam, Cambridge, UK
S100β	Astrocyte marker	Mouse	Cat# 676604	1:200	BioLegend, San Diego, CA, USA)
Vimentin	Astrocyte marker	Rabbit	GTX16700	1:200	GeneTex Inc., Irvine, CA, USA
Pro-BDNF	Neurotrophic factor	Rabbit	P1374-200UL	1:500	Sigma-Aldrich, St. Louis, MO, USA
Glutamine Synthetase (GS)	Glutamate metabolism	Mouse	MA5-38531	1:500	Invitrogen™, Thermo Fisher Scientific, Waltham, MA, USA
β-Amyloid (1–42) Alexa Fluor™ 488-conjugated	Amyloid β (1–42)	Mouse (clone MOC64)	ab224026	1:200	Abcam, Cambridge, UK
Oxidized DJ-1 (oxC106)	Oxidative stress	Rabbit	ab169520	1:200	Abcam, Cambridge, UK
Phospho-Tau (Ser404)	Tau pathology	Rabbit	sc-12952	1:200	Santa Cruz Biotechnology, Dallas, TX, USA
Phospho-Tau (Ser202/Thr205)	Tau pathology	Mouse	clone AT8, Cat# MN1020	1:500	Invitrogen™, Thermo Fisher Scientific, Waltham, MA, USA
t-Tau	Total Tau Protein	Rabbit	T6402		Sigma-Aldrich, St. Louis, MO, USA
NF-κB (p65)	Inflammation	Rabbit	PA5-16545	1:500	Thermo Fisher Scientific, Waltham, MA, USA
Cleaved Caspase-3	Apoptosis	Rabbit	PA5-114687	1:500	Invitrogen™, Thermo Fisher Scientific, Waltham, MA, USA

**Table 2:** Total polyphenol content and fatty acid composition of sesame-derived products. Total polyphenols were quantified by the Folin–Ciocalteu method and expressed as mg gallic acid equivalents (GAE)/100 g sample. Fatty acid composition was determined by gas chromatography and expressed as percentage relative abundance ± S.D. GAE, gallic acid equivalents.

Parameter	Sesame Seed	Sesame Oil	Sesame Paste
Total Polyphenols (mg GAE/100 g sample)	77.138 ± 0.787	17.874 ± 0.164	96.029 ± 0.073
Linoleic acid (% relative area)	40.38 ± 0.41	42.04 ± 0.39	38.87 ± 0.19
Oleic acid (% relative area)	35.88 ± 0.37	35.43 ± 0.33	35.94 ± 0.18
Elaidic acid (% relative area)	0.89 ± 0.01	0.87 ± 0.01	0.84 ± 0.00
Palmitic acid (% relative area)	10.65 ± 0.11	10.57 ± 0.10	10.20 ± 0.05
Stearic acid (% relative area)	5.64 ± 0.06	5.74 ± 0.05	5.58 ± 0.03
Arachidic acid (% relative area)	0.56 ± 0.01	0.56 ± 0.01	0.52 ± 0.00

**Table 3:** Relative abundance of lignans, phytosterols, and tocopherols identified in sesame-derived products by GC–MS. Values correspond to chromatographic peak relative areas (% ± S.D) after ethyl acetate extraction, BSTFA derivatization, and identification using the NIST23 spectral library. ND: Not Detected.

Compound	Chemical Class	CAS	Seed (% Relative Area)	Whole Paste (% Relative Area)	Oil (% Relative Area)
Glycerol, 3TMS derivative	Polyol	6787-10-6	1.58 ± 0.11	ND	ND
1-Monoacetin, 2O-TMS	Glyceride	1000465-38-6	1.20 ± 0.01	ND	ND
Diacetin, 2-trimethylsilyl ether	Glyceride	1000465-83-5	0.44 ± 0.00	ND	ND
2,4-Decadienal (E,E)	Aldehyde	25152-84-5	ND	1.48 ± 0.01	ND
Sesamol	Phenolic lignan	17903-26-3	ND	00.13 ± 0.01	ND
2,4-Di-tert-butylphenoxytrimethylsilane	Phenolic derivative	53925-65-8	0.81 ± 0.00	ND	ND
Azelaic acid	Dicarboxylic acid	17906-08-0	ND	0.48 ± 0.01	ND
Sebacic acid	Dicarboxylic acid	18408-42-9	ND	0.35 ± 0.00	ND
Hexadecanenitrile derivative	Nitrile	629-79-8	ND	ND	0.43 ± 0.00
9-Octadecynenitrile	Nitrile	56599-96-3	ND	0.39 ± 0.00	0.67 ± 0.00
9-Octadecenenitrile (Z)	Nitrile	112-91-4	ND	0.39 ± 0.00	0.47 ± 0.00
Hexadecanoic acid derivative	Fatty acid derivative	112-39-0	ND	ND	0.29 ± 0.02
Palmitic acid	Saturated fatty acid	55520-89-3	28.49 ± 0.04	4.06 ± 0.01	9.71 ± 0.02
11,14-Octadecadienoic acid methyl ester	Unsaturated fatty acid	56554-61-1	0.83 ± 0.00	ND	ND
6-Octadecenoic acid methyl ester	Unsaturated fatty acid	2777-58-4	0.44 ± 0.00	ND	ND
Methyl 9-cis,11-trans-octadecadienoate	Unsaturated fatty acid	13058-52-1	ND	ND	0.50 ± 0.00
13-Octadecenoic acid methyl ester	Unsaturated fatty acid	56554-47-3	ND	ND	0.36 ± 0.01
Linoleic acid	ω-6 polyunsaturated fatty acid	56259-07-5	7.03 ± 0.01	8.70 ± 0.02	11.14 ± 0.01
Linoleic acid (secondary peak)	ω-6 polyunsaturated fatty acid	56259-07-5	0.28 ± 0.00	0.14 ± 0.00	0.42 ± 0.00
Oleic acid	ω-9 monounsaturated fatty acid	21556-26-3	2.03 ± 0.00	9.24 ± 0.01	10.38 ± 0.02
Oleic acid (secondary isomer)	ω-9 monounsaturated fatty acid	21556-26-3	0.97 ± 0.00	ND	ND
Elaidic acid	Trans fatty acid	96851-47-7	0.72 ± 0.00	0.22 ± 0.00	0.59 ± 0.01
11-Octadecenoic acid	Monounsaturated fatty acid	1000333-56-0	0.56 ± 0.00	ND	ND
Stearic acid	Saturated fatty acid	18748-91-9	6.03 ± 0.01	2.76 ± 0.01	3.98 ± 0.01
Arachidic acid	Saturated fatty acid	55530-70-6	2.23 ± 0.01	0.49 ± 0.00	1.01 ± 0.00
Behenic acid	Saturated fatty acid	74367-36-5	0.43 ± 0.01	0.14 ± 0.00	ND
Lignoceric acid	Saturated fatty acid	74367-37-6	0.24 ± 0.00	ND	ND
2-Palmitoylglycerol	Monoacylglycerol	53212-97-8	0.42 ± 0.00	ND	ND
1-Monopalmitin	Monoacylglycerol	1188-74-5	3.11 ± 0.01	0.71 ± 0.00	0.17 ± 0.00
2-Linoleoylglycerol	Monoacylglycerol	54284-46-7	3.89 ± 0.02	ND	ND
1-Monolinolein	Monoacylglycerol	54284-45-6	5.77 ± 0.01	ND	ND
2-Oleoylglycerol	Monoacylglycerol	56554-42-8	ND	0.29 ± 0.00	ND
1-Monooleoylglycerol	Monoacylglycerol	54284-47-8	0.76 ± 0.00	0.81 ± 0.00	0.19 ± 0.00
Glycerol monostearate	Monoacylglycerol	1188-75-6	1.92 ± 0.01	0.48 ± 0.00	ND
γ-Tocopherol	Tocopherol	2733-27-9	1.72 ± 0.02	3.84 ± 0.02	ND
(+)-Sesamin	Lignan	607-80-7	11.48 ± 0.12	24.70 ± 0.12	21.73 ± 0.20
Sesamolin	Lignan	526-07-8	3.45 ± 0.04	10.14 ± 0.05	7.62 ± 0.07
Campesterol	Phytosterol	55429-62-4	1.97 ± 0.02	3.87 ± 0.02	3.03 ± 0.03
Stigmasterol	Phytosterol	14030-29-6	0.93 ± 0.01	2.31 ± 0.01	1.81 ± 0.02
β-Sitosterol	Phytosterol	25873-56-7	5.17 ± 0.05	13.75 ± 0.07	10.85 ± 0.10
Isofucosterol	Phytosterol	22042-03-1	1.40 ± 0.01	2.63 ± 0.01	2.33 ± 0.02
Trimethylsiloxycitrostadienol	Phytosterol derivative	17774-68-4	ND	0.31 ± 0.01	ND

**Table 4:** Comparative molecular docking analysis of the principal bioactive constituents identified in *Sesamum indicum* whole paste against molecular targets associated with chronic stress-induced neurodegeneration. Docking simulations were performed using CB-Dock3 against TNF-α (PDB: 6RMJ), DJ-1 (PDB: 2OR3), amyloid-β42 (Aβ42; AlphaFold2 model), and caspase-3 (PDB: 1NME). The major SIPE phytochemicals evaluated included (+)-sesamin, sesamolin, β-sitosterol, campesterol, and γ-tocopherol. For each target, docking performance was compared with that of a previously characterized reference ligand. The table summarizes the predicted binding cavity (CurPocket ID), binding affinity (Vina score, kcal/mol), cavity volume, docking coordinates, docking box dimensions, and the principal amino acid residues involved in ligand–protein interactions. More negative Vina scores indicate stronger predicted binding affinity.

Submitted Receptor	Submitted Ligand	Vina Score	Contact Residues
TNF-α (6RMJ)	TNF-α inhibitor (CID: 16079006)	−8.0	Chain A: GLN102 ARG103 GLU104 THR105 PRO106 GLU107 Chain B: GLN102 ARG103 GLU104 THR105 PRO106 GLU107 GLU110 ALA111 LYS112 Chain C: ARG103 THR105 LYS112
	Sesamin (CID: 72307)	−7.7	Chain A: LYS98 SER99 PRO100 CYS101 GLN102 ARG103 GLU104 THR105 PRO106 GLU107 TYR115 GLU116 Chain B: CYS69 SER71 LYS98 SER99 PRO100 CYS101 GLN102 ARG103 GLU104 PRO106 GLU110 LYS112 GLU116 Chain C: LYS98 SER99 PRO100 CYS101 GLN102 ARG103 THR105 ALA111 LYS112 TYR115 GLU116
	Sesamolin (CID: 101746)	−9.0	Chain A: LYS98 SER99 PRO100 CYS101 GLN102 ARG103 GLU104 THR105 PRO106 GLU107 TYR115 GLU116 Chain B: CYS69 LYS98 SER99 PRO100 CYS101 GLN102 ARG103 GLU104 THR105 PRO106 GLU110 LYS112 GLU116 Chain C: CYS69 LYS98 SER99 PRO100 CYS101 GLN102 ARG103 THR105 ALA111 LYS112 TRP114 TYR115 GLU116 PRO117
	(−)-beta-Sitosterol (CID: 222284)	−6.2	Chain A: CYS69 LYS98 SER99 PRO100 CYS101 GLN102 ARG103 GLU104 THR105 PRO106 GLU107 TRP114 TYR115 GLU116 Chain B: CYS69 LYS98 SER99 PRO100 CYS101 GLN102 ARG103 GLU104 THR105 PRO106 GLU110 LYS112 TYR115 GLU116 Chain C: CYS69 LYS98 SER99 PRO100 CYS101 GLN102 ARG103 THR105 ALA111 LYS112 TRP114 TYR115 GLU116
	Campesterol (CID: 173183)	−6.9	Chain A: CYS69 SER71 LYS98 SER99 PRO100 CYS101 GLN102 ARG103 GLU104 THR105 PRO106 GLU107 GLY108 ALA109 TYR115 GLU116 Chain B: SER71 THR72 LYS98 SER99 PRO100 CYS101 GLN102 ARG103 GLU104 THR105 PRO106 GLU110 LYS112 TYR115 GLU116 Chain C: CYS69 LYS98 SER99 PRO100 CYS101 GLN102 ARG103 THR105 ALA111 LYS112 TRP114 TYR115 GLU116 PRO117
	(+)-gamma-Tocopherol (CID: 92729)	−6.9	Chain A: CYS69 LYS98 SER99 PRO100 CYS101 GLN102 ARG103 GLU104 THR105 PRO106 ALA109 GLU110 ALA111 LYS112 TRP114 TYR115 GLU116 Chain B: CYS69 HIS73 LYS98 SER99 PRO100 CYS101 GLN102 ARG103 TRP114 TYR115 GLU116 Chain C: CYS69 SER71 LYS98 SER99 PRO100 CYS101 GLN102 ARG103 GLU104 PRO113 TRP114 TYR115 GLU116
DJ-1 (2OR3)	8RK64 (CID: 162677482)	−7.0	Chain A: LYS12 GLY13 GLU15 GLU18 GLN45 CYS46 SER47 ARG48 ASP49 GLY75 ASN76 LEU77 GLY78 CYS106 ALA107 HIS126 LEU128 ALA129 PRO158 Chain B: ARG28 PRO184 LEU185
	Sesamin (CID: 72307)	−7.5	Chain A: LYS12 GLY13 GLU15 GLU18 GLN45 CYS46 SER47 ARG48 ASP49 GLY75 ASN76 LEU77 GLY78 ALA79 GLN80 SER83 GLU84 LYS89 CYS106 ALA107 THR110 ALA114 HIS126 LEU128 ALA129 ASP131 LYS132 PRO158 Chain B: ARG28 PRO184
	Sesamolin (CID: 101746)	−7.7	Chain A: LYS12 GLY13 GLU15 GLU18 GLN45 CYS46 SER47 ARG48 ASP49 GLY75 ASN76 LEU77 GLY78 ALA79 GLN80 SER83 GLU84 SER85 LYS89 CYS106 ALA107 THR110 ALA114 HIS115 HIS126 LEU128 ALA129 LYS132 PRO158 Chain B: ARG28 PRO184
	(−)-beta-Sitosterol (CID: 222284)	−5.6	Chain A: LYS12 GLY13 GLU15 GLU18 GLN45 CYS46 SER47 ARG48 ASP49 GLY75 ASN76 LEU77 GLN80 SER83 GLU84 LYS89 CYS106 THR110 ALA111 LEU113 ALA114 HIS115 GLU116 HIS126 LEU128 ALA129 LYS130 ASP131 LYS132 ASN135 GLY136 PRO158 Chain B: ARG28 PRO184
	Campesterol (CID: 173183)	−6.0	Chain A: LYS12 GLY13 GLU15 GLN45 CYS46 SER47 ARG48 ASP49 GLY75 ASN76 LEU77 GLN80 SER83 GLU84 LYS89 CYS106 THR110 ALA111 LEU113 ALA114 HIS115 HIS126 LEU128 ALA129 LYS130 ASP131 LYS132 ASN135 GLY136 Chain B: ARG28 PRO184
	(+)-gamma-Tocopherol (CID: 92729)	−5.6	Chain A: LYS12 GLY13 GLU15 GLU18 GLN45 CYS46 SER47 ARG48 ASP49 GLY74 GLY75 ASN76 LEU77 GLY78 ALA79 GLN80 CYS106 ALA107 THR110 HIS126 LEU128 LYS132 GLY157 PRO158 Chain B: ARG28 PRO184 LEU185 VAL186
Amyloid beta 42 (AlphaFold2)	Thioflavin T (CID: 16953)	−5.0	Chain A: VAL12 HIS13 HIS14 GLN15 LYS16 LEU17 PHE19 PHE20 ALA21 GLU22 ASP23 VAL24 ALA30 ILE31 ILE32 GLY33 LEU34 MET35 VAL36 VAL39 VAL40
	Sesamin (CID: 72307)	−5.0	Chain A: VAL12 HIS13 HIS14 GLN15 LYS16 LEU17 VAL18 PHE19 PHE20 ALA21 GLU22 ASP23 VAL24 GLY25 ALA30 ILE31 ILE32 GLY33 LEU34 MET35 VAL36
	Sesamolin (CID: 101746)	−6.2	Chain A: HIS14 GLN15 LYS16 LEU17 VAL18 PHE19 PHE20 ALA21 GLU22 ASP23 VAL24 GLY25 ALA30 ILE31 ILE32 GLY33 LEU34 MET35 VAL39 VAL40 ILE41
	(−)-beta-Sitosterol (CID: 222284)	−5.9	Chain A: TYR10 GLU11 VAL12 HIS13 HIS14 GLN15 LYS16 LEU17 PHE19 PHE20 ALA21 ILE31 ILE32 GLY33 LEU34 MET35 VAL36
	Campesterol (CID: 173183)	−5.8	Chain A: TYR10 GLU11 VAL12 HIS13 HIS14 GLN15 LYS16 LEU17 VAL18 PHE19 PHE20 ALA21 GLU22 ASP23 VAL24 ALA30 ILE31 ILE32 GLY33 LEU34 MET35 VAL36
	(+)-gamma-Tocopherol (CID: 92729)	−4.8	Chain A: HIS13 HIS14 GLN15 LYS16 LEU17 VAL18 PHE19 PHE20 ALA21 GLU22 ASP23 VAL24 ALA30 ILE31 ILE32 GLY33 LEU34 MET35 VAL36 VAL39 VAL40 ILE41 ALA42
Caspase-3 (INME)	NSCI (CID: 11591540)	−8.8	Chain A: THR62 SER63 ARG64 SER65 SER120 HIS121 GLN161 ALA162 CYS163 Chain B: TYR204 SER205 TRP206 ARG207 ASN208 SER209 LYS210 ASP211 GLY212 TRP214 GLN217 GLU246 PHE247 GLU248 SER249 PHE250 SER251 PHE252 PHE256
	Sesamin (CID: 72307)	−8.3	Chain B: TYR204 TRP206 ARG207 ASN208 SER209 LYS210 ASP211 TRP214 GLN217 GLU246 PHE247 GLU248 SER249 PHE250 SER251 PHE256
	Sesamolin (CID: 101746)	−8.1	Chain A: THR62 SER63 SER65 HIS121 Chain B: TYR204 SER205 TRP206 ARG207 ASN208 SER209 ASP211 TRP214 GLN217 LYS242 GLU246 PHE247 GLU248 SER249 PHE250 SER251 PHE252 ASP253 PHE256
	(−)-beta-Sitosterol (CID: 222284)	−6.3	Chain A: THR62 SER63 SER65 Chain B: TYR204 SER205 TRP206 ARG207 ASN208 SER209 ASP211 TRP214 GLN217 GLU246 PHE247 GLU248 SER249 PHE250 SER251 PHE252 ASP253 PHE256
	Campesterol (CID: 173183)	−6.8	Chain A: THR62 SER63 HIS121 Chain B: TYR204 TRP206 ARG207 ASN208 SER209 LYS210 ASP211 TRP214 GLN217 GLU246 PHE247 GLU248 SER249 PHE250 SER251 PHE252 ASP253 PHE256
	(+)-gamma-Tocopherol (CID: 92729)	−5.4	Chain A: THR62 ARG64 SER65 SER120 HIS121 GLY122 GLN161 CYS163 Chain B: TYR204 SER205 TRP206 ARG207 ASN208 SER209 ASP211 TRP214 GLN217 GLU246 PHE247 GLU248 SER249 PHE250 SER251 PHE252 ASP253 PHE256 HIS257

## Data Availability

All relevant data are within the manuscript.

## Supplementary Materials

The following supporting information can be downloaded at: https://www.mdpi.com/article/10.3390/biom16081084/s1.

## Abbreviations

The following abbreviations are used in this manuscript:

SIPE	*Sesamum indicum* whole paste extract
BDNF	Brain-Derived Neurotrophic Growth Factor
Aβ42	Amyloid Beta 42
TGC	TNFα, Glutamate, Cortisol (Hydrocortisone)
