# Supplementary material for: Neuroprotective Potential of Sesamum indicum in a Multifactorial In Vitro Neuron–Astrocyte System Exposed to Chronic Stress Mediators
Source: Biomolecules. 2026 Jul 24;16(8):1084. doi: 10.3390/biom16081084 (PMC13510025; doi:10.3390/biom16081084)
Supplement: Supplementary file 1 [file biomolecules-16-01084-s001.zip › biomolecules-4389874-supplementary.pdf]

Neuroprotective Potential of *Sesamum indicum* in a Multifactorial In Vitro Neuron-Astrocyte System Exposed to Chronic Stress Mediators

Viviana Soto-Mercado [1,*], María Paulina Arias-Loaiza [1,2] and Miguel Mendivil-Perez [1,2,*]

[1]Grupo de Neurosciencias de Antioquia, Faculty of Medicine, University of Antioquia, Calle 67 #53-108, University Research Headquarters, Calle 62#52-59, Building 1, Laboratory 411/412, Universidad de Antioquia (UdeA), Medellin 050010, Colombia

[2]Grupo de Investigación la Práctica de la Enfermería en el Contexto Social-GIPECS, Departamento Formación Básica Profesional, Facultad de Enfermería, Calle 67 #53-108, Universidad de Antioquia (UdeA), Medellin 050010, Colombia

*Correspondence: viviana.soto@udea.edu.co (V.S.-M.); miguel.mendivil@udea.edu.co (M.M.-P.);

Tel.: +57-604-219-6457 (V.S.-M.)

Supplementary Material

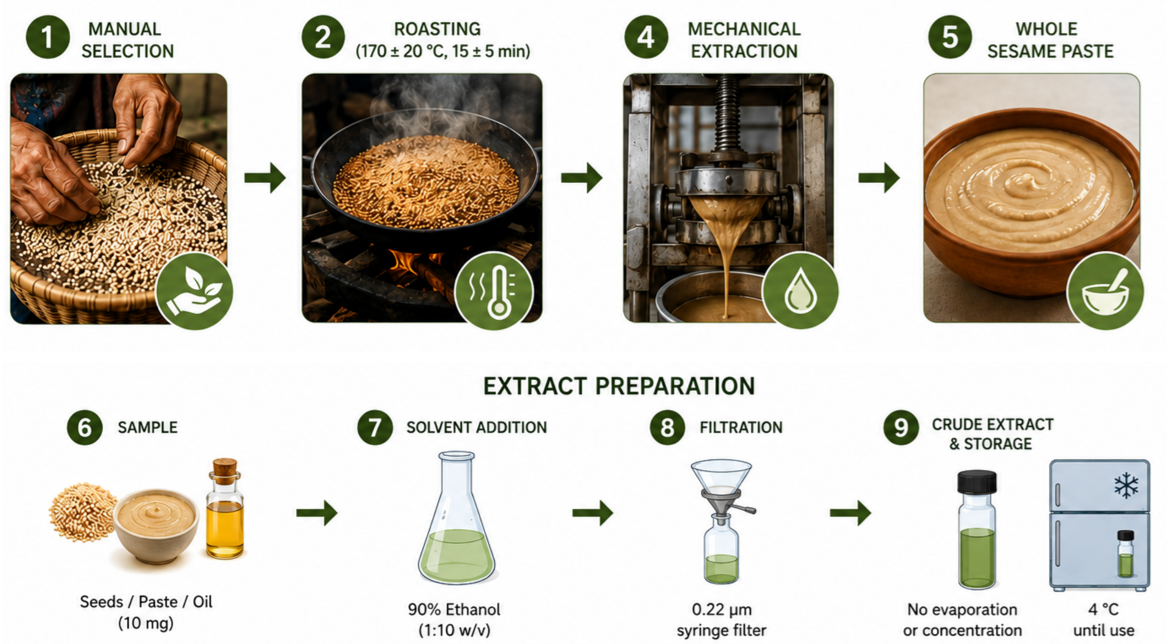

Suplementary Figure S1. Traditional production of sesame whole paste and preparation of crude ethanolic extracts. Sesame-derived products were obtained from small-scale farmers in the Montes de María region (Colombia) through traditional artisanal processing methods. Briefly, sesame seeds were manually selected (1), roasted under artisanal conditions (170 ± 20 °C, 15 ± 5 min) (2), and mechanically ground using traditional hand-operated milling systems (4) to obtain whole sesame paste (5). For extract preparation, sesame seeds, whole paste, and oil samples (6) were mixed with 90% ethanol at a 1:10 (w/v) ratio (7), filtered through a 0.22 µm syringe filter (8), and collected as crude ethanolic extracts without solvent evaporation or concentration before storage at 4 °C until use

(9). This workflow preserved the natural oil fraction and bioactive phytochemicals characteristic of traditionally processed sesame products used for subsequent in vitro analyses.

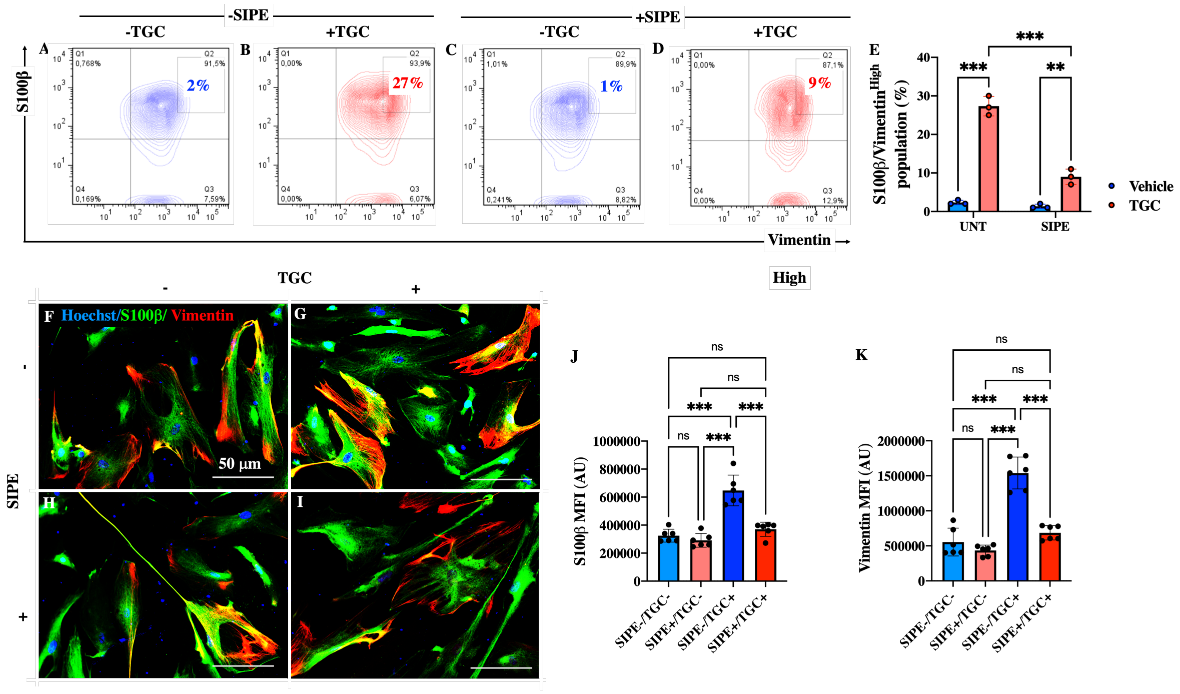

Supplementary Figure S2. ***Sesamum indicum*** **whole paste extract (SIPE) attenuates TGC-induced astrocytic reactivity, as evidenced by S100β and Vimentin expression in neuron–ALC co-cultures.** (A–D) Representative flow cytometry contour plots illustrating the proportion of S100β^{high}/Vimentin^{high} astrocyte-like cells (ALCs) in neuron–ALC co-cultures exposed to TNF-α/glutamate/cortisol (TGC) in the presence or absence of SIPE (10 µg/mL). (E) Quantitative analysis of the S100β^{high}/Vimentin^{high} ALC population determined by flow cytometry. (F–I) Representative fluorescence microscopy images showing S100β immunoreactivity (green), Vimentin immunoreactivity (red), and nuclear staining (blue) in neuron–ALC co-cultures exposed to vehicle, SIPE, TGC, or SIPE + TGC conditions. (J) Quantification of S100β mean fluorescence intensity (MFI) obtained from fluorescence microscopy analyses. (K) Quantification of Vimentin mean fluorescence intensity (MFI) obtained from fluorescence microscopy analyses. Data are presented as mean ± SD from at least three independent experiments. Statistical significance was determined by one-way ANOVA followed by appropriate post hoc analysis (*p < 0.05, **p < 0.01, ***p < 0.001). Images were acquired at 40× magnification. Scale bars are indicated in each panel.

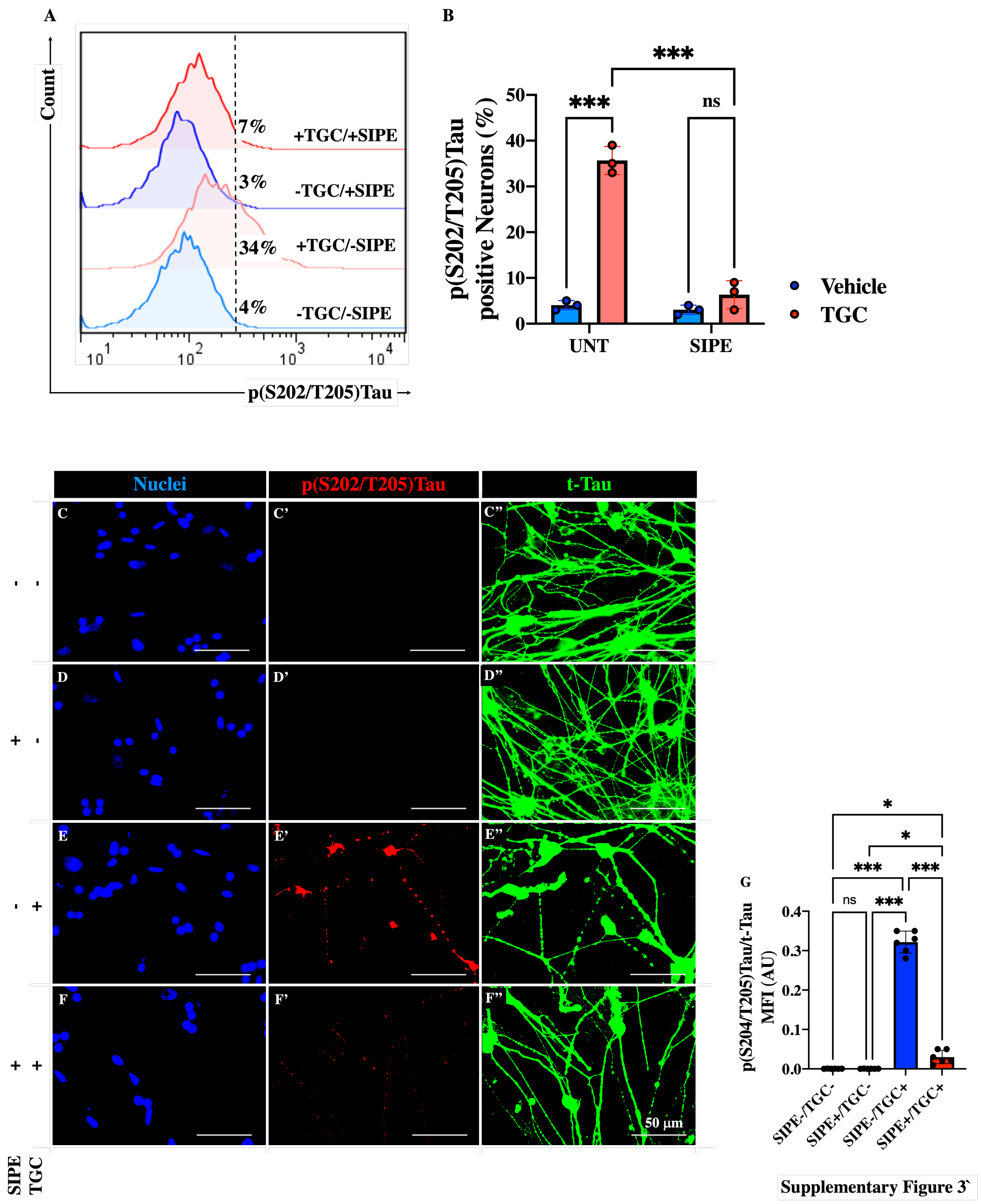

Supplementary Figure S3. Sesamum indicum whole paste extract (SIPE) prevents TGC-induced Tau phosphorylation at Ser202/Thr205 in neuron–ALC co-cultures.

(A) Representative flow cytometry histograms illustrating neuronal Tau phosphorylation, defined as p(S202/T205)Tau-positive cells, in neuron–ALC co-cultures exposed to TNF-α/glutamate/cortisol (TGC) in the presence or absence of SIPE (10 µg/mL). (B) Quantitative analysis of p(S202/T205)Tau-positive neuronal populations determined by flow cytometry. (C–F, C′–F′, C″–F″) Representative

fluorescence microscopy images showing nuclear staining (blue; C–F), phosphorylated Tau at Ser202/Thr205 (p(S202/T205)Tau, red; C′–F′), and total Tau (t-Tau, green; C″–F″) in neuron–ALC co-cultures exposed to vehicle, SIPE, TGC, or SIPE + TGC conditions. (G) Quantification of the p(S202/T205)Tau/t-Tau fluorescence intensity ratio, determined from fluorescence microscopy analyses. Data are presented as mean ± SD from at least three independent experiments. Statistical significance was determined by one-way ANOVA followed by appropriate post hoc analysis (*$p < 0.05$, **$p < 0.01$, ***$p < 0.001$). Images were acquired at 20× magnification. Scale bars are indicated in each panel.

Retention time (min)	Seed	Whole Paste	Oil
**6.13**	ND	0.37± 0.00	ND
**6.65–6.67**	ND	ND	1.96± 0.01
**7.63**	ND	0.23± 0.01	0.86± 0.22
**8.42–8.43**	ND	0.10± 0.00	1.21± 0.23
**9.27**	ND	0.12± 0.00	ND
**10.32–10.71**	0.40± 0.00	0.29± 0.01	ND
**12.46–12.47**	ND	0.18± 0.00	1.09± 0.11
**14.27**	0.27± 0.00	ND	ND
**17.71**	ND	0.17± 0.01	ND
**17.90**	ND	0.20± 0.01	ND
**23.87**	ND	0.22± 0.02	ND
**24.03**	ND	0.19± 0.00	ND
**28.32**	ND	0.69± 0.01	ND
**28.83**	ND	1.81± 0.12	ND
**31.01**	0.26± 0.00	ND	ND
**31.46–31.52**	ND	0.25± 0.01	0.15± 0.00
**31.69**	0.36± 0.00	ND	ND
**32.31–32.43**	0.24± 0.00	0.41± 0.01	ND
**32.73**	ND	0.18± 0.00	ND
**33.18–33.19**	ND	ND	0.16± 0.00
**33.74–33.77**	0.31± 0.00	0.22± 0.00	ND
**34.86–35.30**	ND	0.27± 0.00	ND
**36.22–36.23**	ND	ND	3.19± 0.01
**37.37–37.38**	ND	0.16± 0.00	0.34± 0.00
**38.80–38.90**	ND	0.24± 0.00	0.85± 0.03
**39.21–39.82**	ND	0.36± 0.00	0.29± 0.00
**40.48–40.68**	ND	0.22± 0.00	0.25± 0.00
**40.64**	ND	ND	0.14± 0.00
**40.87**	0.26± 0.00	ND	ND
**42.06–42.64**	0.21± 0.00	0.22± 0.00	0.19± 0.00
**43.62–43.72**	ND	0.26± 0.00	ND
**45.15**	0.34± 0.00	ND	ND
**47.08**	ND	ND	1.45± 0.01
**48.29**	ND	ND	0.23± 0.02

Supplementary Table S1. Unidentified chromatographic peaks detected in sesame seed, whole sesame paste, and sesame oil by GC–MS. Unidentified compounds are listed according to their chromatographic retention time (RT, min) and expressed as relative peak area (%±S.D.). Peaks that could not be confidently assigned to known metabolites based on comparison with the NIST23 spectral library are reported to provide a complete representation of the chromatographic profiles. ND indicates that no corresponding peak was detected in the respective sample.

Submitted Receptor	Submitted ligand	CurPocket ID	Vina score	Cavity volume (Å3)	Center (x, y, z)	Docking size (x, y, z)	Contact residues
**TNF- α (6RMJ)**	**TNF-a inhibitor (CID: 16079006)**	C1	-8.0	3405	-17, 51, 116	24, 24, 24	**Chain A: GLN102 ARG103 GLU104 THR105 PRO106 GLU107 Chain B: GLN102 ARG103 GLU104 THR105 PRO106 GLU107 GLU110 ALA111 LYS112 Chain C: ARG103 THR105 LYS112**
	**Sesamin (CID: 72307)**	C1	-7.7	3405	-17, 51, 116	24, 24, 24	**Chain A: LYS98 SER99 PRO100 CYS101 GLN102 ARG103 GLU104 THR105 PRO106 GLU107 TYR115 GLU116 Chain B: CYS69 SER71 LYS98 SER99 PRO100 CYS101 GLN102 ARG103 GLU104 PRO106**

						**GLU110**<br>**LYS112**<br>**GLU116**<br>**Chain C:**<br>**LYS98**<br>**SER99**<br>**PRO100**<br>**CYS101**<br>**GLN102**<br>**ARG103**<br>**THR105**<br>**ALA111**<br>**LYS112**<br>**TYR115**<br>**GLU116**
**Sesamolin (CID: 101746)**	C1	-9.0	3405	-17, 51, 116	24, 24, 24	**Chain A:**<br>**LYS98**<br>**SER99**<br>**PRO100**<br>**CYS101**<br>**GLN102**<br>**ARG103**<br>**GLU104**<br>**THR105**<br>**PRO106**<br>**GLU107**<br>**TYR115**<br>**GLU116**<br>**Chain B:**<br>**CYS69**<br>**LYS98**<br>**SER99**<br>**PRO100**<br>**CYS101**<br>**GLN102**<br>**ARG103**<br>**GLU104**<br>**THR105**<br>**PRO106**<br>**GLU110**<br>**LYS112**<br>**GLU116**<br>**Chain C:**<br>**CYS69**<br>**LYS98**<br>**SER99**<br>**PRO100**<br>**CYS101**

						**GLN102 ARG103 THR105 ALA111 LYS112 TRP114 TYR115 GLU116 PRO117**
**(-)-beta-Sitosterol (CID: 222284)**	C1	-6.2	3405	-17, 51, 116	25, 25, 25	**Chain A: CYS69 LYS98 SER99 PRO100 CYS101 GLN102 ARG103 GLU104 THR105 PRO106 GLU107 TRP114 TYR115 GLU116 Chain B: CYS69 LYS98 SER99 PRO100 CYS101 GLN102 ARG103 GLU104 THR105 PRO106 GLU110 LYS112 TYR115 GLU116 Chain C: CYS69 LYS98 SER99 PRO100 CYS101 GLN102 ARG103**

						**THR105**<br>**ALA111**<br>**LYS112**<br>**TRP114**<br>**TYR115**<br>**GLU116**
**Campesterol (CID: 173183)**	C1	-6.9	3405	-17, 51, 116	24, 24, 24	**Chain A:**<br>**CYS69**<br>**SER71**<br>**LYS98**<br>**SER99**<br>**PRO100**<br>**CYS101**<br>**GLN102**<br>**ARG103**<br>**GLU104**<br>**THR105**<br>**PRO106**<br>**GLU107**<br>**GLY108**<br>**ALA109**<br>**TYR115**<br>**GLU116**<br>**Chain B:**<br>**SER71**<br>**THR72**<br>**LYS98**<br>**SER99**<br>**PRO100**<br>**CYS101**<br>**GLN102**<br>**ARG103**<br>**GLU104**<br>**THR105**<br>**PRO106**<br>**GLU110**<br>**LYS112**<br>**TYR115**<br>**GLU116**<br>**Chain C:**<br>**CYS69**<br>**LYS98**<br>**SER99**<br>**PRO100**

							**CYS101<br>GLN102<br>ARG103<br>THR105<br>ALA111<br>LYS112<br>TRP114<br>TYR115<br>GLU116<br>PRO117**
	**(+)-gamma-Tocopherol (CID: 92729)**	C1	-6.9	3405	-17, 51, 116	22, 22, 22	**Chain A:<br>CYS69<br>LYS98<br>SER99<br>PRO100<br>CYS101<br>GLN102<br>ARG103<br>GLU104<br>THR105<br>PRO106<br>ALA109<br>GLU110<br>ALA111<br>LYS112<br>TRP114<br>TYR115<br>GLU116<br>Chain B:<br>CYS69<br>HIS73<br>LYS98<br>SER99<br>PRO100<br>CYS101<br>GLN102<br>ARG103<br>TRP114<br>TYR115<br>GLU116<br>Chain C:<br>CYS69<br>SER71<br>LYS98<br>SER99<br>PRO100**

							**CYS101**<br>**GLN102**<br>**ARG103**<br>**GLU104**<br>**PRO113**<br>**TRP114**<br>**TYR115**<br>**GLU116**
**DJ-1 (2OR3)**	**8RK64 (CID:162677482)**	C1	-7.0	1148	-6, -4, 12	25, 25, 25	**Chain A:**<br>**LYS12**<br>**GLY13**<br>**GLU15**<br>**GLU18**<br>**GLN45**<br>**CYS46**<br>**SER47**<br>**ARG48**<br>**ASP49**<br>**GLY75**<br>**ASN76**<br>**LEU77**<br>**GLY78**<br>**CYS106**<br>**ALA107**<br>**HIS126**<br>**LEU128**<br>**ALA129**<br>**PRO158**<br>**Chain B:**<br>**ARG28**<br>**PRO184**<br>**LEU185**
	**Sesamin (CID: 72307)**	C1	-7.5	1148	-6, -4, 12	24, 24, 24	**Chain A:**<br>**LYS12**<br>**GLY13**<br>**GLU15**<br>**GLU18**<br>**GLN45**<br>**CYS46**<br>**SER47**<br>**ARG48**<br>**ASP49**<br>**GLY75**<br>**ASN76**<br>**LEU77**

						**GLY78<br>ALA79<br>GLN80<br>SER83<br>GLU84<br>LYS89<br>CYS106<br>ALA107<br>THR110<br>ALA114<br>HIS126<br>LEU128<br>ALA129<br>ASP131<br>LYS132<br>PRO158<br>Chain B:<br>ARG28<br>PRO184**
	**Sesamolin (CID: 101746)**	C1	-7.7	1148	-6, -4, 12	24, 24, 24	**Chain A:<br>LYS12<br>GLY13<br>GLU15<br>GLU18<br>GLN45<br>CYS46<br>SER47<br>ARG48<br>ASP49<br>GLY75<br>ASN76<br>LEU77<br>GLY78<br>ALA79<br>GLN80<br>SER83<br>GLU84<br>SER85<br>LYS89<br>CYS106<br>ALA107<br>THR110<br>ALA114<br>HIS115<br>HIS126<br>LEU128<br>ALA129<br>LYS132**

						**PRO158**<br>**Chain B:**<br>**ARG28**<br>**PRO184**
**(-)-beta-Sitosterol (CID: 222284)**	C1	-5.6	1148	-6, -4, 12	25, 25, 25	**Chain A:**<br>**LYS12**<br>**GLY13**<br>**GLU15**<br>**GLU18**<br>**GLN45**<br>**CYS46**<br>**SER47**<br>**ARG48**<br>**ASP49**<br>**GLY75**<br>**ASN76**<br>**LEU77**<br>**GLN80**<br>**SER83**<br>**GLU84**<br>**LYS89**<br>**CYS106**<br>**THR110**<br>**ALA111**<br>**LEU113**<br>**ALA114**<br>**HIS115**<br>**GLU116**<br>**HIS126**<br>**LEU128**<br>**ALA129**<br>**LYS130**<br>**ASP131**<br>**LYS132**<br>**ASN135**<br>**GLY136**<br>**PRO158**<br>**Chain B:**<br>**ARG28**<br>**PRO184**

	**Campesterol (CID: 173183)**	C1	-6.0	1148	-6, -4, 12	24, 24, 24	**Chain A: LYS12 GLY13 GLU15 GLN45 CYS46 SER47 ARG48 ASP49 GLY75 ASN76 LEU77 GLN80 SER83 GLU84 LYS89 CYS106 THR110 ALA111 LEU113 ALA114 HIS115 HIS126 LEU128 ALA129 LYS130 ASP131 LYS132 ASN135 GLY136 Chain B: ARG28 PRO184**
	**(+)-gamma-Tocopherol (CID: 92729)**	C1	-5.6	1148	-6, -4, 12	22, 22, 22	**Chain A: LYS12 GLY13 GLU15 GLU18 GLN45 CYS46 SER47 ARG48 ASP49 GLY74 GLY75 ASN76 LEU77 GLY78**

							**ALA79 GLN80 CYS106 ALA107 THR110 HIS126 LEU128 LYS132 GLY157 PRO158 Chain B: ARG28 PRO184 LEU185 VAL186**
**Amyloid beta 42 (AlphaFold 2)**	**Thioflavin T (CID: 16953)**	C1	-5.0	109	2, -2, -1	22, 22, 22	**Chain A: VAL12 HIS13 HIS14 GLN15 LYS16 LEU17 PHE19 PHE20 ALA21 GLU22 ASP23 VAL24 ALA30 ILE31 ILE32 GLY33 LEU34 MET35 VAL36 VAL39 VAL40**
	**Sesamin (CID: 72307)**	C1	-5.0	109	2, -2, -1	24, 24, 24	**Chain A: VAL12 HIS13 HIS14 GLN15 LYS16 LEU17 VAL18 PHE19 PHE20 ALA21**

						**GLU22**<br>**ASP23**<br>**VAL24**<br>**GLY25**<br>**ALA30**<br>**ILE31**<br>**ILE32**<br>**GLY33**<br>**LEU34**<br>**MET35**<br>**VAL36**
**Sesamolin (CID: 101746)**	C1	-6.2	109	2, -2, -1	24, 24, 24	**Chain A:**<br>**HIS14**<br>**GLN15**<br>**LYS16**<br>**LEU17**<br>**VAL18**<br>**PHE19**<br>**PHE20**<br>**ALA21**<br>**GLU22**<br>**ASP23**<br>**VAL24**<br>**GLY25**<br>**ALA30**<br>**ILE31**<br>**ILE32**<br>**GLY33**<br>**LEU34**<br>**MET35**<br>**VAL39**<br>**VAL40**<br>**ILE41**
**(-)-beta-Sitosterol (CID: 222284)**	C1	-5.9	109	2, -2, -1	25, 25, 25	**Chain A:**<br>**TYR10**<br>**GLU11**<br>**VAL12**<br>**HIS13**<br>**HIS14**<br>**GLN15**<br>**LYS16**<br>**LEU17**<br>**PHE19**<br>**PHE20**<br>**ALA21**<br>**ILE31**<br>**ILE32**<br>**GLY33**

						LEU34<br>MET35<br>VAL36
**Campesterol (CID: 173183)**	C1	-5.8	109	2, -2, -1	24, 24, 24	**Chain A:**<br>**TYR10**<br>**GLU11**<br>**VAL12**<br>**HIS13**<br>**HIS14**<br>**GLN15**<br>**LYS16**<br>**LEU17**<br>**VAL18**<br>**PHE19**<br>**PHE20**<br>**ALA21**<br>**GLU22**<br>**ASP23**<br>**VAL24**<br>**ALA30**<br>**ILE31**<br>**ILE32**<br>**GLY33**<br>**LEU34**<br>**MET35**<br>**VAL36**
**(+)-gamma-Tocopherol (CID: 92729)**	C1	-4.8	109	2, -2, -1	22, 22, 22	**Chain A:**<br>**HIS13**<br>**HIS14**<br>**GLN15**<br>**LYS16**<br>**LEU17**<br>**VAL18**<br>**PHE19**<br>**PHE20**<br>**ALA21**<br>**GLU22**<br>**ASP23**<br>**VAL24**<br>**ALA30**<br>**ILE31**<br>**ILE32**<br>**GLY33**<br>**LEU34**<br>**MET35**<br>**VAL36**<br>**VAL39**

							**VAL40**<br>**ILE41**<br>**ALA42**
**Caspase-3 (INME)**	**NSCI (CID: 11591540)**	C1	-8.8	150	43, 99, 23	25, 25, 25	**Chain A:**<br>**THR62**<br>**SER63**<br>**ARG64**<br>**SER65**<br>**SER120**<br>**HIS121**<br>**GLN161**<br>**ALA162**<br>**CYS163**<br>**Chain B:**<br>**TYR204**<br>**SER205**<br>**TRP206**<br>**ARG207**<br>**ASN208**<br>**SER209**<br>**LYS210**<br>**ASP211**<br>**GLY212**<br>**TRP214**<br>**GLN217**<br>**GLU246**<br>**PHE247**<br>**GLU248**<br>**SER249**<br>**PHE250**<br>**SER251**<br>**PHE252**<br>**PHE256**
	**Sesamin (CID: 72307)**	C5	-8.3	150	43, 99, 23	24, 24, 24	**Chain B:**<br>**TYR204**<br>**TRP206**<br>**ARG207**<br>**ASN208**<br>**SER209**<br>**LYS210**<br>**ASP211**<br>**TRP214**<br>**GLN217**<br>**GLU246**<br>**PHE247**<br>**GLU248**

						**SER249 PHE250 SER251 PHE256**
**Sesamolin (CID: 101746)**	C5	-8.1	150	43, 99, 23	24, 24, 24	**Chain A: THR62 SER63 SER65 HIS121 Chain B: TYR204 SER205 TRP206 ARG207 ASN208 SER209 ASP211 TRP214 GLN217 LYS242 GLU246 PHE247 GLU248 SER249 PHE250 SER251 PHE252 ASP253 PHE256**
**(-)-beta-Sitosterol (CID: 222284)**	C5	-6.3	150	43, 99, 23	25, 25, 25	**Chain A: THR62 SER63 SER65 Chain B: TYR204 SER205 TRP206 ARG207 ASN208 SER209 ASP211 TRP214 GLN217 GLU246 PHE247 GLU248 SER249 PHE250**

						**SER251 PHE252 ASP253 PHE256**
**Campesterol (CID: 173183)**	C5	-6.8	150	43, 99, 23	24, 24, 24	**Chain A: THR62 SER63 HIS121 Chain B: TYR204 TRP206 ARG207 ASN208 SER209 LYS210 ASP211 TRP214 GLN217 GLU246 PHE247 GLU248 SER249 PHE250 SER251 PHE252 ASP253 PHE256**
**(+)-gamma-Tocopherol (CID: 92729)**	C5	-5.4	150	43, 99, 23	22, 22, 22	**Chain A: THR62 ARG64 SER65 SER120 HIS121 GLY122 GLN161 CYS163 Chain B: TYR204 SER205 TRP206 ARG207 ASN208 SER209 ASP211 TRP214 GLN217**

						**GLU246**<br>**PHE247**<br>**GLU248**<br>**SER249**<br>**PHE250**<br>**SER251**<br>**PHE252**<br>**ASP253**<br>**PHE256**<br>**HIS257**

Supplementary Table S2. Complete molecular docking parameters and ligand–protein interaction residues for the major bioactive constituents of *Sesamum indicum* whole paste. Complete molecular docking results for the principal bioactive constituents identified in *Sesamum indicum* whole paste extract (SIPE), including (+)-sesamin, sesamolin, β-sitosterol, campesterol, and γ-tocopherol. Docking simulations were performed using CB-Dock3 against TNF-*α* (PDB: 6RMJ), DJ-1 (PDB: 2OR3), amyloid-β42 (Aβ42; AlphaFold2 model), and caspase-3 (PDB: 1NME). For each molecular target, the corresponding reference ligand was included for comparative purposes. The table summarizes the predicted binding cavity (CurPocket ID), Vina binding affinity (kcal/mol), cavity volume ($Å^3$), docking center coordinates, docking box dimensions, and the complete list of amino acid residues involved in ligand–protein interactions. These data complement the representative docking analyses presented in Figure 11 and Table 4, providing the full interaction profiles supporting the proposed multitarget phytochemical mechanism of SIPE.
